# Synthesis of New Phenolic Derivatives of Quinazolin-4(3H)-One as Potential Antioxidant Agents—In Vitro Evaluation and Quantum Studies

**DOI:** 10.3390/molecules27082599

**Published:** 2022-04-18

**Authors:** Raluca Pele, Gabriel Marc, Anca Stana, Ioana Ionuț, Cristina Nastasă, Brîndușa Tiperciuc, Ilioara Oniga, Adrian Pîrnău, Laurian Vlase, Ovidiu Oniga

**Affiliations:** 1 Department of Pharmaceutical Chemistry, “Iuliu Hațieganu” University of Medicine and Pharmacy, 41 Victor Babeș Street, 400012 Cluj-Napoca, Romania; raluca.pele@umfcluj.ro (R.P.); stana.anca@umfcluj.ro (A.S.); ionut.ioana@umfcluj.ro (I.I.); cmoldovan@umfcluj.ro (C.N.); btiperciuc@umfcluj.ro (B.T.); ooniga@umfcluj.ro (O.O.); 2 Department of Pharmacognosy, “Iuliu Hațieganu” University of Medicine and Pharmacy, 12 Ion Creangă Street, 400010 Cluj-Napoca, Romania; ioniga@umfcluj.ro; 3 National Institute for Research and Development of Isotopic and Molecular Technologies, 400293 Cluj-Napoca, Romania; adrian.pirnau@itim-cj.ro; 4 Department of Pharmaceutical Technology and Biopharmaceutics, “Iuliu Hațieganu” University of Medicine and Pharmacy, 41 Victor Babeș Street, 400012 Cluj-Napoca, Romania; laurian.vlase@umfcluj.ro

**Keywords:** quinazolin-4(3H)-one, phenol derivatives, antioxidant, hybrid molecules, in vitro evaluation

## Abstract

Considering the important damage caused by the reactive oxygen (ROS) and nitrogen (RNS) species in the human organism, the need for new therapeutic agents, with superior efficacy to the known natural and synthetic antioxidants, is crucial. Quinazolin-4-ones are known for their wide range of biological activities, and phenolic compounds display an important antioxidant effect. Linking the two active pharmacophores may lead to an increase of the antioxidant activity. Therefore, we synthesized four series of new hybrid molecules bearing the quinazolin-4-one and phenol scaffolds. Their antioxidant potential was evaluated in vitro, considering different possible mechanisms of action: hydrogen atom transfer, ability to donate electrons and metal ions chelation. Theoretical quantum and thermodynamical calculations were also performed. Some compounds, especially the *ortho* diphenolic ones, exerted a stronger antioxidant effect than ascorbic acid and Trolox.

## 1. Introduction

Quinazolin-4(3H)-one is one of the heterocycles encountered and used in medicinal chemistry due to its wide spectrum of chemical and biological applicability. 2,3-Disubstituted-4-quinazolinones have been reported to possess antioxidant [1], anticancer [2], antihypertensive [3], antimicrobial, analgesic and anti-inflammatory [4] activities.

In terms of specific human metabolic processes, reactive oxygen (ROS) and nitrogen species (RNS) are generated in vivo; these can react with biological molecules like proteins, lipids, lipoproteins and DNA. Their high level can lead to the development of pathologies such as cancer or cardiovascular diseases. Therefore, the development of new therapeutic agents with superior efficacy to the known natural and synthetic antioxidants is important. Antioxidant compounds protect against ROS and RNS by releasing hydrogen atoms in order to neutralize these reactive radicals.

Considering the aspects presented above, an interesting profile is represented by quinazolin-4-one substituted with hydroxylated radicals in position 2. The published research of the antioxidant activity of these hybrid compounds is limited [1].

On the other hand, phenolic derivatives are a large class of natural or synthetic compounds known for their antioxidant or antimicrobial properties. Some examples of such natural phenolic compounds are: resveratrol, quercetin, kaempferol, luteolin, apigenin, catechin, caffeic, chlorogenic, rosmarinic acids, etc. Studies show that the number and position of phenolic groups are directly related to their antioxidant activity [5,6,7].

**Figure 1:**
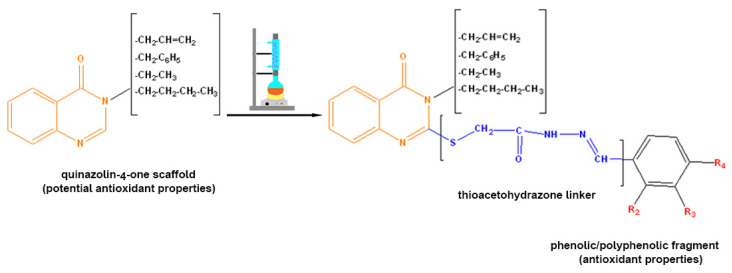
The hypothesis of this research.

This research is based on the hypothesis that linking two active pharmacophores [8] could lead to an increase of the antioxidant activity due to an additive effect. In our case, the linker between quinazolin-4-one and phenol derivatives is a thioacetohydrazone fragment (Figure 1). The choice of this linker was made because it has two useful features for this research. One is given by the flexibility of the thio-methylene fragment in the structure of the linker given by the sulfur atom and the sp3 carbon atom. Therefore, using the linker as a hinge would allow the folding of the quinazolin-4(3H)-one fragment and the polyphenolic fragment to modulate the polyphenol activity. The hydrazide–hydrazone moiety, represented by the other component of the linker structure with a rigid structure compared to the thio-methylene fragment, was chosen for its chelating properties on transition metal ions. The choice of this type of linker was made based on the multiple reports in the literature in which this type of compound is used to obtain complexes with transition metals [9,10,11].

In vitro spectrophotometric techniques are the most commonly used assays, because they are easily accessible, fast and simple. According to the literature, multiple quantum and thermodynamical parameters can describe the antioxidant activity of the compounds [12,13,14,15].

Herein, we present the synthesis, design and in vitro evaluation of the antioxidant activity of new quinazolin-4-one derivatives.

## 2. Results

### 2.1. Chemical Synthesis

**Figure 2:**
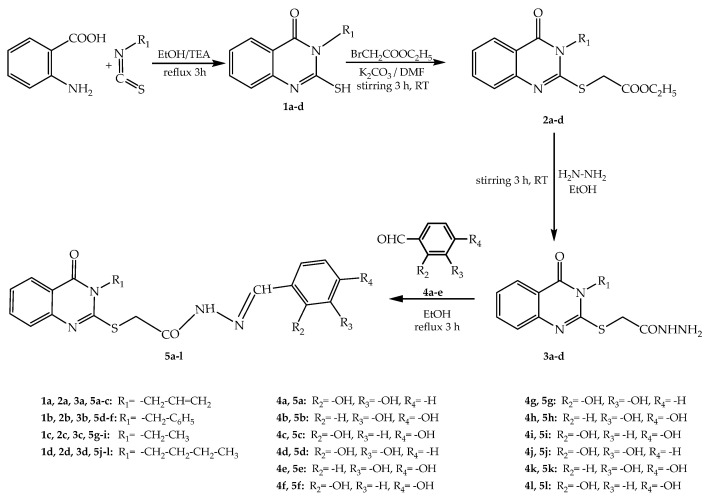
The synthesis steps followed in order to obtain compounds **5a**–**l**.

A total of 12 new final compounds **5a**–**l**, grouped in four series, were synthesized by the derivatization of 4-quinazolinon-2-mercapto-acetohydrazides **3a**–**d** and condensation with phenolic aromatic aldehydes **4a**–**e**. The 2-mercapto-quinazolin-4-one derivatives **1a**–**d** and the corresponding esters **2a**–**d** were synthesized according to the literature, starting from anthranilic acid (Figure 2) [16,17,18,19].

By reacting anthranilic acid with a variety of isothiocyanates, we obtained, in very good yields, the 2-mercapto-3-substituted quinazolin-4-ones **1a**–**d**. The esters **2a**–**d** were synthesized by the reaction of **1a**–**d** with ethyl bromoacetate in good yields. By stirring of the esters with hydrazine hydrate at room temperature, we obtained 4-quinazolinon-2-mercapto-acetohydrazides **3a**–**d**. The final synthesized compounds **5a**–**l** were obtained in good yields.

### 2.2. In Vitro Antioxidant, Antiradical and Chelation Assays

The antioxidant effect of compounds **5a**–**l** was evaluated in vitro based on the different mechanisms reported in the literature. Antioxidant compounds could manifest their activity by hydrogen atom transfer, electron transfer or by the chelation of transition metal ions [20]. The in vitro laboratory protocols used for the evaluation of the new compounds were performed at a semi-microscale level, according to the previous reports of our research group [21,22,23]. All determinations were performed in triplicate, and the results were presented as averages. All assays were performed using reference compounds mentioned individually in the experimental protocols.

#### 2.2.1. Antiradical Assays

The antiradical activity of compounds **5a**–**l** was evaluated spectrophotometrically by the investigation of the ABTS^+^, DPPH and NO**˙**, respectively, scavenging capacity. 

##### ABTS^+^ Radical Scavenging Assay

The results of the evaluation of the ABTS^+^ scavenging activity are presented in Table 1. Trolox and ascorbic acid were used as the positive controls. The most active compounds were **5h**, **5k** and **5l**, with lower IC_50_ values than that of the antioxidant reference drugs.

##### DPPH Radical Scavenging Assay

The results obtained in the DPPH radical scavenging assay are presented in Table 2. The reference antioxidants were ascorbic acid and Trolox. Compounds **5b**, **5h** and **5k** had the best activity. 

##### NO Radical Scavenging Assay

The NO antiradical activity of compounds **5a**–**l** and gentisic acid was evaluated spectrophotometrically based on the Griess reaction. The obtained results are presented in Table 3, revealing that compounds **5h** and **5j** had the best ability to scavenge this radical.

#### 2.2.2. Electron Transfer Assays

The antioxidant capacity of compounds **5a**–**l** expressed as the capacity of the donating electrons, was determined spectrophotometrically using the FRAP, TAC, RP and CUPRAC assays.

##### Ferric Reducing Antioxidant Power (FRAP)

In this assay, the tested compounds reduced ferric to ferrous ions, which formed a blue-colored complex (Fe^2+^-TPTZ) with tripyridyltriazine (2,4,6-tris(2-pyridyl)-*s*-triazine) at pH = 3.6. The amount of blue complex that resulted was proportional with the compounds’ capacity to reduce the Fe^3+^ ions. The results obtained for the FRAP assay are presented in Table 4. Compounds **5b**, **5g**, **5h** and **5k** expressed a good capacity for electron donation. 

##### Phosphomolybdate Assay for Total Antioxidant Capacity (TAC)

In the TAC assay, at acidic pH, the tested compounds reduced Mo^6+^ to Mo^5+^. The larger the amount of green Mo^5+^ phosphate complex, the higher the activity of the tested compounds was. The results of the TAC assay are presented in Table 4, and compounds **5a**, **5d**, **5g** and **5j** proved to have a very good electron-donating ability.

##### Reducing Power (RP) Assay

In this experiment, the tested compounds performed the reduction of ferricyanide to ferrocyanide to give Perl’s Prussian blue in the presence of ferric ions. The higher the percent of the reducing power, the higher the absorbance measured was. The results of the RP assay are presented in Table 4. Compounds **5b**, **5g**, **5h** and **5k** displayed their best reducing activity. 

##### Cupric Reducing Antioxidant Capacity (CUPRAC) Assay

In this assay, cupric ions were reduced to cuprous ions by electron donation. The cuprous ions that resulted were chelated by neocuproine, giving a colored complex with an absorbance proportional to the quantity of the resultant cuprous ions. The results of the CUPRAC assay are presented in Table 4 for the 3,4-dihydroxy derivatives (**5b**, **5e**, **5h** and **5k**), which displayed an excellent activity.

#### 2.2.3. Transition Metal Ions Chelation Assays

##### Fe^2+^ Chelation Assay

The chelating of the ferrous ion activity of the tested compounds was evaluated spectrophotometrically based on the competition for Fe^2+^ with ferrozine. A decrease in the resultant absorbance indicated that the ferrous ions were sequestered by the tested compounds. The results of the Fe^2+^ chelation assay are presented in Table 5. The chelating activity of compounds **5a**–**l** for the ferrous ions was compared to Na_2_-EDTA. The most active compounds were **5a**, **5d** and **5j**.

##### Cu^2+^ Chelation Assay

The cupric ion chelating capacity of the tested compounds was evaluated based on the competition for Cu^2+^ with murexide, and the results are presented in Table 6. The chelating activity was significant for compounds **5c, 5d** and **5g**, compared to Na_2_-EDTA. Analyzing the data obtained from this experiment, it can be seen that the most potent compounds were those with an OH group in position 2 of the aromatic radical.

### 2.3. Theoretical Quantum and Thermodynamical Calculations

The highest occupied molecular orbital (HOMO) indicated a good capacity for electron donation of the molecule, which is due to the susceptibility of a molecule to being attacked by electrophilic species. The energy of the lowest unoccupied molecular orbital (LUMO) was related to its susceptibility to being attacked by nucleophilic species and to its electron affinity.

**Figure 3:**
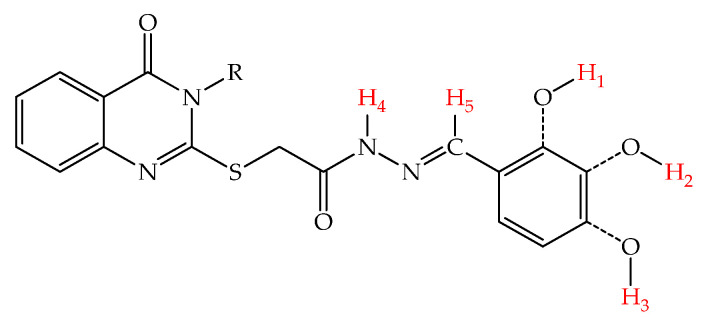
General structure representing the possible sites of molecules **5a**–**l** to release hydrogen atoms numbered H_1_–H_5_.

This assay is important to identify which functional group donates a hydrogen atom more easily and promotes the neutralization of free radicals. The easier a bond breaks, the easier the hydrogen is released. The general structure representing the possible sites of these molecules to release hydrogen atoms is presented in Figure 3.

The energy levels of the HOMO and LUMO are presented in Table 7, along with the bond dissociation energies (BDEs) of the molecule sites that could release hydrogen atoms numbered H_1_–H_5_, which followed after the in silico calculations.

The spin density maps of the compound radicals are presented in the Supplementary Materials (Table S1).

## 3. Discussion

### 3.1. Chemical Synthesis

The chemical synthesis started from anthranilic acid, which was subjected to a condensation reaction with four different isothiocyanate derivatives by reflux in ethanol to obtain the 4(3H)-quinazolinones **1a**–**d**.

The intermediate compounds **2a**–**d** were obtained by S-alkylation of the 2-mercapto-quinazolin-4(3H)-ones differently substituted in position 3 (compounds **1a**–**d**).

The hydrazinolysis of esters **2a**–**d** with hydrazine hydrate in order to obtain the (4-quinazolin-2-yl)-thioacetohydrazides **3a**–**d** was performed by stirring them in ethanol.

The final compounds **5a**–**l** were obtained by refluxing in ethanol the previously obtained hydrazides **3a**–**d** with the hydroxylated benzaldehydes **4a**–**e**.

The spectral data resulting from the analysis of the synthesized compounds were in accordance with the proposed structures.

In the MS spectra of the intermediate compounds **1a**–**d**, **2a**–**d**, **3a**–**d** and the final compounds **5a**–**l,** all the corresponding molecular peaks were found. The IR spectra analysis revealed all the desired signals for all the synthesized compounds: **1a**–**d**, **2a**–**d**, **3a**–**d** and **5a**–**l**. The phenolic signals found were νO–H stretching as wide bands between 3099 and 3487 cm^−1^. The hydrazide had N–H stretching bands between 3274 and 3279 cm^−1^ and hydrazine between 3470 and 3549 cm^−1^. In the 1628–1697 cm^−1^ spectral region, two strong νC = O stretching signals were found: one from 4(3H)-quinazolinone and another one from hydrazide or hydrazone. The νC = N signals for the hydrazide and hydrazone compounds were found between 1547 and 1558 cm^−1^. In the IR spectra, the difference between the parent compounds **1a**–**d,** the intermediate compounds **2a**–**d** and **3a**–**d** and their derivatives **5a**–**l** was the appearance, in all final compounds **5a**–**l**, of a specific signal between 1549 and 1558 cm^−1^ due to νC = N stretching and the presence of phenolic OH signals between 3099 and 3487 cm^−1^, proving that condensation had taken place successfully.

In the ^1^H-NMR spectra of the synthesized intermediate compounds and 4-quinazolinone derivatives, all proton expected signals were identified with the expected multiplicity.

In the ^13^C-NMR spectra of the synthesized compounds, the expected signals corresponding to the carbon atoms were identified in the expected region of the spectra.

The signals from the NMR spectra were attributed to the corresponding atoms and are presented in the characterizations of the compounds in the Chemistry subsection from the Materials and Methods section of the paper.

The graphic depiction of the recorded spectra of all the synthesized compounds are provided in the Supplementary Materials (Figures S1–S96).

### 3.2. In Vitro Antioxidant, Antiradical and Chelation Assay

#### 3.2.1. Antiradical Assays

The results obtained in the antiradical assays (ABTS^+^, DPPH and NO) were similar, displaying compounds **5h**, **5j** and **5k** as the most active phenolic 4(3H)-quinazolinone derivatives of the synthesized series of compounds, with higher radical scavenging activity than that of the reference antioxidants used. These compounds were *ortho* diphenolic derivatives. The rest of the compounds presented lower antiradical properties.

#### 3.2.2. Electron Transfer Assays

The results of the antioxidant activity evaluation (TAC and CUPRAC) revealed that phenolic derivatives **5a**, **5b**, **5d**, **5e**, **5g**, **5h**, **5j** and **5k** were the most active compounds of the newly synthesized series, presenting better antioxidant potential than ascorbic acid and Trolox, which were used as reference antioxidants. The rest of the synthesized compounds displayed a moderate-to-low antioxidant capacity. 

#### 3.2.3. Transition Metal Ions Chelation Assays

**5d** and **5j** were the most active compounds, but their activity was inferior to that of Na_2_-EDTA. Regarding the structure–activity relationship, the compounds that presented the best chelating properties were the 2,3-disubstituted catechol derivatives. Furthermore, compounds **5d** and **5j**, bearing bulky substituents, were surprisingly more active than **5a** and **5g**. Bulky benzyl or butyl substituents grafted onto the N_3_ atom of quinazolinone might stabilize better the resulting chelated complex in comparison to compounds **5a** and **5g**, which had smaller substituents.

### 3.3. Theoretical Quantum and Thermodynamical Energy Calculations

In all final compounds, the HOMO was found over the phenolic benzene, demonstrating that this was the molecular site mainly involved in radical scavenging and electron release. The LUMO was found in compounds **5c** and **5i** over the phenolic group, distributed over the phenolic and quinazolinone in compound **5l** and over quinazolinone in the rest of the compounds.

The HOMO energy levels were lower for the resorcinol-derived molecules (2,4-disubstituted series: **5c**, **5f**, **5i** and **5l**) but slightly higher for the catechol-derived molecules (2,3-disubstituted series: **5a**, **5d**, **5g** and **5j**; 3,4-disubstituted series: **5b**, **5e**, **5h** and **5k**). The lowest HOMO orbital was identified in compound **5c**, with an energy level of -5.69 eV, while the highest was identified in molecule **5k** at −5.43 eV. Regarding the LUMO, the lowest values were identified in the catechol-derived compounds (2,3-disubstituted series: **5a**, **5d**, **5g** and **5j**; 3,4-disubstituted series: **5b**, **5e**, **5h** and **5k**), while slightly higher levels were identified in the resorcinol-derived compounds (2,4-disubstituted series: **5c**, **5f**, **5i** and **5l**). The lowest energy levels for the LUMO were identified in compounds **5d** and **5g**, equal to −1.67 eV, while the highest was identified in molecule **5l** at −1.28 eV. Statistically comparing the energy levels in the two types of limit orbitals, a higher dispersion could be identified in the case of the LUMO (0.127 eV standard deviation) compared to the case of the HOMO (0.089 eV standard deviation).

Analyzing the HOMO–LUMO gap, considered a derivative descriptor that took into account both molecular frontier orbitals, high values could be observed in the case of the resorcinol-derived compounds, the highest being for compounds **5c**, **5i** and **5l** at over 4.2 eV. Interestingly, from the current enumeration of compounds with similar structures, resorcinol-derived compound **5f** was missing, its HOMO–LUMO gap falling in between the energetic gaps of its related compounds, **5d** and **5e**. This observation led us to analyze the intramolecular hydrogen-bonding effects that took place in our molecules, with strong influences on these compounds’ behavior. 

Lower values in the case of catechol derivatives were found for the HOMO–LUMO gap. More specifically, in the case of the 2,3-disubstituted compounds series, lower values were identified compared to the case of the 3,4-disubstituted compounds series, with values between 3.92 eV for compound **5j** and 3.78 eV for compound **5d**, this one being the smallest from the current series. 

The second important descriptor analyzed in this research for some groups that could release hydrogen atoms was BDE. The most susceptible group to release hydrogen atoms was *para* hydroxy (site H_3_), having globally the lowest BDEs in the whole series of compounds, followed by the OH groups from *meta* (site H_2_) and *ortho* (site H_1_), respectively. 

Moreover, the presence of the hydrazide–hydrazone group had a negative effect on the phenolic groups that could release hydrogen atoms, an effect seen especially in the OH group from the *ortho* position, the BDE of OH from H_1_ being much higher than the ones from H_2_ and H_3_. This could be explained by the intramolecular hydrogen bonding between one nitrogen atom from hydrazone and the phenol group from *ortho* (H_1_).

In the case of the hydrogen atoms bound to the nitrogen atom of the hydrazide group (H_4_ site), the lowest BDE in this series was 81.08 kcal/mol. The lowest BDE of the C-H bond on azomethine carbon (H_5_ site) was 99.78 kcal/mol. By analyzing the BDE values computed for the hydrazide–hydrazone group (sites H_4_ and H_5_), their high values indicated that the release of hydrogen atoms from the two sites was unlikely to take place. The BDE computed for the C-H bonds from the allyl group in the case of compounds **5a**, **5b** and **5c** was found to be more than 100 kcal/mol (data not presented), considering the allyl group inert in terms of hydrogen atom abstraction and the neutralization of an external radical.

The conformations with the lowest energy of the compounds are presented in Table 8. The resultant hydrogen bonds were also depicted. 

For all compounds that have an *ortho* hydroxy phenol group (**5a**, **5c**, **5d**, **5f**, **5g**, **5i**, **5j** and **5l**), a hydrogen bond could be found between the respective group as a donor and the imine nitrogen atom from hydrazine as the acceptor. This explained the higher BDE for the O-H bond described before caused by the resulting hydrogen bond generating a pseudo-bicyclic system.

In compounds **5b**, **5e**, **5h** and **5k**, a hydrogen bond appeared between the NH group from hydrazide as the donor and N_1_ atom from the quinazolinone heterocycle, this being strongly favored by the flexibility of the thio-methylene fragment, because the other elements of the newly resultant cyclic system were rigid. Surprisingly, from the resorcinol series of compounds, **5f** presented this internal hydrogen-bonding feature, being the only one in its series, an effect that could appear due to the bulky benzylidene fragment found on the N_3_ atom of the quinazolinone heterocycle. This intramolecular hydrogen bonding is important to be studied in compounds with this type of structure, because a bulky fragment such as benzylidene (inert in terms of antioxidant and/or antiradical properties) can induce conformational changes in molecules that can influence the antioxidant and/or antiradical properties of these compounds.

## 4. Materials and Methods

### 4.1. Chemistry

The reagents used for all the synthesis, purification, analysis and antioxidant assays were purchased from local suppliers and used in accordance with the instructions.

Using melting point device MPM-H1 (Schorpp Gerätetechnik, Überlingen, Germany), based on the glass capillary method, the melting points were measured. 

An Agilent 1100 series device was used to record the MS spectra of the compounds in the positive ionization mode for intermediate compounds **1a**–**d**, **2a**–**d** and **3a**–**d**, respectively, and an Agilent Ion Trap SL mass spectrometer (70 eV) instrument (Agilent Technologies, Santa Clara, CA, USA) in the negative and positive ionization modes for the final compounds **5a**–**l**. The IR spectra were recorded with a FT/IR 6100 spectrometer (Jasco, Cremella, Italy) in KBr pellets under vacuum. Using an Avance NMR spectrometer (Bruker, Karlsruhe, Germany) with dimethylsulfoxide-*d*_6_ (DMSO-*d*_6_), ^1^H-NMR and ^13^C-NMR spectra were recorded. Tetramethylsilane was used for the calibration of the spectrometer. The multiplicity identified for the signals was presented using the following abbreviations for the peak patterns: br—broad, s—singlet, d—doublet, dd—double doublet, t—triplet, td—triplet of doublets, q—quartet, quint—quintet, sext—sextet and m—multiplet, respectively. In order to ease the tracing of the signals given by the hydrogen or carbon atoms, some abbreviations were used to describe the location of the atom in a specific region of the molecule, as follows: Q-quinazolin-4(3H)-one, Bz-benzyl and Ar-phenolic benzene ring.

#### 4.1.1. Synthesis of Compounds **1a**–**d**

In a glass flask, 40 mL of absolute ethanol were added to 20 mmol (2.74 g) of anthranilic acid, 20 mmol of the appropriate isothiocyanate and 30 mmol of triethylamine. The mixture was refluxed gently under a condenser in a water bath for 3 h. The resultant precipitate was filtered and dried under vacuum. The impure solid was recrystallized from ethanol, resulting in a pure yellow solid. The followed protocol was an adaptation of one previously reported in the literature, where intermediate compounds **1a, 1b** and **1c** were previously reported [24,25].

*3-Allyl-2-mercaptoquinazolin-4(3H)-one* (**1a**): yellow solid; mp = 207 °C; yield = 53.38%; FT IR (KBr) ν_max_ cm^−1^: 1654.6 (C = N), 1623.2 (str C = O); MS: *m*/*z* = 219.0 (M + 1); ^1^H-NMR (DMSO-*d*_6_, 500 MHz) δ: 12.939 (br, 1H, -SH), 7.935 (dd, 1H, Q, *J* = 8 and 1.5 Hz), 7.724 (td, 1H, Q, *J* = 8 and 1.5 Hz), 7.381 (d, 1H, Q, *J* = 7.5 Hz), 7.315 (td, 1H, Q, *J* = 7.5 and 1 Hz), 5.909 (m, 1H, =CH-), 5.160 (dd, 1H, =CH_2_*, J* = 6.25 and 1.5 Hz), 5.132 (t, 1H, =CH_2_*, J* = 1.5 Hz), 5.032 (d, 2H, -CH_2_-, *J* = 5.5 Hz); ^13^C-NMR (DMSO-*d*_6_, 125 MHz) δ: 175.044 (C = O), 158.988 (C = N), 139.075 (Q), 135.463 (Q), 131.774 (-CH=), 127.267 (Q), 124.481 (Q), 117.125 (=CH_2_), 115.627 (Q), 47.601 (-CH_2_-).

*3-Benzyl-2-mercaptoquinazolin-4(3H)-one* (**1b**): yellow solid; mp = 253 °C; yield = 72.72%; FT IR (KBr) ν_max_ cm^−1^: 1687.8 (C = N), 1622.3 (str C = O); MS: *m*/*z* = 269.3 (M + 1); ^1^H-NMR (DMSO-*d*_6_, 500 MHz) δ: 13.060 (br, 1H, -SH), 7.943 (dd, 1H, Q, *J* = 8 and 1 Hz), 7.732 (td, 1H, Q, *J* = 8 and 1.5 Hz), 7.419 (d, 1H, Q, *J* = 7.5 Hz), 7.341-7.272 (m, 5H, Bz), 7.219 (t, 1H, Q, *J* = 7.5 Hz), 5.670 (s, 2H, -CH_2_-); ^13^C-NMR (DMSO-*d*_6_, 125 MHz) δ: 175.548 (C = N), 159.394 (C = O), 139.117 (Q), 136.597 (Bz), 135.582 (Q), 128.205 (Bz), 127.155 (Bz), 126.931 (Bz), 124.565 (Q), 115.403 (Q), 48.742 (-CH_2_-).

*3-Ethyl-2-mercaptoquinazolin-4(3H)-one* (**1c**): yellow solid; mp = 258 °C; yield = 74.33%; FT IR (KBr) ν_max_ cm^−1^: 1649.8 (C = N), 1621.8 (str C = O); MS: *m*/*z* = 207.2 (M + 1); ^1^H-NMR (DMSO-*d*_6_, 500 MHz) δ: 12.865 (br, 1H, -SH), 7.934 (dd, 1H, Q, *J* = 8 and 1 Hz), 7.711 (td, 1H, Q, *J* = 7.5 and 1.5 Hz), 7.364 (d, 1H, Q, *J* = 8.5 Hz), 7.307 (td, 1H, Q, *J* = 7.5 and 1 Hz), 4.439 (q, 2H, -CH_2_-, *J* = 7 Hz), 1.232 (t, 3H, –CH_3_, *J* = 7 Hz); ^13^C-NMR (DMSO-*d*_6_, 125 MHz) δ: 174.771 (C = N), 159.002 (C = O), 139.005 (Q), 135.344 (Q), 127.183 (Q), 124.418 (Q), 115.564 (Q), 41.015 (-CH_2_-), 11.954 (-CH_3_).

*3-Butyl-2-mercaptoquinazolin-4(3H)-one* (**1d**): yellow solid; mp = 177 °C; yield = 53.57%; FT IR (KBr) ν_max_ cm^−1^: 1650.2 (C = N), 1624.7 (str C = O); MS: *m*/*z* = 235.1 (M + 1); ^1^H-NMR (DMSO-*d*_6_, 500 MHz) δ: 12.891 (br, 1H, -SH), 7.942 (dd, 1H, Q, *J* = 8 and 1 Hz), 7.721 (td, 1H, Q, *J* = 8 and 1 Hz), 7.374 (d, 1H, Q, *J* = 8 Hz), 7.310 (td, 1H, Q, *J* = 7.5 and 1 Hz), 4.382 (q, 2H, -CH_2_-, *J* = 7.5 Hz), 1.653 (m, 2H, -CH_2_-), 1.338 (m, 2H, -CH_2_-), 0.919 (t, 3H, -CH_3_, *J* = 7.5 Hz); ^13^C-NMR (DMSO-*d*_6_, 125 MHz) δ: 175.002 (C = N), 159.212 (C = O), 139.033 (Q), 135.379 (Q), 127.246 (Q), 124.446 (Q), 115.571 (Q), 45.459 (-CH_2_-), 28.409 (-CH_2_-), 19.709 (-CH_2_-), 13.704 (-CH_3_).

#### 4.1.2. Synthesis of Compounds **2a**–**d**

In a glass flask, 25 mL of dimethylformamide (DMF) were added to 9.17 mmol of the appropriate quinazolin-4-one (**1a**–**d**), and 9.20 mmol of anhydrous potassium carbonate. The mixture was stirred at room temperature for 2 h. To the obtained reaction mixture, 9.17 mmol of ethyl bromoacetate was added gently, and the stirring was continued at room temperature for 7 h. The resultant precipitate was filtered and recrystallized from ethanol. A white solid was obtained. According to the literature reports, intermediate compounds **2a, 2b** and **2c** were previously synthesized by using another protocol [24,25].

*Ethyl 2-((3-allyl-4-oxo-3,4-dihydroquinazolin-2-yl)thio)acetate* (**2a**): white solid; mp = 75 °C; yield = 65.05%; FT IR (KBr) ν_max_ cm^−1^: 1743.8 (C = O), 1685 (C = O); MS: *m*/*z* = 305.3 (M + 1); ^1^H-NMR (DMSO-*d*_6_, 500 MHz) δ: 8.074 (dd, 1H, Q, *J* = 8 and 1.5 Hz), 7.796 (td, 1H, Q, *J* = 7.75 and 2 Hz), 7.477-7.428 (m, 2H, Q), 5.940 (m, 1H, -CH=), 5.236 (dd, 1H, =CH_2_, *J* = 10.5 and 1.5 Hz), 5.143 (dd, 1H, =CH_2_, *J* = 17.5 and 1 Hz), 4.718 (d, 2H, -CH_2_-, *J* = 5 Hz), 4.169-4.115 (m, 4H, -CH_2_- and -CH_2_-), 1.206 (t, 3H, -CH_3_, *J* = 7 Hz); ^13^C-NMR (DMSO-*d*_6_, 125 MHz) δ: 168.276 (C = O), 160.213 (C = O), 155.922 (C = N), 146.613 (Q), 134.889 (Q), 131.236 (-CH=), 126.532 (Q), 126.154 (Q), 125.762 (Q), 118.644 (Q), 117.573 (=CH_2_), 61.124 (-CH_2_-), 45.928 (-CH_2_-), 34.086 (-CH_2_-), 14.159 (-CH_3_).

*Ethyl 2-((3-benzyl-4-oxo-3,4-dihydroquinazolin-2-yl)thio)acetate* (**2b**): white solid; mp = 104 °C; yield = 93.11%; FT IR (KBr) ν_max_ cm^−1^: 1744.3 (C = O), 1679.6 (C = O); MS: *m*/*z* = 355.4 (M + 1); ^1^H-NMR (DMSO-*d*_6_, 500 MHz) δ: 8.110 (dd, 1H, Q, *J* = 8.25 and 1.5 Hz), 7.819 (td, 1H, Q, *J* = 7.25 and 1.5 Hz), 7.497-7.456 (m, 2H, Q), 7.363-7.323 (m, 2H, Bz), 7.298-7.269 (m, 3H, Bz), 5.341 (s, 2H, -CH_2_-), 4.156-4.098 (m, 4H, -CH_2_- and -CH_2_-), 1.194 (t, 3H, -CH_3_, *J* = 7 Hz); ^13^C-NMR (DMSO-*d*_6_, 125 MHz) δ: 168.199 (C = O), 160.758 (C = O), 156.153 (C = N), 146.641 (Q), 135.498 (Bz), 135.036 (Q), 128.611 (Bz), 127.512 (Bz), 126.805 (Bz), 126.658 (Q), 126.266 (Q), 125.818 (Q), 118.658 (Q), 61.124 (-CH_2_-), 47.013 (-CH_2_-), 34.191 (-CH_2_-), 14.138 (-CH_3_).

*Ethyl 2-((3-ethyl-4-oxo-3,4-dihydroquinazolin-2-yl)thio)acetate* (**2c**): white solid; mp = 88 °C; yield = 63.24%; FT IR (KBr) ν_max_ cm^−1^: 1739.0 (C = O), 1685.0 (C = O); MS: *m*/*z* = 293.2 (M + 1); ^1^H-NMR (DMSO-*d*_6_, 500 MHz) δ: 8.071 (dd, 1H, Q, *J* = 8 and 1.5 Hz), 7.761 (td, 1H, Q, *J* = 7.75 and 1.5 Hz), 7.469-7.415 (m, 2H, Q), 4.176-4.087 (m, 6H, -CH_2_-, -CH_2_- and -CH_2_-), 1.302 (t, 3H, -CH_3_, *J* = 7 Hz), 1.213 (t, 3H, -CH_3_, *J* = 7 Hz); ^13^C-NMR (DMSO-*d*_6_, 125 MHz) δ: 168.325 (C = O), 160.199 (C = O), 155.488 (C = N), 146.613 (Q), 134.763 (Q), 126.416 (Q), 126.077 (Q), 125.706 (Q), 118.777 (Q), 61.145 (-CH_2_-), 33.995 (-CH_2_-), 14.166 (-CH_3_), 13.004 (-CH_3_).

*Ethyl 2-((3-butyl-4-oxo-3,4-dihydroquinazolin-2-yl)thio)acetate* (**2d**): white solid; mp = 77 °C; yield = 54.12%; FT IR (KBr) ν_max_ cm^−1^: 1744.3 (C = O), 1679.6 (C = O); MS: *m*/*z* = 321.6 (M + 1); ^1^H-NMR (DMSO-*d*_6_, 500 MHz) δ: 8.067 (dd, 1H, Q, *J* = 7.5 and 1.5 Hz), 7.784 (td, 1H, Q, *J* = 7.75 and 1.5 Hz), 7.468-7.413 (m, 2H, Q), 4.172-4.130 (m, 4H, -CH_2_- and -CH_2_-), 4.056 (t, 2H, –CH_2_-, *J* = 7.5 Hz), 1.697 (m, 2H, -CH_2_-), 1.390 (m, 2H, -CH_2_-), 1.209 (t, 3H, -CH_3_, *J* = 5 Hz), 0.944 (t, 3H, -CH_3_, *J* = 7.5 Hz); ^13^C-NMR (DMSO-*d*_6_, 125 MHz) δ: 168.311 (C = O), 160.381 (C = O), 155.691 (C = N), 146.571 (Q), 134.770 (Q), 126.455 (Q), 126.091 (Q), 125.706 (Q), 118.714 (Q), 61.131 (-CH_2_-), 43.983 (-CH_2_-), 34.044 (-CH_2_-), 29.613 (-CH_2_-), 19.604 (-CH_2_-), 14.159 (-CH_3_), 13.571 (-CH_3_).

#### 4.1.3. Synthesis of Compounds **3a**–**d**

In a glass flask, 15 mL of ethanol 96% were added to 6 mmol of the appropriate ethyl quinazolin-4-one acetate (compounds **2a**–**d**) and 6 mmol of hydrazine hydrate >98%. The mixture was stirred for 5 h at room temperature. The resultant precipitate was filtered and dried under vacuum. The impure solid was recrystallized from absolute ethanol to obtain the target compound as a white solid. 

*2-((3-Allyl-4-oxo-3,4-dihydroquinazolin-2-yl)thio)acetohydrazide* (**3a**): white solid; mp = 153 °C; yield = 50.27%; FT IR (KBr) ν_max_ cm^−1^: 3276.9 (N–H hydrazide), 1681.6 (str C = O), 1657.5 (C = O), 1555.3 (C = N); MS: *m*/*z* = 291.2 (M + 1); ^1^H-NMR (DMSO-*d*_6_, 500 MHz) δ: 9.377 (br, 1H, -NH-), 8.082 (dd, 1H, Q, *J* = 8 and 1.5 Hz), 7.814 (td, 1H, Q, *J* = 7.75 and 1.5 Hz), 7.571 (d, 1H, Q, *J* = 7.5 Hz), 7.469 (td, 1H, Q, *J* = 8 and 1 Hz), 5.935 (m, 1H, -CH=), 5.225 (dd, 1H, =CH_2_, *J* = 10.5 and 1.5 Hz), 5.137 (dd, 1H, =CH_2_, *J* = 17.5 and 1 Hz), 4.728 (d, 2H, –CH_2_–, *J* = 5 Hz), 4.313 (br, 2H, -NH_2_), 3.971 (s, 2H, -CH_2_-); ^13^C-NMR (DMSO-*d*_6_, 125 MHz) δ: 166.183 (C = O), 160.346 (C = O), 156.139 (C = N), 146.732 (Q), 134.791 (Q), 131.390 (-CH=), 126.469 (Q), 126.056 (Q), 118.686 (Q), 117.538 (=CH_2_), 45.837 (-CH_2_-), 34.058 (-CH_2_-).

*2-((3-Benzyl-4-oxo-3,4-dihydroquinazolin-2-yl)thio)acetohydrazide* (**3b**): white solid; mp = 193 °C; yield = 53.97%; FT IR (KBr) ν_max_ cm^−1^: 3279.3 (N–H hydrazide), 1673.4 (str C = O), 1657.0 (C = O), 1547.5 (C = N); MS: *m*/*z* = 341.3 (M + 1); ^1^H-NMR (DMSO-*d*_6_, 500 MHz) δ: 9.371 (br, 1H, -NH-), 8.118 (d, 1H, Q, *J* = 7 Hz), 7.834 (t, 1H, Q, *J* = 8.5 Hz), 7.592 (d, 1H, Q, *J* = 8 Hz), 7.488 (t, 1H, Q, *J* = 7 Hz), 7.361-7.331 (m, 2H, Bz), 7.295-7.273 (m, 3H, Bz), 5.350 (s, 2H, -CH_2_-), 4.307 (br, 2H, -NH_2_), 3.957 (s, 2H, -CH_2_-); ^13^C-NMR (DMSO-*d*_6_, 125 MHz) δ: 166.022 (C = O), 160.842 (C = O), 156.335 (C = N), 146.718 (Q), 135.561 (Bz), 134.854 (Q), 128.569 (Bz), 127.407 (Bz), 126.749 (Bz), 126.553 (Q), 126.105 (Q), 126.056 (Q), 118.651 (Q), 46.852 (-CH_2_-), 34.184 (-CH_2_-).

*2-((3-Ethyl-4-oxo-3,4-dihydroquinazolin-2-yl)thio)acetohydrazide* (**3c**): white solid; mp = 169 °C; yield = 51.64%; FT IR (KBr) ν_max_ cm^−1^: 3274.5 (N–H hydrazide), 1675.3 (str C = O), 1658.4 (C = O), 1553.3 (C = N); MS: *m*/*z* = 279.2 (M + 1); ^1^H-NMR (DMSO-*d*_6_, 500 MHz) δ: 9.384 (br, 1H, -NH-), 8.070 (dd, 1H, Q, *J* = 8.25 and 1.5 Hz), 7.791 (td, 1H, Q, *J* = 7.75 and 1.5 Hz), 7.546 (d, 1H, Q, *J* = 7.5 Hz), 7.449 (td, 1H, Q, *J* = 7.5 and 1 Hz), 4.319 (br, 2H, -NH_2_), 4.111 (q, 2H, -CH_2_-, *J* = 7 Hz), 3.999 (s, 2H, -CH_2_-), 1.292 (t, 3H, -CH_3_, *J* = 7 Hz); ^13^C-NMR (DMSO-*d*_6_, 125 MHz) δ: 166.169 (C = O), 160.241 (C = O), 155.670 (C = N), 146.683 (Q), 134.553 (Q), 126.287 (Q), 125.930 (Q), 125.895 (Q), 118.770 (Q), 39.342 (-CH_2_-), 33.890 (-CH_2_-), 12.976 (-CH_3_).

*2-((3-Butyl-4-oxo-3,4-dihydroquinazolin-2-yl)thio)acetohydrazide* (**3d**): white solid; mp = 156 °C; yield = 50.12%; FT IR (KBr) ν_max_ cm^−1^: 3279.8 (N–H hydrazide), 1675.8 (str C = O), 1647.3 (C = O), 1554.8 (C = N); MS: *m*/*z* = 307.3 (M + 1); ^1^H-NMR (DMSO-*d*_6_, 500 MHz) δ: 9.383 (br, 1H, -NH-), 8.068 (dd, 1H, Q, *J* = 8.25 and 1.5 Hz), 7.793 (td, 1H, Q, *J* = 7.75 and 1.5 Hz), 7.548 (d, 1H, Q, *J* = 8.5 Hz), 7.451 (d, 1H, Q, *J* = 7.5 and 1 Hz), 4.320 (br, 2H, -NH_2_), 4.059 (t, 2H, -CH_2_-, *J* = 7.5 Hz), 3.986 (s, 2H, -CH_2_-), 1.688 (quint, 2H, -CH_2_-, *J* = 7 Hz), 1.387 (sext, 2H, -CH_2_-, *J* = 7.5 Hz), 0.964 (t, 3H, -CH_3_, *J* = 7.5 Hz); ^13^C-NMR (DMSO-*d*_6_, 125 MHz) δ: 166.148 (C = O), 160.416 (C = O), 155.845 (C = N), 146.641 (Q), 134.560 (Q), 126.329 (Q), 125.930 (Q), 118.707 (Q), 43.787 (-CH_2_-), 33.946 (-CH_2_-), 29.550 (-CH_2_-), 19.597 (-CH_2_-), 13.522 (-CH_3_).

#### 4.1.4. Synthesis of Compounds **5a**–**l**

In a glass flask, 8 mL of ethanol 96% were added to 2 mmol of the appropriate quinazoline-4-one acetohydrazide **3a**–**d**, 2 mmol of the appropriate dihydroxybenzaldehyde **4a**–**e** and a drop of glacial acetic acid. The mixture was refluxed gently under a condenser for 3 h. The resultant precipitate was filtered and dried under vacuum. The impure solid was recrystallized from dioxane, giving the pure product as a white or pale pink solid.

*2-((3-Allyl-4-oxo-3,4-dihydroquinazolin-2-yl)thio)-N’-(2,3-dihydroxybenzylidene)acetohydrazide* (**5a**): pale pink solid; mp = 192 °C; yield = 64.27%; FT IR (KBr) ν_max_ cm^−1^: 3470.2 (N–H hydrazone), 3420.1, 3152.5 (OH phenolic), 1697.0, 1646.9 (str C = O), 1558.6 (C = N); MS: *m*/*z* = 410.8 (M + 1); ^1^H-NMR (DMSO-*d*_6_, 500 MHz) δ: 8.446 (s, 1H, =CH-Ar), 8.079 (d, 1H, Q, *J* = 8 Hz), 7.807-7.740 (m, 1H, Q), 7.528-7.410 (m, 2H, Q), 6.979 (dd, 1H, Ar, *J* = 8 and 1.5 Hz), 6.838 (t, 1H, Ar, *J* = 8 Hz), 6.732 (t, 1H, Ar, *J* = 8 Hz), 5.954 (m, 1H, -CH=), 5.241 (m, 1H, =CH_2_), 5.160 (m, 1H, =CH_2_), 4.754 (m, 2H, -CH_2_-), 4.134 (s, 2H, -CH_2_-); ^13^C-NMR (DMSO-*d*_6_, 125 MHz) δ: 168.297 (C = O), 163.474 (C = O), 160.297 (C = N), 156.349 (N = CH-Ar), 156.146 (Ar-OH), 147.726 (Ar-OH), 146.697 (Q), 134.875 (Q), 131.334 (-CH=), 126.553 (Q), 126.147 (Q), 125.895 (Ar), 119.204 (Ar), 118.728 (Q), 117.699 (Ar), 117.580 (=CH_2_), 116.922 (Ar), 45.970 (-CH_2_-), 34.750 (-CH_2_-).

*2-((3-Allyl-4-oxo-3,4-dihydroquinazolin-2-yl)thio)-N’-(3,4-dihydroxybenzylidene)acetohydrazide* (**5b**): pale pink solid; mp = 235 °C; yield = 66.12%; FT IR (KBr) ν_max_ cm^−1^: 3519.4 (N–H hydrazone), 3420.1, 3183.9 (OH phenolic), 1658.9, 1650.2 (str C = O), 1558.6 (C = N); MS: *m*/*z* = 409.6 (M-1); ^1^H-NMR (DMSO-*d*_6_, 500 MHz) δ: 8.084-8.071 (m, 2H, Q and =CH-Ar), 7.768 (m, 1H, Q), 7.532-7.414 (m, 2H, Q), 7.215 (d, 1H, Ar, *J* = 2 Hz), 6.940 (d, 1H, Ar, *J* = 8 Hz), 6.774 (d, 1H, Ar, *J* = 8 Hz), 5.960 (m, 1H, -CH=), 5.240 (m, 1H, =CH_2_), 5.163 (d, 1H, =CH_2_, *J* = 17.5 and 1 Hz), 4.755 (m, 2H, -CH_2_-), 4.560 (s, 2H, -CH_2_-); ^13^C-NMR (DMSO-*d*_6_, 125 MHz) δ: 168.234 (C = O), 163.075 (C = O), 160.360 (C = N), 156.426 (N = CH-Ar), 147.831 (Ar-OH), 146.711 (Q), 145.724 (Ar-OH), 134.798 (Q), 131.390 (-CH=), 126.532 (Q), 126.014 (Q), 125.902 (Q), 125.594 (Ar), 120.688 (Ar), 118.672 (Q), 117.608 (=CH_2_), 115.641 (Ar), 112.743 (Ar), 45.921 (-CH_2_-), 35.037 (-CH_2_-).

*2-((3-Allyl-4-oxo-3,4-dihydroquinazolin-2-yl)thio)-N’-(2,4-dihydroxybenzylidene)acetohydrazide* (**5c**): pale pink solid; mp = 246 °C; yield = 57.21%; FT IR (KBr) ν_max_ cm^−1^: 3536.3 (N–H hydrazone), 3410.9, 3360.8 (OH phenolic), 1655.5, 1650.7 (str C = O), 1551.9 (C = N); MS: *m*/*z* = 409.3 (M-1); ^1^H-NMR (DMSO-*d*_6_, 500 MHz) δ: 8.346 (s, 1H, =CH-Ar), 8.081 (d, 1H, Q, *J* = 8 Hz), 7.781 (m, 1H, Q), 7.530-7.420 (m, 2H, Q), 7.320 (d, 1H, Ar, *J* = 8.5 Hz), 6.357 (m, 1H, Ar), 6.282 (m, 1H, Ar), 5.951 (m, 1H, -CH=), 5.239 (m, 1H, =CH_2_), 5.161 (m, 1H, =CH_2_), 4.749 (m, 2H, -CH_2_-), 4.104 (s, 2H, -CH_2_-); ^13^C-NMR (DMSO-*d*_6_, 125 MHz) δ: 167.842 (C = O), 162.998 (C = O), 160.751 (C = N), 160.479 (Ar-OH), 159.289 (N = CH-Ar), 158.113 (Ar-OH), 147.992 (Q), 134.847 (Q), 131.334 (-CH=), 131.152 (Ar), 126.546 (Q), 126.126 (Q), 125.902 (Q), 118.728 (Q), 117.678 (=CH_2_), 110.420 (Ar), 107.753 (Ar), 102.608 (Ar), 45.949 (-CH_2_-), 34.729 (-CH_2_-).

*2-((3-Benzyl-4-oxo-3,4-dihydroquinazolin-2-yl)thio)-N’-(2,3-dihydroxybenzylidene)acetohydrazide* (**5d**): pale pink solid; mp = 208 °C; yield = 62.24%; FT IR (KBr) ν_max_ cm^−1^: 3508.3 (N–H hydrazone), 3481.3, 3239.8 (OH phenolic), 1671.0, 1665.2 (str C = O), 1549.5 (C = N); MS: *m*/*z* = 459.3 (M-1); ^1^H-NMR (DMSO-*d*_6_, 500 MHz) δ: 8.440 (s, 1H, =CH-Ar), 8.117 (dd, 1H, Q, *J* = 8 and 1 Hz), 7.798 (m, 1H, Q), 7.550-7.439 (m, 2H, Q), 7.362-7.285 (m, 5H, Bz), 6.977 (dd, 1H, Ar, *J* = 8 and 1.5 Hz), 6.834 (m, 1H, Ar), 6.731 (t, 1H, Ar, *J* = 8 Hz), 5.370 (s, 2H, -CH_2_-), 4.114 (s, 2H, -CH_2_-); ^13^C-NMR (DMSO-*d*_6_, 125 MHz) δ: 168.248 (C = O), 163.397 (C = O), 160.856 (C = N), 156.580 (N = CH-Ar), 156.377 (Ar-OH), 147.705 (Ar-OH), 146.718 (Q), 135.561 (Bz), 135.015 (Q), 128.653 (Bz), 127.533 (Bz), 126.875 (Bz), 126.819 (Q), 126.679 (Q), 126.252 (Q), 125.944 (Ar), 119.806 (Ar), 118.770 (Q), 117.384 (Ar), 116.873 (Ar), 47.041 (-CH_2_-), 34.890 (-CH_2_-).

*2-((3-Benzyl-4-oxo-3,4-dihydroquinazolin-2-yl)thio)-N’-(3,4-dihydroxybenzylidene)acetohydrazide* (**5e**): pale pink solid; mp = 233 °C; yield = 65.11%; FT IR (KBr) ν_max_ cm^−1^: 3527.6 (N–H hydrazone), 3487.1, 3415.3 (OH phenolic), 1669.5, 1653.1 (str C = O), 1549.5 (C = N); MS: *m*/*z* = 459.2 (M-1); ^1^H-NMR (DMSO-*d*_6_, 500 MHz) δ: 8.113 (m, 1H, Q), 8.058 (s, 1H, =CH-Ar), 7.798 (m, 1H, Q), 7.556-7.440 (m, 2H, Q), 7.373-7.270 (m, 5H, Bz), 7.190 (d, 1H, Ar, *J* = 2 Hz), 6.929 (dd, 1H, Ar, *J* = 8 and 1.5 Hz), 6.761 (d, 1H, Ar, *J* = 8 Hz), 5.380 (s, 2H, -CH_2_-), 4.543 (s, 2H, -CH_2_-); ^13^C-NMR (DMSO-*d*_6_, 125 MHz) δ: 168.157 (C = O), 162.977 (C = O), 160.884 (C = N), 156.664 (N = CH-Ar), 147.810 (Ar-OH), 146.739 (Q), 145.689 (Ar-OH), 135.638 (Bz), 134.945 (Q), 128.604 (Bz), 127.470 (Bz), 126.847 (Bz), 126.644 (Q), 126.126 (Q), 125.559 (Q), 125.510 (Ar), 120.072 (Ar), 118.721 (Q), 115.627 (Ar), 112.785 (Ar), 47.027 (-CH_2_-), 35.156 (-CH_2_-).

*2-((3-Benzyl-4-oxo-3,4-dihydroquinazolin-2-yl)thio)-N’-(2,4-dihydroxybenzylidene)acetohydrazide* (**5f**): pale pink solid; mp = 253 °C; yield = 60.34; FT IR (KBr) ν_max_ cm^−1^: 3502.5 (N–H hydrazone), 3411.4, 3178.6 (OH phenolic), 1662.8, 1628.1 (str C = O), 1550.0 (C = N); MS: *m*/*z* = 461.2 (M + 1); ^1^H-NMR (DMSO-*d*_6_, 500 MHz) δ: 8.338 (s, 1H, =CH-Ar), 8.116 (d, 1H, Q, *J* = 7 Hz), 7.804 (m, 1H, Q), 7.551-7.452 (m, 2H, Q), 7.358-7.279 (m, 6H, 5H Bz and 1H Ar), 6.358-6.237 (m, 2H, Ar), 5.364 (s, 2H, -CH_2_-), 4.513 (s, 2H, -CH_2_-); ^13^C-NMR (DMSO-*d*_6_, 125 MHz) δ: 167.800 (C = O), 162.921 (C = O), 160.863 (C = N), 160.758 (Ar-OH), 160.471 (N = CH-Ar), 159.282 (Ar-OH), 147.978 (Q), 135.624 (Bz), 135.575 (Q), 128.653 (Bz), 127.533 (Bz), 126.875 (Bz), 126.812 (Q), 126.679 (Q), 126.238 (Q), 118.735 (Q), 110.420 (Ar), 107.753 (Ar), 102.608 (Ar), 47.027 (-CH_2_-), 34.876 (-CH_2_-).

*2-((3-Ethyl-4-oxo-3,4-dihydroquinazolin-2-yl)thio)-N’-(2,3-dihydroxybenzylidene)acetohydrazide* (**5g**): pale pink solid; mp = 229 °C; yield = 67.24%; FT IR (KBr) ν_max_ cm^−1^: 3545.4 (N–H hydrazone), 3480.8, 3414.8 (OH phenolic), 1689.8, 1678.2 (str C = O), 1550.4 (C = N); MS: *m*/*z* = 398.4 (M-1); ^1^H-NMR (DMSO-*d*_6_, 500 MHz) δ: 8.450 (s, 1H, =CH-Ar), 8.072 (d, 1H, Q, *J* = 8 Hz), 7.755 (m, 1H, Q), 7.441-7.427 (m, 2H, Q), 6.978 (d, 1H, Ar, *J* = 8 and 1.5 Hz), 6.836 (td, 1H, Ar, *J* = 8.5 and 1.5 Hz), 6.724 (t, 1H, Ar, *J* = 8 Hz), 4.148-4.128 (m, 2H, -CH_2_- and -CH_2_-), 1.319 (m, 3H, -CH_3_); ^13^C-NMR (DMSO-*d*_6_, 125 MHz) δ: 168.325 (C = O), 163.530 (C = O), 160.318 (C = N), 155.922 (N = CH-Ar), 155.726 (Ar-OH), 147.719 (Q), 146.690 (Ar-OH), 134.728 (Q), 126.427 (Q), 126.049 (Q), 125.937 (Ar), 125.832 (Q), 120.576 (Ar), 118.847 (Q), 117.384 (Ar), 39.342 (-CH_2_-), 34.618 (-CH_2_-), 13.046 (-CH_3_).

*2-((3-Ethyl-4-oxo-3,4-dihydroquinazolin-2-yl)thio)-N’-(3,4-dihydroxybenzylidene)acetohydrazide* (**5h**): pale pink solid; mp = 247 °C; yield = 62.67%; FT IR (KBr) ν_max_ cm^−1^: 3523.7 (N–H hydrazone), 3412.4, 3239.8 (OH phenolic), 1673.4, 1642.0 (str C = O), 1552.9 (C = N); MS: *m*/*z* = 397.1 (M-1); ^1^H-NMR (DMSO-*d*_6_, 500 MHz) δ: 8.085-8.054 (m, 2H, Q and =CH-Ar), 7.738 (m, 1H, Q), 7.506-7.386 (m, 2H, Q), 7.230 (d, 1H, Ar, *J* = 2 Hz), 6.944 (m, 1H, Ar), 6.784 (d, 1H, Ar, *J* = 8 Hz), 4.164-4.106 (m, 4H, -CH_2_- and -CH_2_-), 1.314 (m, 3H, -CH_3_); ^13^C-NMR (DMSO-*d*_6_, 125 MHz) δ: 168.290 (C = O), 163.166 (C = O), 106.339 (C = N), 155.992 (N = CH-Ar), 148.048 (Ar-OH), 147.845 (Q), 146.711 (Ar-OH), 134.623 (Q), 126.406 (Q), 125.986 (Q), 125.839 (Ar), 125.629 (Q), 120.709 (Ar), 118.798 (Q), 115.669 (Ar), 112.757 (Ar), 39.481 (-CH_2_-), 33.820 (-CH_2_-), 13.067 (-CH_3_).

*2-((3-Ethyl-4-oxo-3,4-dihydroquinazolin-2-yl)thio)-N’-(2,4-dihydroxybenzylidene)acetohydrazide* (**5i**): pale pink solid; mp = 153 °C; yield = 50.27%; FT IR (KBr) ν_max_cm^−1^: 3545.4 (N–H hydrazone), 3411.4, 3308.2 (OH phenolic), 1665.2, 1654.6 (str C = O), 1554.3 (C = N); MS: *m*/*z* = 397.3 (M-1); ^1^H-NMR (DMSO-*d*_6_, 500 MHz) δ: 8.352 (s, 1H, =CH-Ar), 8.070 (dd, 1H, Q, *J* = 8 and 1 Hz), 7.760 (m, 1H, Q), 7.509-7.396 (m, 3H, 2H Q and 1H Ar), 6.348 (dd, 1H, Ar, *J* = 8 and 2 Hz), 6.285 (dd, 1H, Ar, *J* = 8 and 2 Hz), 4.149-4.119 (m, 4H, -CH_2_- and -CH_2_-), 1.31 (t, 3H, -CH_3_, *J* = 7 Hz); ^13^C-NMR (DMSO-*d*_6_, 125 MHz) δ: 167.870 (C = O), 163.054 (C = O), 160.751 (C = N), 160.486 (N = CH-Ar), 160.262 (Ar-OH), 159.296 (Ar-OH), 147.999 (Q), 134.693 (Q), 131.159 (Ar), 126.413 (Q), 126.201 (Q), 125.839 (Q), 118.840 (Q), 110.420 (Ar), 107.746 (Ar), 102.615 (Ar), 33.890 (-CH_2_-), 13.039 (-CH_2_-).

*2-((3-Butyl-4-oxo-3,4-dihydroquinazolin-2-yl)thio)-N’-(2,3-dihydroxybenzylidene)acetohydrazide* (**5j**): pale pink solid; mp = 209 °C; yield = 50.27%; FT IR (KBr) ν_max_ cm^−1^: 3522.8 (N–H hydrazone), 3466.9, 3420.1 (OH phenolic), 1693.1, 1676.8 (str C = O), 1550.4 (C = N); MS: *m*/*z* = 425.7 (M-1); ^1^H-NMR (DMSO-*d*_6_, 500 MHz) δ: 8.450 (s, 1H, =CH-Ar), 8.076 (d, 1H, Q, *J* = 7.5 Hz), 7.756 (m, 1H, Q),7.508-7.387 (m, 2H, Q), 6.979 (dd, 1H, Ar, *J* = 7.5 and 1.5 Hz), 6.835 (td, 1H, Ar, *J* = 8.75 and 1.5 Hz), 6.733 (t, 1H, Ar, *J* = 8 Hz), 4.144 (s, 2H, -CH_2_-), 4.085 (m, 2H, -CH_2_-), 1.716 (m, 2H, -CH_2_-), 1.399 (m, 2H, -CH_2_-), 0.960 (t, 3H, -CH_3_, *J* = 7.5 Hz); ^13^C-NMR (DMSO-*d*_6_, 125 MHz) δ: 168.325 (C = O), 163.523 (C = O), 160.443 (C = N), 156.111 (N = CH-Ar), 155.915 (Ar-OH), 147.719 (Ar-OH), 145.570 (Q), 134.735 (Q), 126.469 (Q), 126.063 (Q), 125.958 (Q), 125.832 (Ar), 120.583 (Ar), 118.777 (Q), 117.384 (Ar), 116.901 (Ar), 43.990 (-CH_2_-), 33.820 (-CH_2_-), 29.641 (-CH_2_-), 19.660 (-CH_2_-), 13.592 (-CH_3_).

*2-((3-Butyl-4-oxo-3,4-dihydroquinazolin-2-yl)thio)-N’-(3,4-dihydroxybenzylidene)acetohydrazide* (**5k**): pale pink solid; mp = 238 °C; yield = 50.27%; FT IR (KBr) ν_max_ cm^−1^: 3530.5 (N–H hydrazone), 3169.4, 3099.5 (OH phenolic), 1660.4, 1649.8 (str C = O), 1553.8 (C = N); MS: *m*/*z* = 425.2 (M-1); ^1^H-NMR (DMSO-*d*_6_, 500 MHz) δ: 8.073-8.058 (m, 2H, Q and =CH-Ar), 7.754 (m, 1H, Q), 7.442-7.390 (m, 2H, Q), 7.214 (d, 1H, Ar, *J* = 2 Hz), 6.932 (m, 1H, Ar), 6.770 (d, 1H, Ar, *J* = 8 Hz), 4.567 (s, 2H, -CH_2_-), 4.098 (m, 2H, -CH_2_-), 1.716 (m, 2H, -CH_2_-), 1.396 (m, 2H, -CH_2_-), 0.945 (m, 3H, -CH_3_); ^13^C-NMR (DMSO-*d*_6_, 125 MHz) δ: 168.248 (C = O), 163.110 (C = O), 160.507 (C = N), 156.188 (N = CH-Ar), 147.810 (Ar-OH), 146.662 (Q), 145.717 (Ar-OH), 134.658 (Q), 126.441 (Q), 126.021 (Q), 125.930 (Q), 125.832 (Ar), 118.777 (Ar), 118.728 (Q), 115.627 (Ar), 112.743 (Ar), 43.920 (-CH_2_-), 33.841 (-CH_2_-), 29.634 (-CH_2_-), 19.660 (-CH_2_-), 13.585 (-CH_3_).

*2-((3-Butyl-4-oxo-3,4-dihydroquinazolin-2-yl)thio)-N’-(2,4-dihydroxybenzylidene)acetohydrazide* (**5l**): pale pink solid; mp = 277 °C; yield = 50.27%; FT IR (KBr) ν_max_cm^−1^: 3502.1 (N–H hydrazone), 3256.2, 3184.8 (OH phenolic), 1664.2, 1642.0 (str C = O), 1554.8 (C = N); MS: *m*/*z* = 426.6 (M + 1); ^1^H-NMR (DMSO-*d*_6_, 500 MHz) δ: 8.351 (s, 1H, =CH-Ar), 8.066 (dd, 1H, Q, *J* = 8 and 1 Hz), 7.759 (m, 1H, Q), 7.493 (t, 1H, Q, *J* = 7.5 Hz), 7.438 (m, 1H, Q), 7.318 (d, 1H, Ar, *J* = 9 Hz), 6.348 (dd, 1H, Ar, *J* = 9 and 2 Hz), 6.276 (dd, 1H, Ar, *J* = 8.5 and 2 Hz), 4.114-4.058 (m, 4H, -CH_2_- and -CH_2_-), 1.706 (m, 2H, -CH_2_-), 1.391 (m, 2H, -CH_2_-), 0.952 (m, 3H, -CH_3_); ^13^C-NMR (DMSO-*d*_6_, 125 MHz) δ: 167.870 (C = O), 163.054 (C = O), 160.758 (C = N), 160.443 (Ar-OH), 159.296 (N = CH-Ar), 158.132 (Ar-OH), 147.999 (Q), 134.700 (Q), 131.159 (Ar), 126.462 (Q), 126.035 (Q), 125.839 (Q), 118.784 (Q), 110.420 (Ar), 107.753 (Ar), 102.615 (Ar), 43.962 (-CH_2_-), 33.932 (-CH_2_-), 29.634 (-CH_2_-), 19.653 (-CH_2_-), 13.585 (-CH_3_).

### 4.2. In Vitro Antioxidant, Antiradical and Chelation Assays

The stock solutions of the reference antioxidants and of the tested compounds were prepared by dissolving the solid powders of the compounds in DMSO, with resulting concentrations of 1 mg/mL. The absorbance of the samples was determined in low-volume single-use 10 mm width cuvettes using an UV–Vis Jasco V-530 spectrophotometer (Jasco International Co., Tokyo, Japan). The absorption spectra of the tested compounds between 400 nm and 800 nm were preliminary recorded and indicated that the compounds had no absorption peaks near the wavelengths where the assays were performed. All the assays were performed in triplicate, and the results are presented as the averages.

#### 4.2.1. Antiradical Assays

##### ABTS^+^ Radical Scavenging Assay

The green ABTS**˙**^+^ (2,2′-azinobis-(3-ethylbenzothiazoline-6-sulfonic acid) decolorization assay to ABTS, as reported by Re et al., was performed according to our group report [21,26]. The stability of the reagent was verified at λ = 734 nm for one hour to ensure the constant absorption (approximately equal to 0.7) after preparation in potassium phosphate buffer (0.1 M, pH = 7.4) and activation of the reagent using MnO_2_ [21]. 

Amounts of 10 µL, 15 µL, 20 µL, 30 µL, 40 µL and 50 µL from stock solutions of the compounds; Trolox and ascorbic acid and the appropriate amount of DMSO were added to all the cuvettes, until a total volume of 50 µL was reached. Later, 2950 µL of ABTS**˙**^+^ reagent were added to all cuvettes, and the resultant mixtures were shaken well for over 10 min at room temperature in the dark. The absorbance of the resultant solutions was determined spectrophotometrically at λ = 734 nm against a blank sample. The activity of the tested compounds was assessed using Equation (1):

(1) $${\mathrm{ABTS}}^{+}\;\mathrm{scavenging}\;\left(\%\right)=\frac{\mathrm{control}\;\mathrm{absorbance}-\mathrm{sample}\;\mathrm{absorbance}}{\mathrm{control}\;\mathrm{absorbance}}\times 100$$

##### DPPH Radical Scavenging Assay

The DPPH**˙** radical scavenging assay was previously reported by Brand-Williams et al. [27,28]. It is based on the transfer of one hydrogen from the analyzed substrate to the violet stable free radical of DPPH**˙** (2,2-diphenyl-1-picrylhydrazyl), converting it to a yellow compound. The change in absorbance of the reagent was proportional to the amount of DPPH**˙** neutralized [20].

The preparation of the working solution of DPPH was previously reported and performed by dissolving the solid reagent in methanol. Supplementary amounts of solvent were added until a constant absorbance of the reagent approximately equal to 1 was achieved at λ = 517 nm against a blank sample [21,23]. 

In each cuvette were added 10 µL, 15 µL, 20 µL, 30 µL, 40 µL, 50 µL and 60 µL stock solutions of the compounds and the reference compounds and the appropriate amount of DMSO, until a total volume of 100 µL was reached. The reference compounds were prepared in the same manner. After mixing well, 3900 µL of DPPH**˙** solution were added to all cuvettes, and the mixtures were shaken well for over 10 min in the dark at room temperature. The absorbance of the samples was measured at λ = 517 nm against a blank sample. The percent of DPPH**˙** radical scavenging activity of the tested compounds was assessed using the following equation:

(2) $$\mathrm{DPPH}\;\mathrm{scavenging}\;\left(\%\right)=\frac{\mathrm{control}\;\mathrm{absorbance}-\mathrm{sample}\;\mathrm{absorbance}}{\mathrm{control}\;\mathrm{absorbance}}\times 100$$

##### NO Radical Scavenging Assay

Based on some adaptations of the protocols reported in the literature, scavenging of the NO**˙** radical was performed using nitroprusside decomposition at the physiological pH and colorimetric quantification of the resultant azo dye after performing a Griess reaction [24,25,26].

Briefly, to a mixture of 200 µL of 10 mM sodium nitroprusside, 100 µL of test solution (1 mg/mL) and 400 µL of phosphate buffer (PBS, pH = 7.4), 1 mL of sulfanilic acid 0.33% in 20% acetic acid was added 150 min later. After 5 min, 1 mL of naphthylethylenediamine dichloride 0.1% was added. After another 30 min, the absorbance of the samples was measured at 546 nm against a blank sample. The percent of NO**˙** radical scavenging activity of the tested compounds was assessed using the following equation:

(3) $$\mathrm{NO}\;\mathrm{scavenging}\;\left(\%\right)=\frac{\mathrm{control}\;\mathrm{absorbance}-\mathrm{sample}\;\mathrm{absorbance}}{\mathrm{control}\;\mathrm{absorbance}}\times 100$$

#### 4.2.2. Electron Transfer Assays

##### Ferric Reducing Antioxidant Potential (FRAP) 

Using the FRAP test, the reducing antioxidant potential of the analyzed compounds could be determined according to the modified method originally proposed by Benzie and Strain [21,28,29]. One thousand microliters of FRAP reagent and fifty microliters of solution (1 mg/mL) of the tested compounds and standards were mixed in glass test tubes. For 30 min, the resulting mixtures were stirred vigorously in a rotating shaker (GFL Gesellschaft für Labortechnik, Burgwedel, Germany). In all test tubes, 2000 μL of acetate buffer 0.3 M (pH = 3.6) were added. The absorbance of the solutions was measured against a blank sample prepared from 50 μL of DMSO and 1000 μL of FRAP reagent at λ = 593 nm. The reducing antioxidant potential of the compounds was expressed as a percentage of the activity of the reference compounds, based on Equation (4):

(4) $$\%\;\mathrm{of}\;\mathrm{control}\;\mathrm{activity}=\frac{\mathrm{sample}\;\mathrm{absorbance}}{\mathrm{reference}\;\mathrm{absorbance}}\times 100$$

##### Phosphomolybdate Assay for Total Antioxidant Capacity (TAC) 

The TAC assay of the tested compounds was determined by a method previously reported in the literature [21,30,31,32]. One thousand microliters of the reagent [16] and one hundred microliters of solution of the compounds and standards (1 mg/mL) were mixed well in glass test tubes and incubated in a water bath at 95 °C for 90 min. The solutions were left at room temperature, and after cooling, 2000 μL of water were added to all the tubes. The absorbance of the solutions was measured at λ = 695 nm against a blank sample. The TAC of the compounds was calculated using Equation (4).

##### Reducing Power (RP) Assay

In this assay the tested compounds reduced ferric ions from potassium ferricyanide, and with ferric ions, the resultant ferrocyanide gave a blue complex. The followed protocol was previously reported [21,22,32]. In glass test tubes, 100 µL of the compounds and standards solutions (1 mg/mL) were mixed with 1000 µL of DMSO, 400 µL of phosphate buffer (0.2 M, pH = 6.6) and 400 µL of K_3_[Fe(CN)_6_] solution (1% *w*/*v*). The mixtures were incubated for 20 min in bath water at 50 °C. After cooling at room temperature, 500 µL of trichloroacetic acid (10% *w*/*w*) were added in all test tubes, and the resultant mixtures were left to stand for 30 min at room temperature. Two hundred and fifty microliters of the solution were collected carefully and mixed with one hundred and forty microliters of FeCl_3_ solution (0.1% *w*/*v*) and one thousand microliters of distilled water. The absorbance was measured against a blank sample at λ = 700 nm. The reducing power of the tested compounds was expressed as a percent of the reference compound activity, based on Equation (4).

##### Cupric Reducing Antioxidant Capacity (CUPRAC) Assay

In this assay, the capacity of the compounds to transfer electrons to cupric ions in order to reduce them to cuprous ions was evaluated. The cuprous ions that resulted were chelated by the chromogenic neocuproine, giving an orange complex. The followed protocol represents an adaptation of previous similar reports [33,34]. One milliliter of CuCl_2_ ten millimeters, one millimeter of ammonium acetate buffer one millimeter and one millimeter of seven point five millimeters neocuproine ethanolic solution were mixed well in test tubes with one hundred microliters of the samples and reference compound solutions. For the next 30 min, the test tubes containing the resultant mixtures were shaken vigorously on a rotating shaker in the dark. Five hundred microliters from the solutions were taken and mixed with one thousand microliters of distilled water. The absorbance was measured at λ = 450 nm against a blank sample. The cupric ion reduction of the tested compounds was expressed as a percent of the reference compound activity, based on Equation (4).

#### 4.2.3. Transition Metal Ions Chelation Assays

##### Fe^2+^ Chelation Assay

The protocol used to assess the compounds’ chelating capacity of Fe^2+^ was adapted from the initial report of Benzie and Strain [29,35,36]. The principle behind this assay is the disk formation of a colored complex between ferrozine and Fe^2+^ ions that were not chelated by the tested compounds. In the present assay, new synthesized molecules were added in various concentrations to evaluate, in a better way, their chelation capacity. In the test tubes, increasing amounts (25 µL, 35 µL, 50 µL, 75 µL, 100 µL, 150 µL, 200 µL, 300 µL and 400 µL) of the tested compounds and Na_2_-EDTA solutions (1 mg/mL in DMSO) were added. Then, DMSO was added, until a total volume of 500 µL was found in all the test tubes. 500 µL of DMSO was used as the control. Later, 1000 µL of 0.125 mM FeSO_4_ were added in all test tubes. After 15 min of shaking, 500 µL of 0.3125 mM ferrozine were added, and the shaking continued for another 15 min. The absorbance of the solutions was determined at λ = 562 nm against a blank sample. 

The results were calculated using the following equation:

(5) $$\mathrm{iron}\;\mathrm{chelation}\;\left(\%\right)=\frac{\mathrm{control}\;\mathrm{absorbance}-\mathrm{sample}\;\mathrm{absorbance}}{\mathrm{control}\;\mathrm{absorbance}}\times 100$$

##### Cu^2+^ Chelation Assay

The protocol used to evaluate the Cu^2+^ chelating activity of the compounds and EDTA disodium salt was determined using a slightly modified version of the method presented by Wu et al. [37,38]. 100 µL, 250 µL and 500 µL of each compound were taken from the stock solutions (1 mg/mL in DMSO), and DMSO was added to each test tube, until a 500 µL total volume was reached. 400 µL of 3 mM CuSO_4_ solution in hexamine buffer (10 mM hexamine and 10 mM KCl) was added. After shaking well over 5 min, 75 µL of murexide 1 mM and 2 mL of water were added. The absorbance was measured at 485 nm and 520 nm after 3 min of incubation at room temperature. The two wavelengths corresponded to the absorbance of the murexide–copper (II) complex and the free murexide, respectively. The ratio of the two absorbances was proportional to the free copper (II) in the solution. The copper chelation capacity of the compounds was calculated using the following equation:

(6) $$\mathrm{copper}\;\mathrm{chelation}\;\left(\%\right)=\frac{{\left(\frac{A_{485}}{A_{520}}\right)}_{\mathrm{control}}-{\left(\frac{A_{485}}{A_{520}}\right)}_{\mathrm{sample}}}{{\left(\frac{A_{485}}{A_{520}}\right)}_{\mathrm{control}}}\times 100$$

### 4.3. Theoretical Quantum and Thermodynamical Calculations

Due to the polyphenolic structure of compounds **5a**–**l**, according to the literature, some quantum and thermodynamic parameters can describe the intensity of their structure-related activity. The HOMO and LUMO frontier orbital energy levels and the energy gap between the two are the most commonly used theoretical descriptors. The area of the molecule in which HOMO is considered represents the site responsible for neutralizing the radicals. The higher the energy value of the HOMO, the more easily the molecule will release electrons and, indirectly, hydrogen atoms. The HOMO–LUMO gap is an important derived descriptor as well. The LUMO describes the ability of a molecule to accept an electron, and in this context, the lowest possible energy level in this orbital is wanted. Therefore, a lower HOMO–LUMO gap indicates that the molecule has a higher antiradical effect [12,14,39].

From a thermodynamic point of view, it is desirable for a molecule that, after the release of a hydrogen atom, the resulting unpaired electron radical is stabilized by internal conjugation as well as possible. Thus, the smaller the enthalpy of the radical resulting after hydrogen atom release, the more it proves the susceptibility of certain molecular sites for hydrogen atom release. In the present research, we evaluated the susceptibility of some molecular sites to yield hydrogen atoms: phenolic groups, hydrazone groups, azomethine groups and the allylic chain of compounds **5a, 5b** and **5c**, the results being expressed as BDE (bond dissociation enthalpy). Obviously, in this determination, no BDEs were calculated for other sites that can only release hydrogen atoms under extremely energetic conditions, situations unsuitable for biological systems—for example, carbon–hydrogen bonds on aromatic nuclei or aliphatic chains.

All the theoretical studies presented in this subsection were performed using the previously reported protocols [21,40]. The images representing the lowest conformation of the compounds were generated using Chimera 1.10.2 [41].

## 5. Conclusions

This study illustrates the synthesis, characterization and preliminary in vitro antioxidant activity evaluation of some new 4(3H)-quinazolinone derivatives. The evaluation of the antioxidant activity was performed in vitro by applying various antiradical and electron transfer-based assays. 

Similar results were obtained in the antiradical assays (ABTS^+^, DPPH and NO), in which the phenolic derivatives 4(3H)-quinazolinone compounds **5h**, **5j** and **5k** had a higher radical scavenging activity than those of the reference antioxidants used.

Phenolic derivatives **5a**, **5b**, **5d**, **5e**, **5g**, **5h**, **5j** and **5k** were the most active compounds of the newly synthesized series, following the evaluation of the antioxidant activity (TAC and CUPRAC). The antioxidant potential was better than that of ascorbic acid and Trolox, used as reference antioxidants in the in vitro electron transfer assays.

In the in vitro transition metal ions chelation assays (Fe^2+^ and Cu^2+^), the best chelating properties were shown by the 2,3-disubstituted catechol derivatives **5d** and **5j**, even if their activity was inferior to that of Na_2_-EDTA. 

Analyzing the HOMO–LUMO gap, the lowest values were found in the case of catechol derivatives—more precisely, in the case of the series of 2,3-disubstituted compounds (**5a**, **5d**, **5g** and **5j**). The values of these derivatives were lower than the series of 3,4-disubstituted compounds, ranging from 3.92 eV for compound **5j** to 3.78 eV for compound **5d**.

Thus, the variety and complexity of the assays can outline an interesting antioxidant profile for the 12 synthetic quinazolin-4(3H)-one compounds. Among the four series of analyzed derivatives, the compounds that have a hydroxyl group in position 2 on phenyl have a stronger antioxidant action. 

The results of the in vitro studies performed to date on these compounds and reported in this paper are encouraging in terms of antioxidant activity. This limitation of the study without the use of experimental models on cells or animals was necessary in order to be able to determine which compounds are of interest to be selected for further evaluation. Therefore, the present research is the basis for future research to evaluate the antioxidant activity of these compounds in experimental cell models and should then be validated in vivo in animal experimental models for the compounds that show good activity and a lack of in vitro cellular toxicity. 

## Figures and Tables

**Table 1:** Results of the ABTS^+^ scavenging assay.

Compound	% of ABTS^+^ Scavenging						IC_50_ (µg/mL)
	3.33 µg/mL	4.99 µg/mL	6.66 µg/mL	9.99 µg/mL	13.32 µg/mL	16.65 µg/mL	
**5a**	14.13	21.94	30.87	44.61	60.39	75.09	11.09
**5b**	52.73	60.97	71.00	88.62	+	+	2.87
**5c**	32.20	59.31	88.59	+	+	+	4.40
**5d**	49.82	56.51	63.76	76.99	86.60	+	3.17
**5e**	28.63	38.66	49.82	65.81	82.66	+	7.07
**5f**	44.01	55.27	66.73	83.76	+	+	4.15
**5g**	42.49	66.39	92.31	+	+	+	3.85
**5h**	60.48	72.18	80.92	+	+	+	1.54
**5i**	33.09	45.73	59.07	84.39	+	+	5.52
**5j**	27.51	42.01	53.16	70.44	90.53	+	6.55
**5k**	60.34	72.03	83.81	+	+	+	1.86
**5l**	57.62	66.81	74.19	91.08	+	+	1.73
**Ascorbic acid**	60.97	73.24	87.73	+	+	+	2.01
**Trolox**	38.66	53.16	66.54	94.57	+	+	4.66

+ Represents more than 95%.

**Table 2:** Results of the DPPH scavenging assay.

Compound	% of DPPH Scavenging							IC_50_ (µg/mL)
	2.5 µg/mL	3.75 µg/mL	5 µg/mL	7.5 µg/mL	10 µg/mL	12.5 µg/mL	15 µg/mL	
**5a**	−	−	−	−	6.16	19.89	28.55	>15
**5b**	49.14	58.34	71.82	90.78	+	+	+	2.30
**5c**	10.73	19.89	28.14	41.61	56.11	69.67	83.89	9.02
**5d**	−	−	−	−	8.45	23.32	33.12	>15
**5e**	5.58	20.03	30.70	50.23	75.09	94.62	+	7.32
**5f**	27.37	38.48	46.70	61.71	80.84	+	+	5.60
**5g**	−	−	−	−	−	6.16	17.60	>15
**5h**	49.65	58.40	64.90	75.62	+	+	+	2.30
**5i**	47.36	55.37	58.80	69.95	81.03	+	+	2.88
**5j**	−	−	−	−	−	6.04	18.75	>15
**5k**	49.65	60.36	71.55	90.16	+	+	+	2.47
**5l**	22.18	32.54	46.42	64.28	81.69	+	+	5.82
**Ascorbic acid**	47.45	55.71	64.21	79.16	94.39	+	+	2.83
**Trolox**	28.53	40.42	53.87	75.85	94.85	+	+	4.68

− Represents less than 5%; + represents more than 95%.

**Table 3:** Results of the NO scavenging assay.

Compound	% of NO Scavenged
**5a**	37.84
**5b**	42.60
**5c**	18.72
**5d**	46.79
**5e**	44.12
**5f**	30.72
**5g**	43.47
**5h**	50.53
**5i**	32.14
**5j**	50.28
**5k**	42.70
**5l**	23.09
**Gentisic acid**	48.14

**Table 4:** Results of the Ferric Reducing Antioxidant Potential (FRAP), Total Antioxidant Capacity (TAC), Reducing Power (RP) and Cupric Reducing Antioxidant Capacity (CUPRAC) assays, expressed as the % activity of ascorbic acid and Trolox activity.

Compound	% of Activity of Ascorbic Acid				% of Activity of Trolox			
	FRAP	TAC	RP	CUPRAC	FRAP	TAC	RP	CUPRAC
**5a**	25.59	58.40	38.94	46.72	29.43	112.81	56.74	44.53
**5b**	71.09	37.66	60.11	155.11	81.75	72.75	87.58	147.84
**5c**	16.18	46.38	32.82	6.96	18.60	89.59	47.82	6.63
**5d**	19.76	65.42	36.71	91.61	22.73	126.38	53.49	87.32
**5e**	24.83	20.91	51.08	182.84	28.56	40.39	74.43	174.28
**5f**	8.38	51.91	15.07	9.74	9.63	100.28	21.97	9.28
**5g**	51.17	89.39	67.74	56.00	58.85	172.68	98.70	53.37
**5h**	71.99	33.71	71.76	99.24	82.78	65.12	104.56	94.59
**5i**	14.08	39.31	39.23	17.94	16.19	75.94	57.17	17.10
**5j**	26.64	72.86	30.16	45.95	30.64	140.75	43.94	43.80
**5k**	63.38	48.33	67.42	165.05	72.89	93.36	98.24	157.31
**5l**	10.80	55.33	18.66	32.59	12.42	106.88	27.19	31.07

**Table 5:** Results of the ferrous ions chelation capacity evaluation.

Compound	Chelation Capacity (%)								
	17.70 µg/mL	20.59 µg/mL	29.41 µg/mL	44.11 µg/mL	58.82 µg/mL	88.23 µg/mL	117.64 µg/mL	257.46 µg/mL	343.28 µg/mL
**5a**	−	−	−	−	−	−	−	−	15.85
**5b**	−	−	−	−	−	−	−	−	−
**5c**	−	−	−	−	−	−	−	−	−
**5d**	−	−	−	−	9.01	15.69	24.50	36.86	55.57
**5e**	−	−	−	−	−	−	−	−	−
**5f**	−	−	−	−	−	−	−	−	−
**5g**	−	−	−	−	−	−	−	−	6.45
**5h**	−	−	−	−	−	−	−	−	−
**5i**	−	−	−	−	−	−	−	−	−
**5j**	−	−	10.98	15.85	19.47	27.02	34.29	47.28	57.41
**5k**	−	−	−	−	−	−	−	−	−
**5l**	−	−	−	−	−	−	−	−	−
**EDTA-Na_2_**	1.32	20.59	42.89	95.10	+	+	+	+	+

− Represents less than 5%; + represents more than 95%.

**Table 6:** Results of the cupric ion chelating capacity evaluation.

Compound	Chelation Capacity (%)		
	3.36 µg/mL	8.40 µg/mL	16.80 µg/mL
**5a**	5.90	12.00	21.80
**5b**	5.87	11.11	18.01
**5c**	7.60	15.24	33.04
**5d**	5.39	14.99	31.86
**5e**	4.21	9.12	16.01
**5f**	5.68	13.68	23.71
**5g**	2.26	14.63	33.32
**5h**	0.49	5.90	13.56
**5i**	5.20	13.99	23.89
**5j**	2.02	8.60	18.86
**5k**	4.11	8.74	14.86
**5l**	4.50	8.73	15.72
**EDTA-Na_2_**	10.39	22.68	44.51

**Table 7:** The energy of the frontier orbitals and the BDE for the H_1_–H_5_ sites in the studied compounds, according to the numeration presented in Figure 3.

Compound	Frontier Orbitals (eV)			X-H BDE (kcal/mol)				
	HOMO	LUMO	Gap	H_1_	H_2_	H_3_	H_4_	H_5_
**5a**	−5.54	−1.66	3.88	81.54	80.20	N/A	89.57	101.16
**5b**	−5.45	−1.53	3.92	N/A	77.50	72.19	97.22	99.87
**5c**	−5.69	−1.42	4.27	88.04	N/A	77.00	81.23	100.36
**5d**	−5.45	−1.67	3.78	81.60	80.23	N/A	88.08	101.25
**5e**	−5.45	−1.55	3.90	N/A	77.44	72.00	88.82	99.78
**5f**	−5.50	−1.62	3.88	85.56	N/A	85.25	86.38	104.97
**5g**	−5.54	−1.67	3.87	81.49	80.17	N/A	88.15	101.18
**5h**	−5.45	−1.50	3.95	N/A	77.45	72.17	90.60	99.80
**5i**	−5.68	−1.36	4.32	80.25	N/A	76.86	81.08	102.56
**5j**	−5.53	−1.61	3.92	81.56	80.14	N/A	87.98	101.20
**5k**	−5.43	−1.49	3.94	N/A	77.40	72.07	88.96	99.84
**5l**	−5.58	−1.28	4.30	82.70	N/A	79.22	82.64	105.04

**Table 8:** The conformation of the lowest energy of the compounds with the depiction of the resulting hydrogen bonds.

Compound	Conformation	Compound	Conformation
**5a**	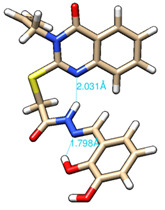	**5g**	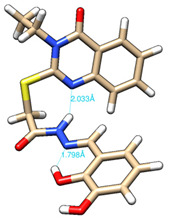
**5b**	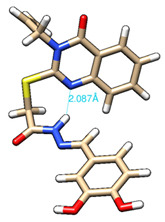	**5h**	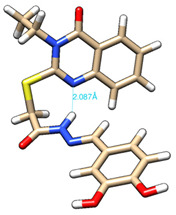
**5c**	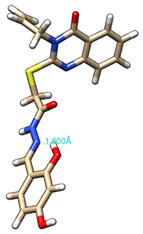	**5i**	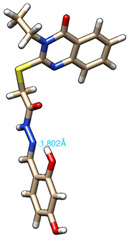
**5d**	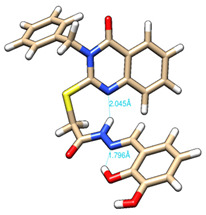	**5j**	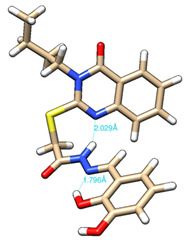
**5e**	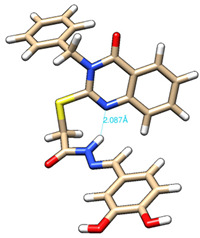	**5k**	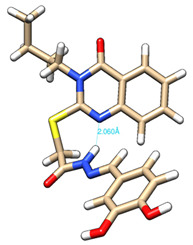
**5f**	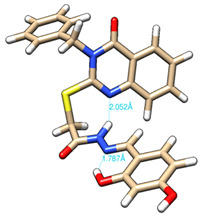	**5l**	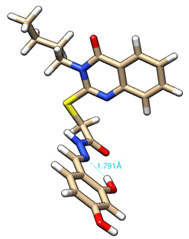

## Data Availability

Not applicable.

## Supplementary Materials

The following supporting information can be downloaded at https://www.mdpi.com/article/10.3390/molecules27082599/s1: Figures S1–S24: The IR spectrums for compounds **1a**–**5l**. Figures S25–S48: The MS spectrums for compounds **1a**–**5l**. Figures S49–S72: The ^1^H-NMR spectrum for compounds **1a**–**5l**. Figures S73–S96: The ^13^C-NMR spectrum for compounds **1a**–**5l**. Table S1: Spin density map dependent on the phenolic radical for compounds **5a**–**l**.

## Sample Availability

Samples of all compounds are available from the authors.
