# Supplementary material for: Synthesis of New Phenolic Derivatives of Quinazolin-4(3H)-One as Potential Antioxidant Agents—In Vitro Evaluation and Quantum Studies
Source: Molecules. 2022 Apr 18;27(8):2599. doi: 10.3390/molecules27082599 (PMC9028568; doi:10.3390/molecules27082599)
Supplement: Supplementary file 1 [file molecules-27-02599-s001.zip › molecules-1668344-supplementary.pdf]

molecules

Article
Supplementary Material

1. Figures

1.1. The IR spectra

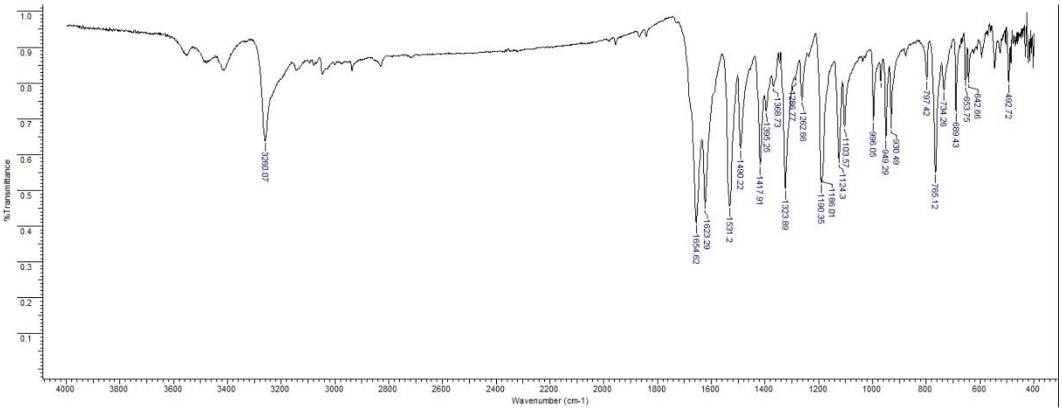

Figure S1. The IR spectrum for the compound **1a.**

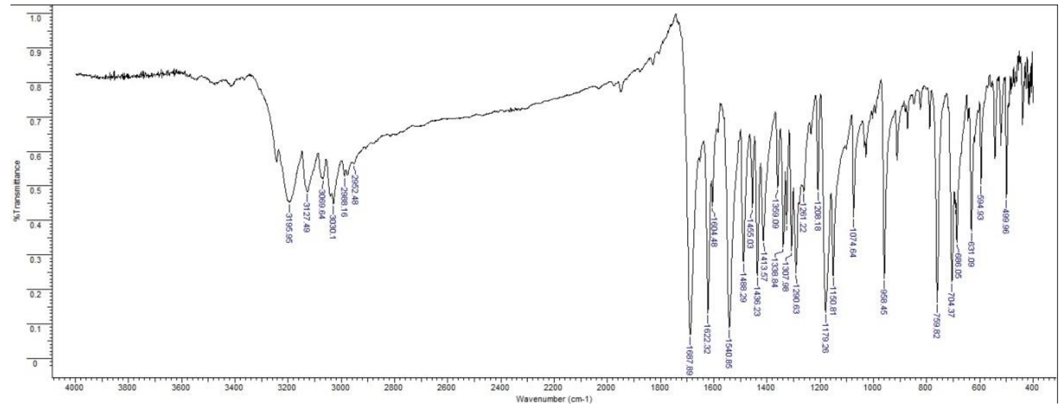

Figure S2. The IR spectrum for the compound **1b.**

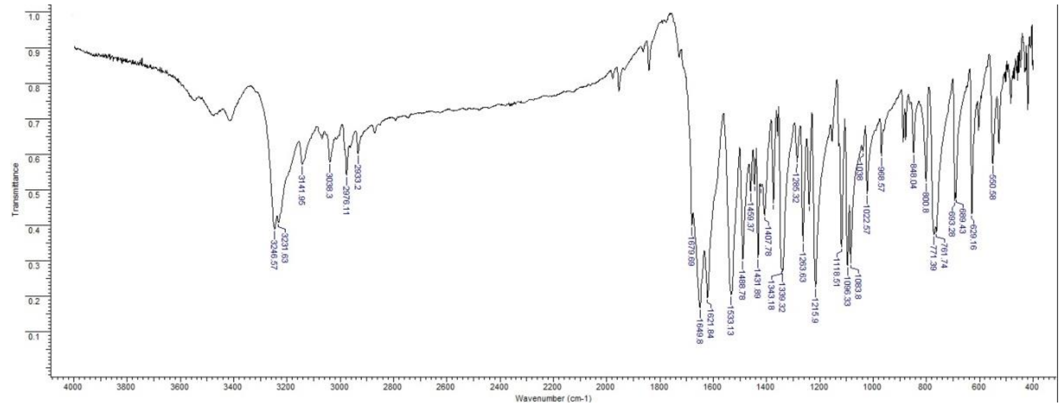

Figure S3. The IR spectrum for the compound **1c.**

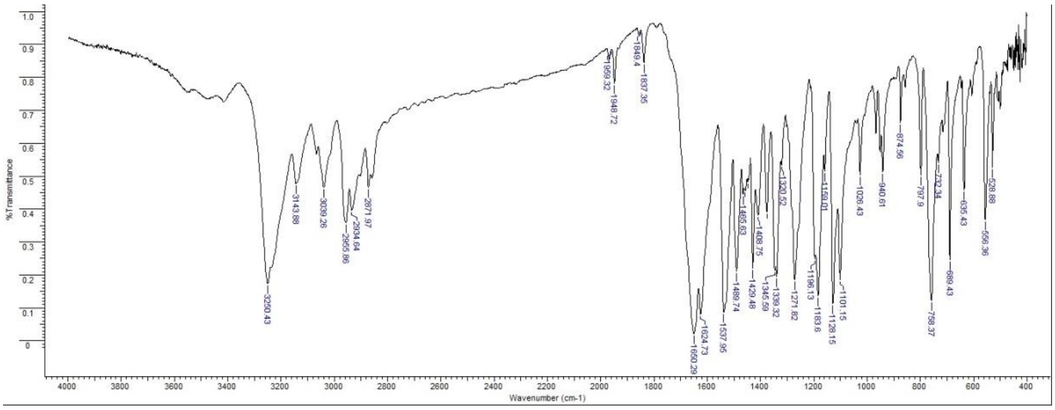

Figure S4. The IR spectrum for the compound **1d.**

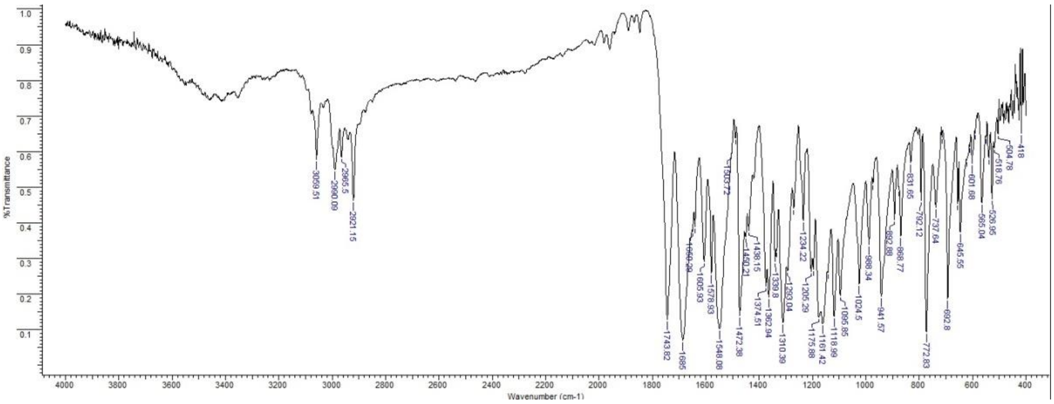

Figure S5. The IR spectrum for the compound **2a.**

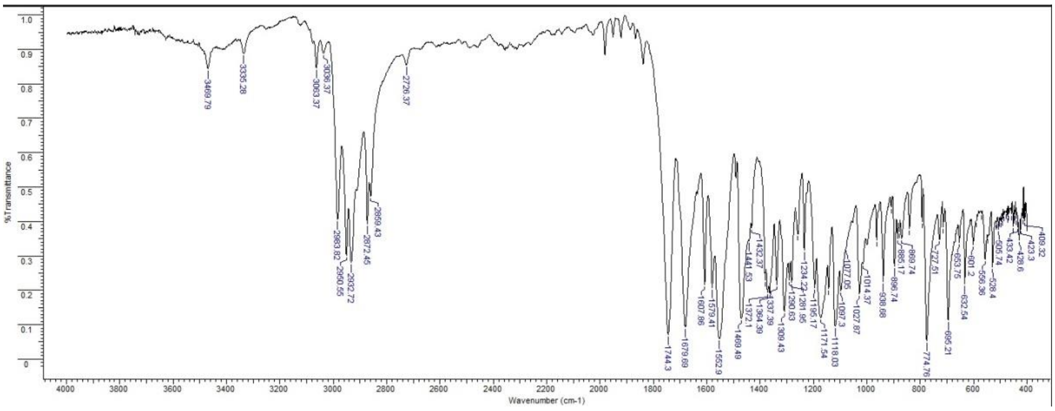

Figure S6. The IR spectrum for the compound **2b.**

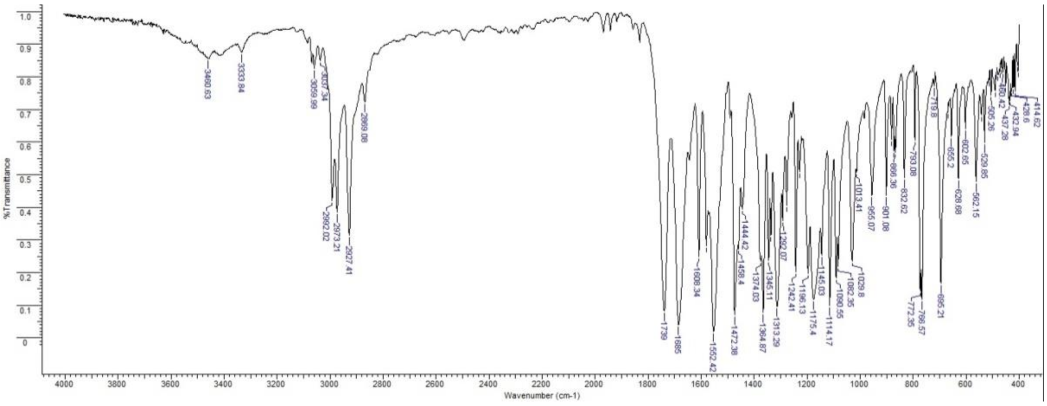

Figure S7. The IR spectrum for the compound **2c.**

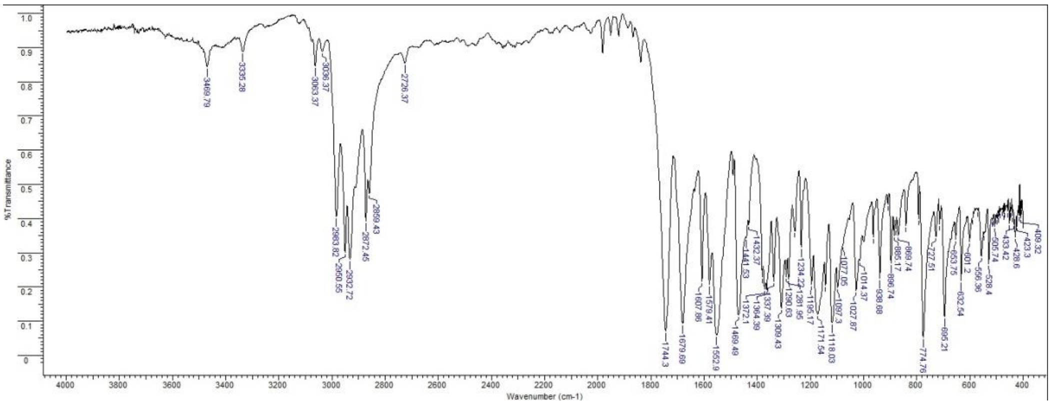

Figure S8. The IR spectrum for the compound **2d.**

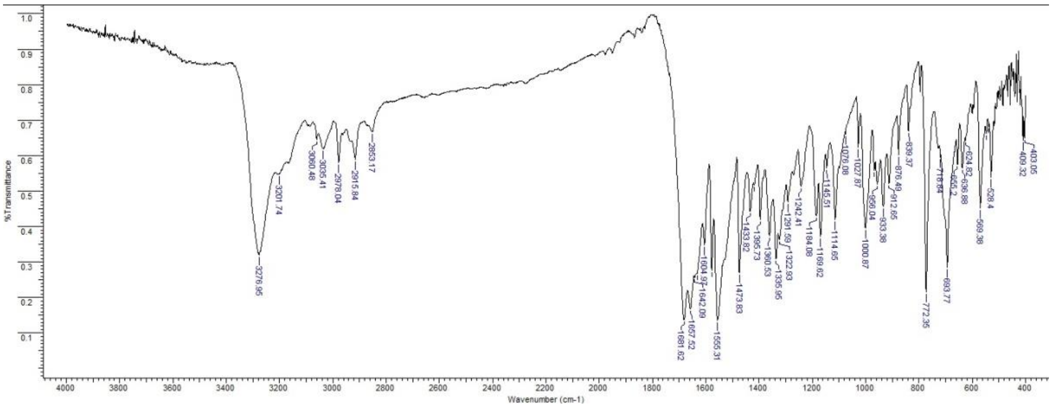

Figure S9. The IR spectrum for the compound **3a.**

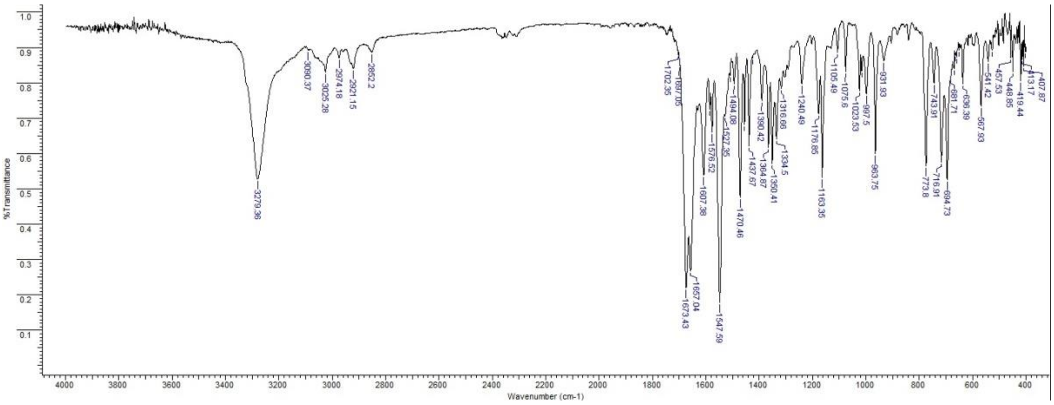

Figure S10. The IR spectrum for the compound **3b.**

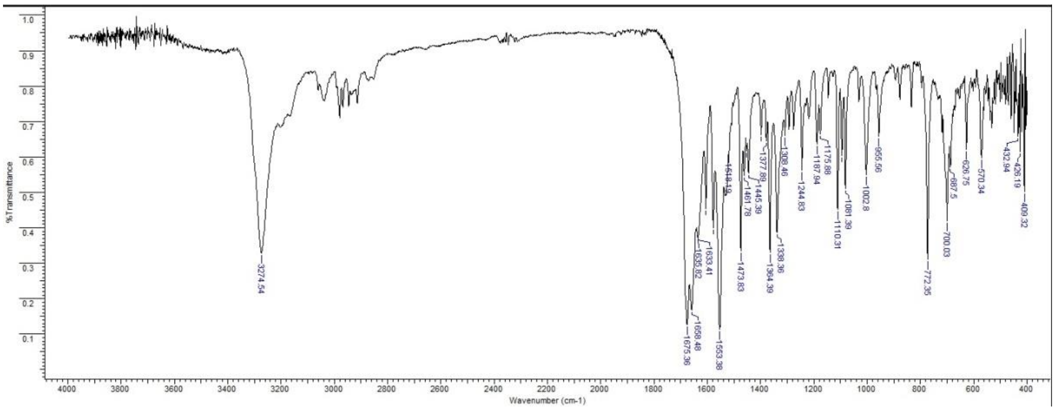

Figure S11. The IR spectrum for the compound **3c.**

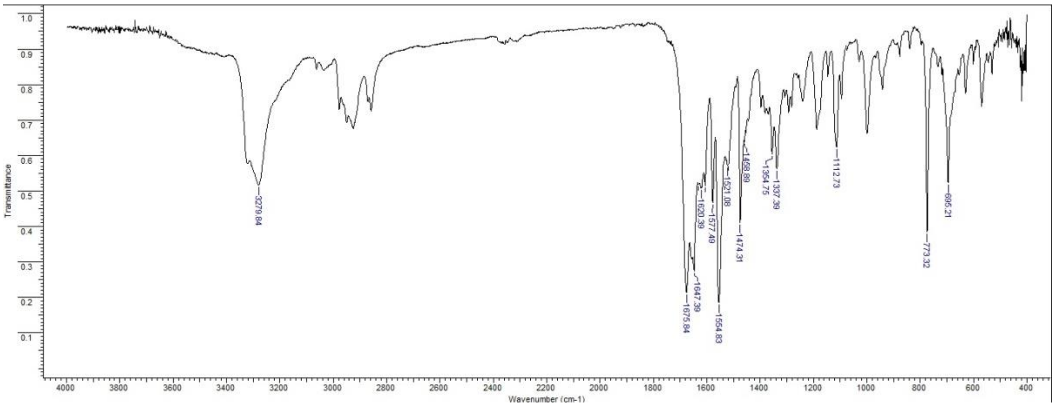

Figure S12. The IR spectrum for the compound **3d.**

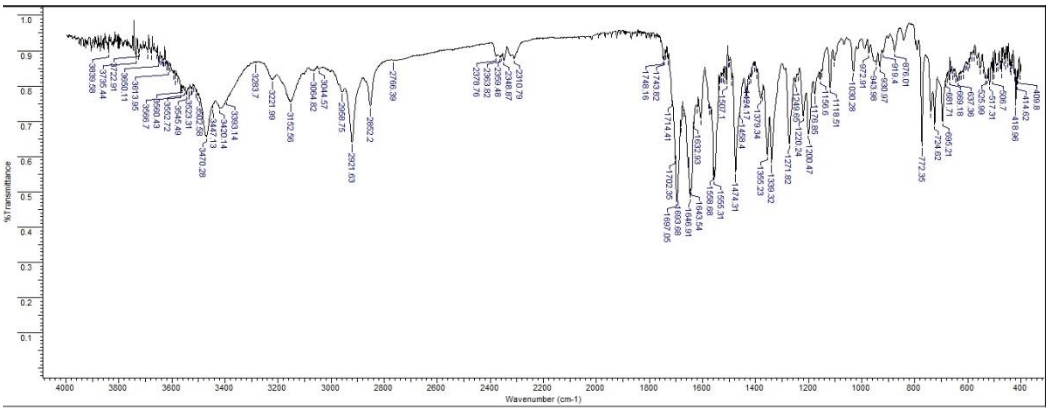

Figure S13. The IR spectrum for the compound **5a.**

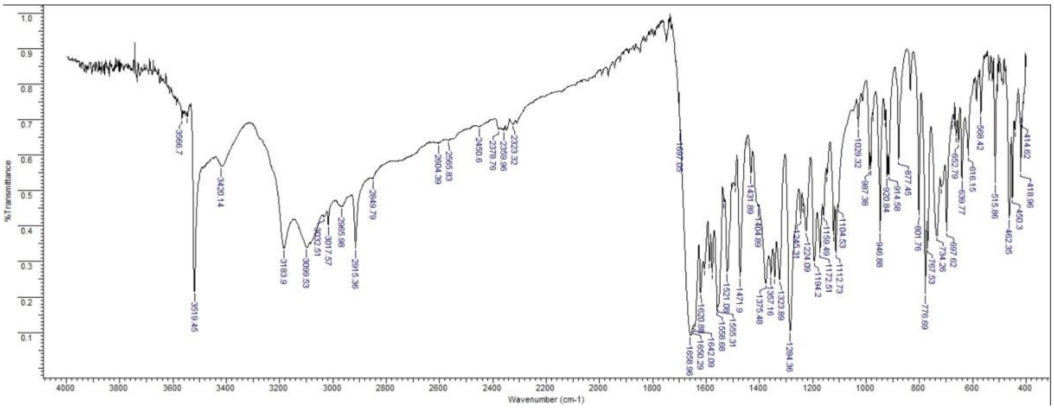

Figure S14. The IR spectrum for the compound **5b.**

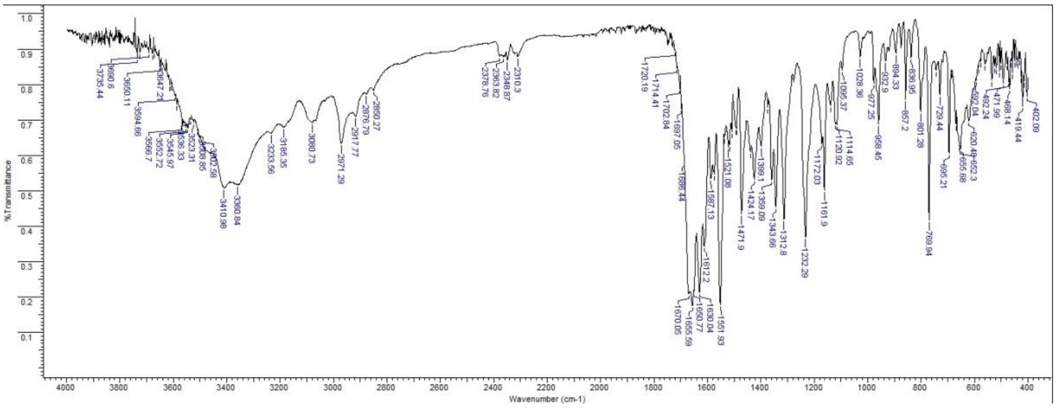

Figure S15. The IR spectrum for the compound **5c.**

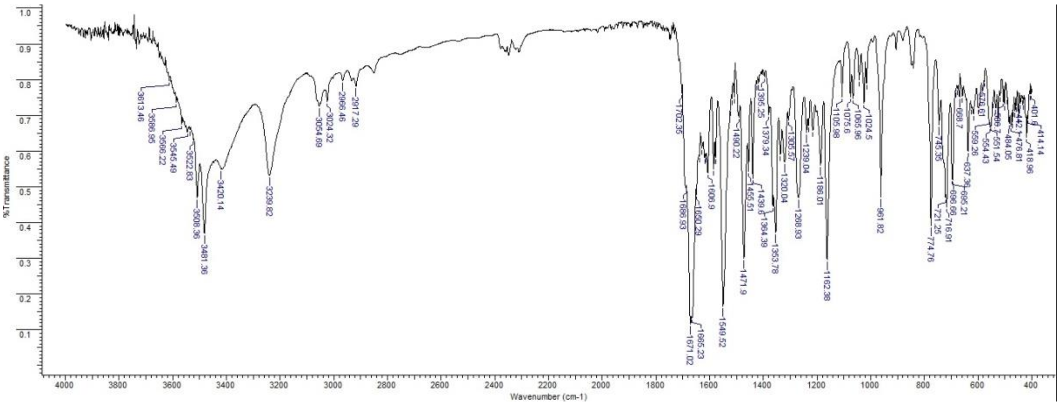

Figure S16. The IR spectrum for the compound **5d.**

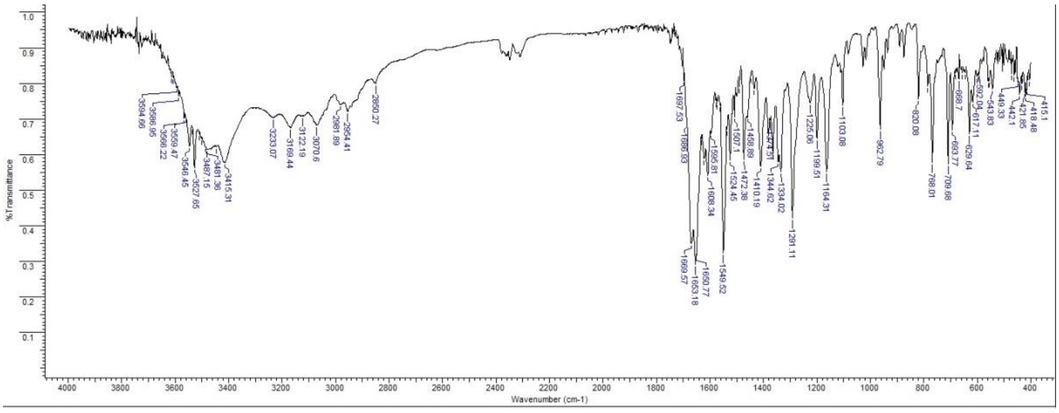

Figure S17. The IR spectrum for the compound **5e.**

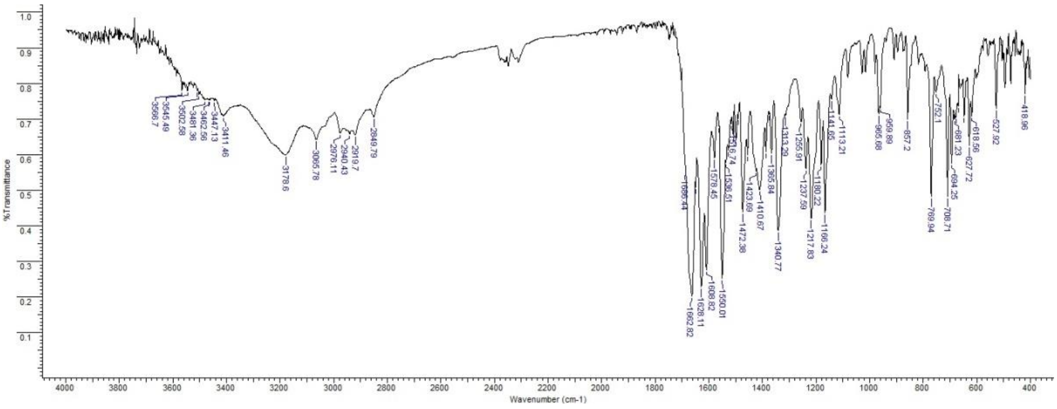

Figure S18. The IR spectrum for the compound **5f.**

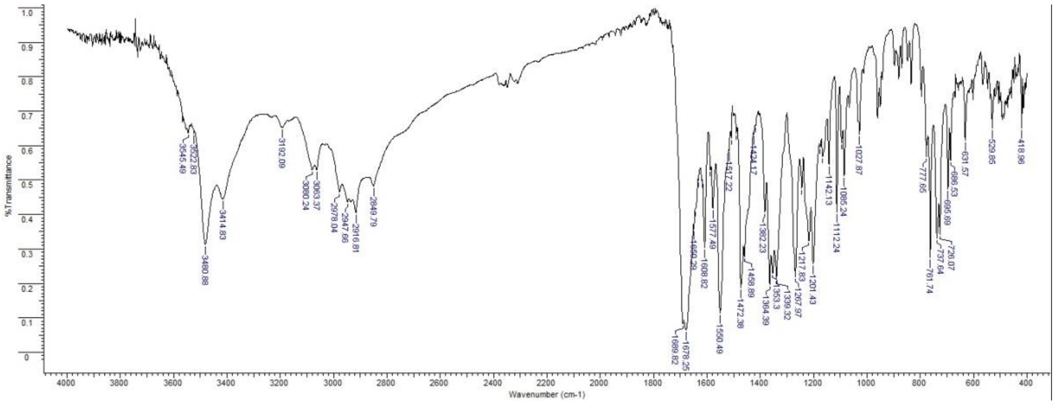

Figure S19. The IR spectrum for the compound **5g.**

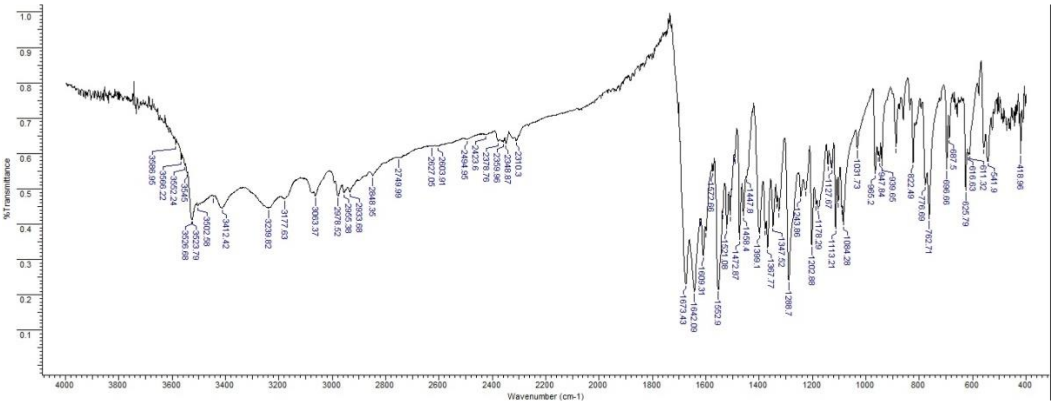

Figure S20. The IR spectrum for the compound **5h.**

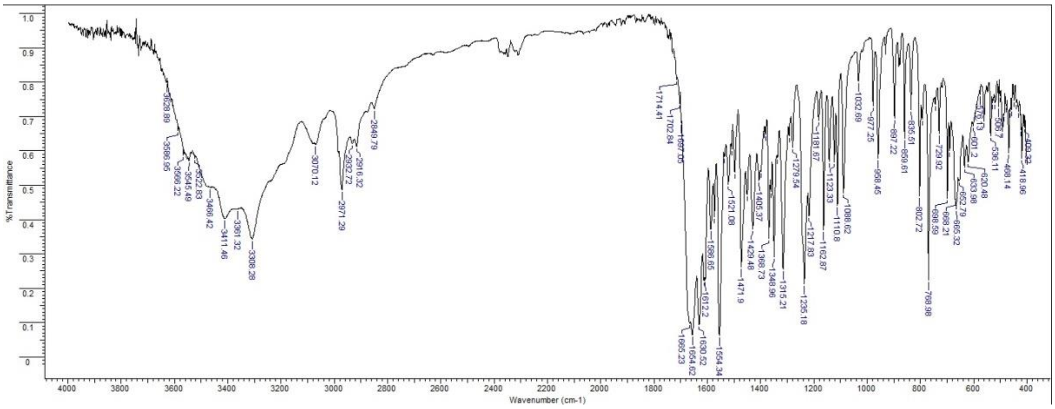

Figure S21. The IR spectrum for the compound **5i.**

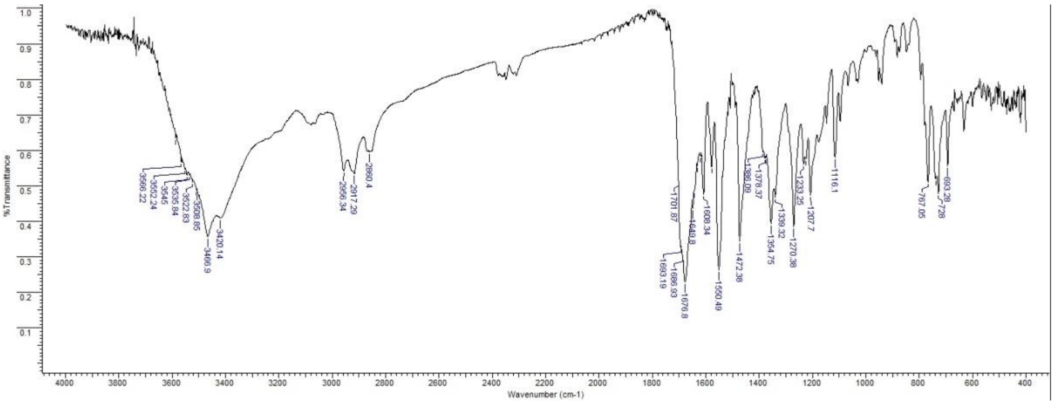

Figure S22. The IR spectrum for the compound **5j.**

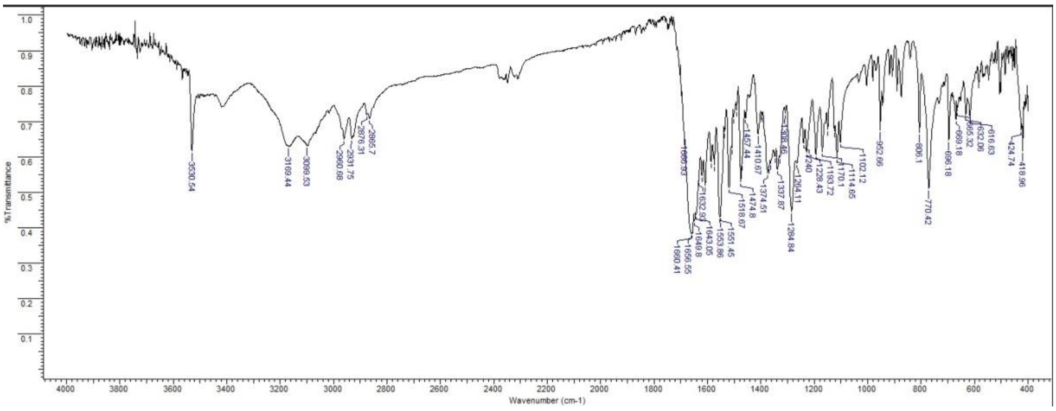

Figure S23. The IR spectrum for the compound **5k.**

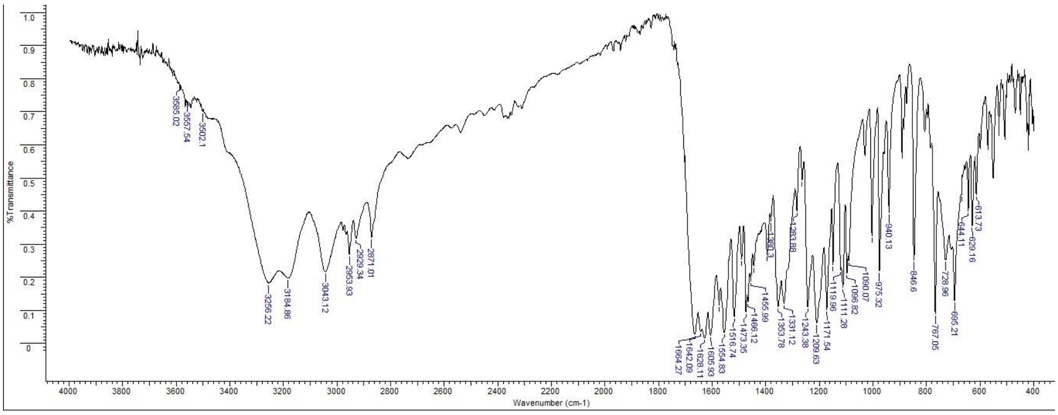

Figure S24. The IR spectrum for the compound **5l.**

1.2. The MS spectra

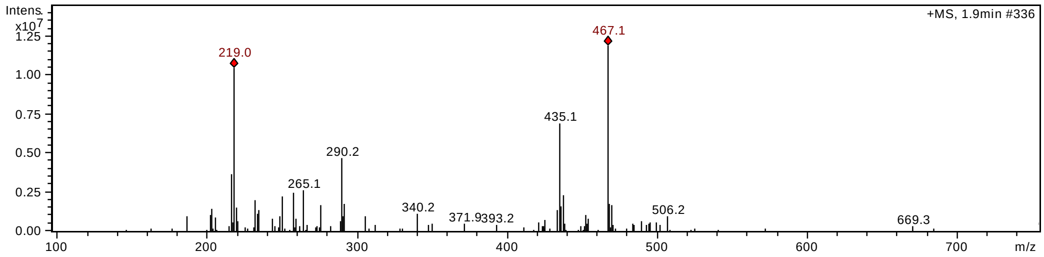

Figure S25. The MS spectrum for the compound **1a.**

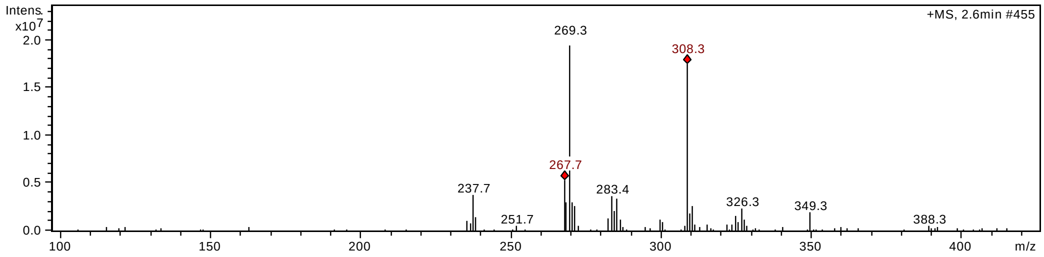

Figure S26. The MS spectrum for the compound **1b.**

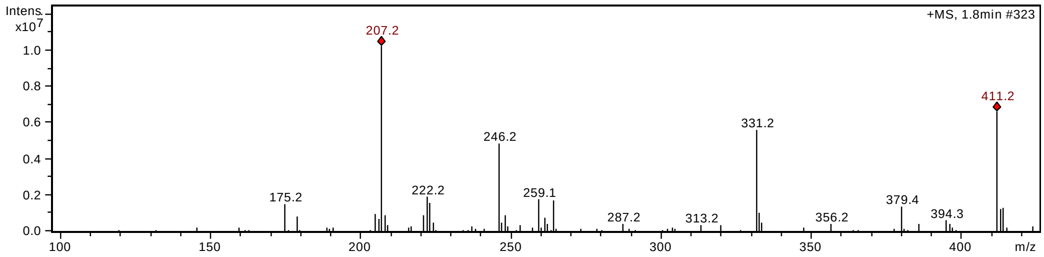

Figure S27. The MS spectrum for the compound **1c.**

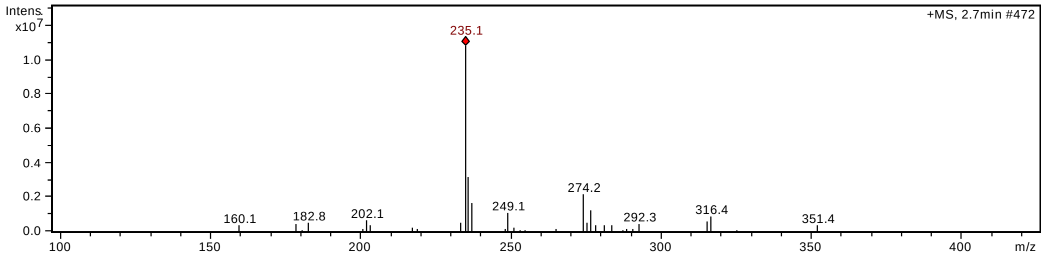

Figure S28. The MS spectrum for the compound **1d.**

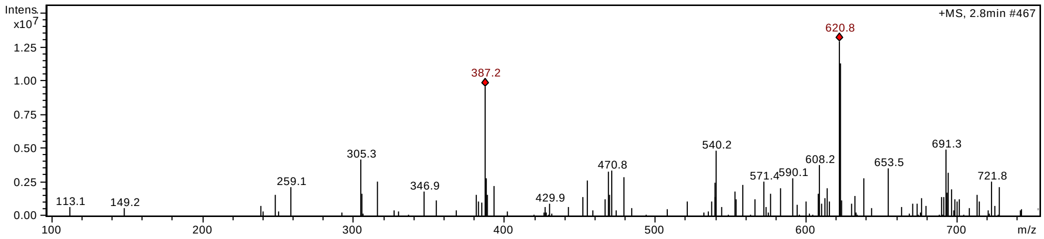

Figure S29. The MS spectrum for the compound **2a.**

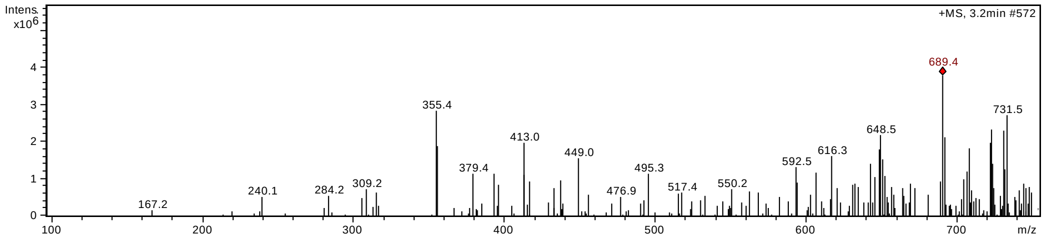

Figure S30. The MS spectrum for the compound **2b.**

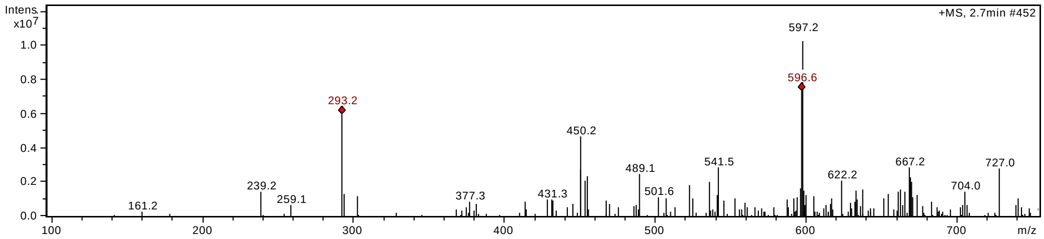

Figure S31. The MS spectrum for the compound **2c.**

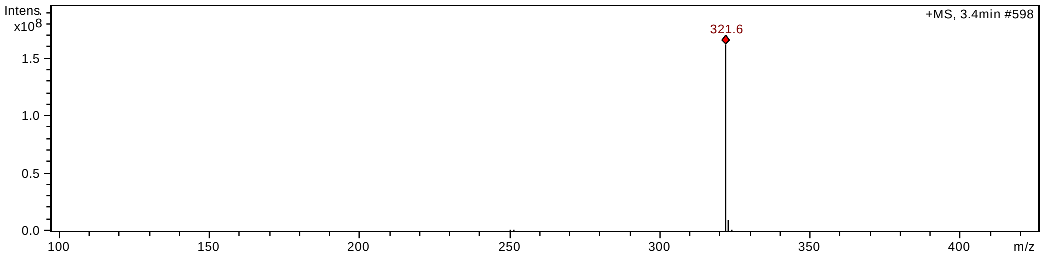

Figure S32. The MS spectrum for the compound **2d.**

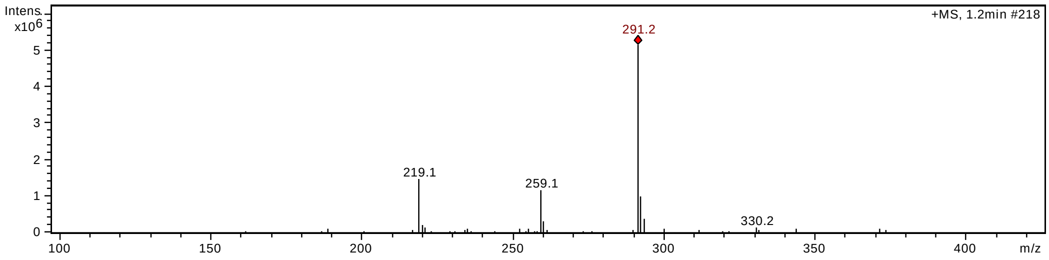

Figure S33. The MS spectrum for the compound **3a.**

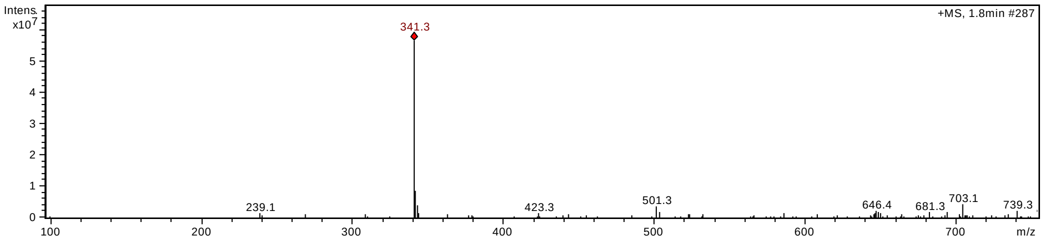

Figure S34. The MS spectrum for the compound **3b.**

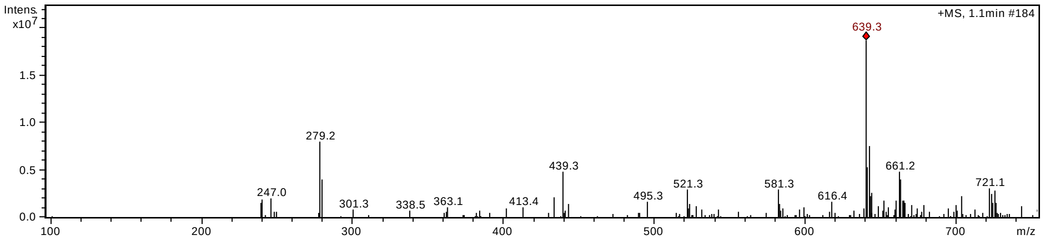

Figure S35. The MS spectrum for the compound **3c.**

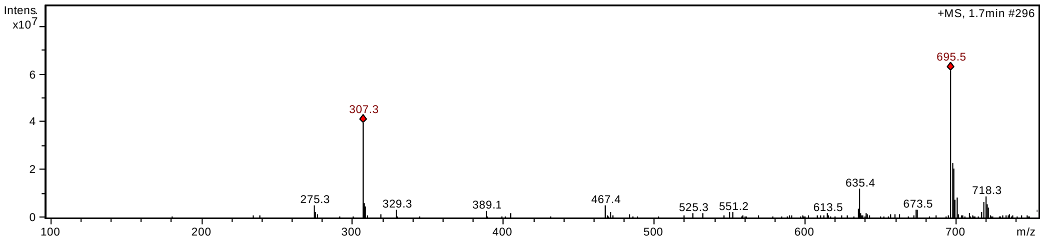

Figure S36. The MS spectrum for the compound **3d.**

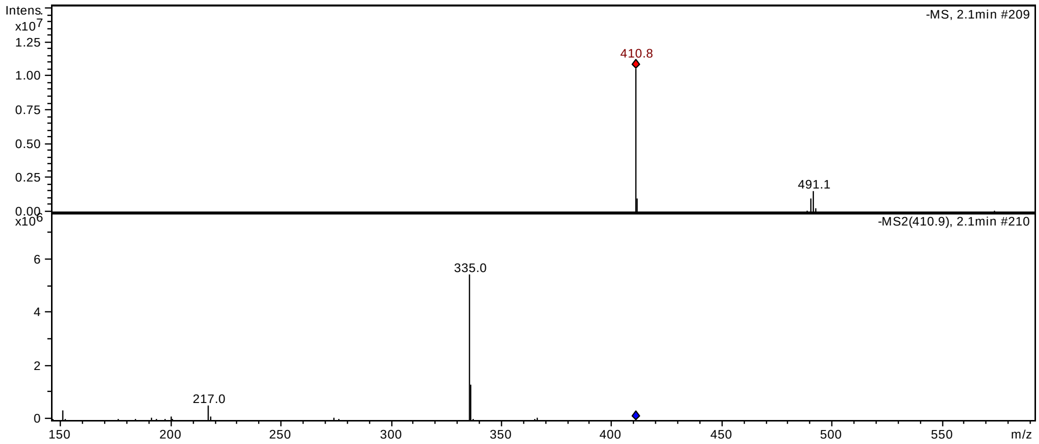

Figure S37. The MS spectrum for the compound **5a.**

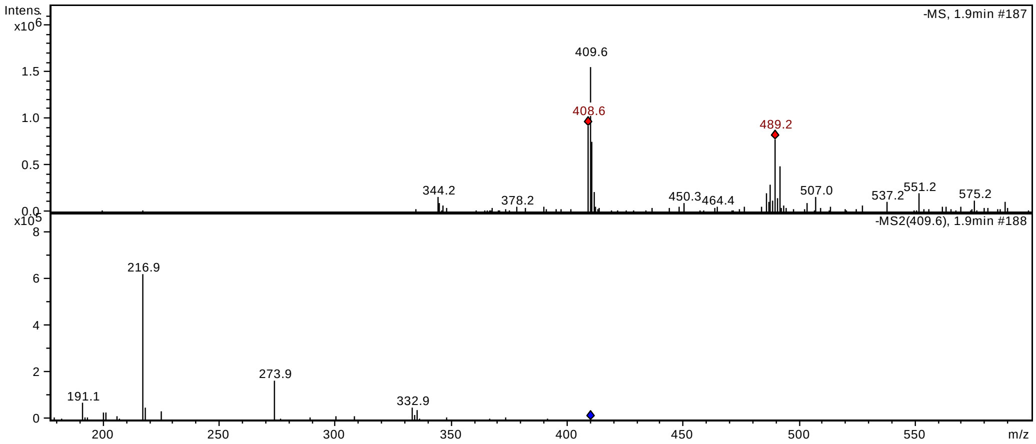

Figure S38. The MS spectrum for the compound **5b.**

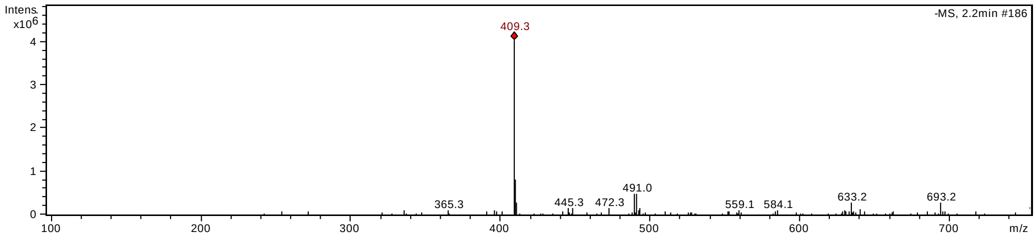

Figure S39. The MS spectrum for the compound **5c**.

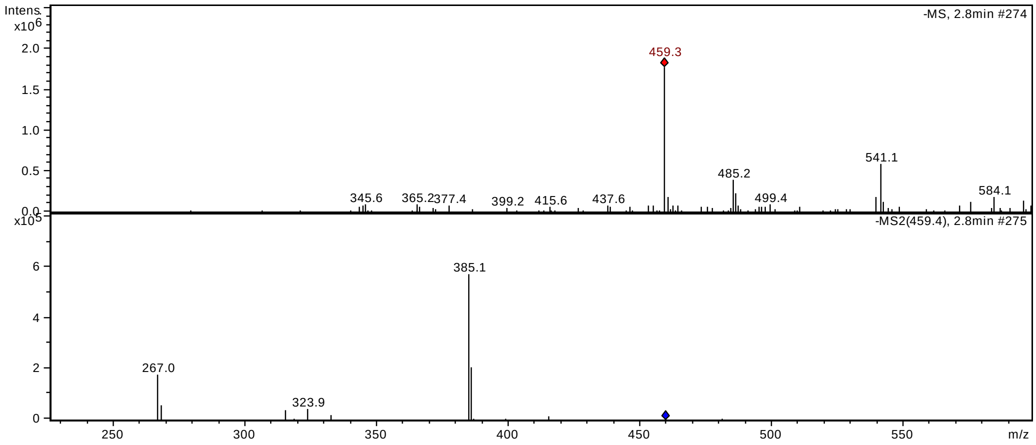

Figure S40. The MS spectrum for the compound **5d**.

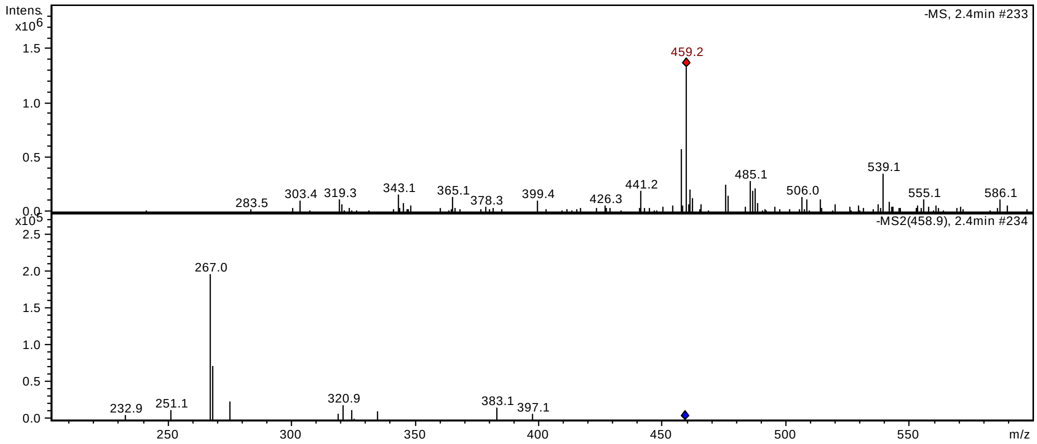

Figure S41. The MS spectrum for the compound **5e**.

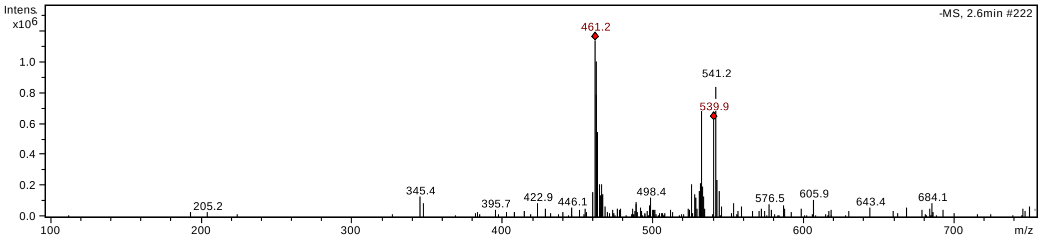

Figure S42. The MS spectrum for the compound **5f.**

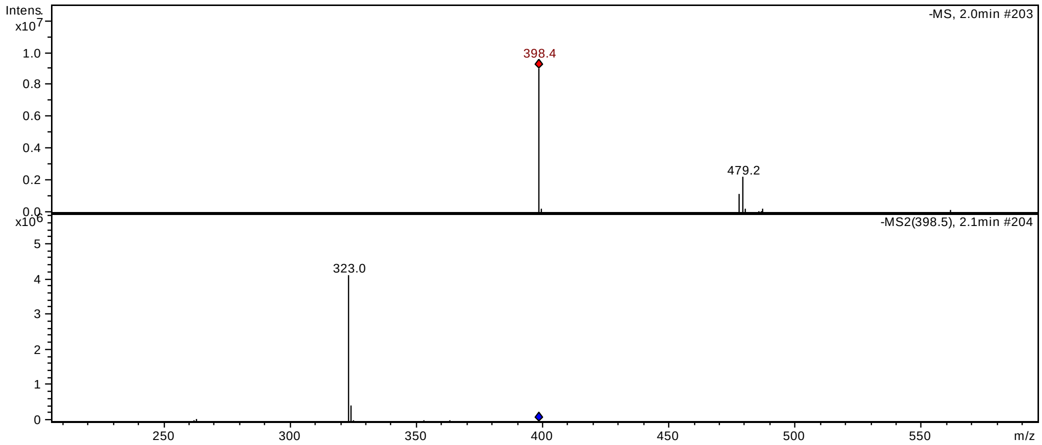

Figure S43. The MS spectrum for the compound **5g.**

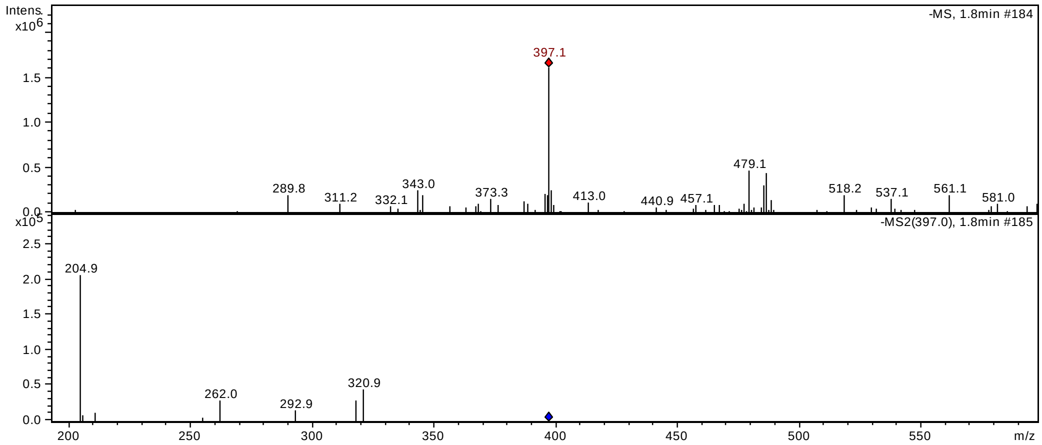

Figure S44. The MS spectrum for the compound **5h.**

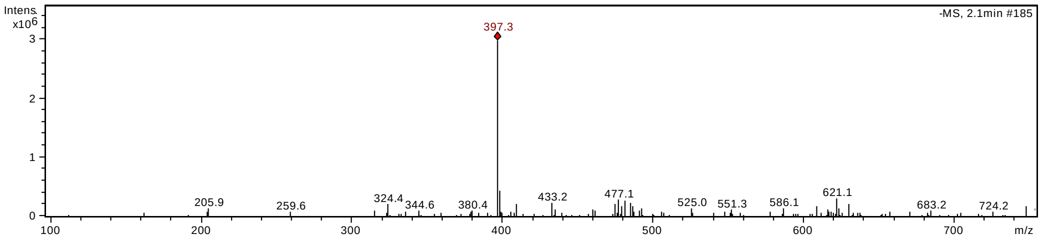

Figure S45. The MS spectrum for the compound **5i.**

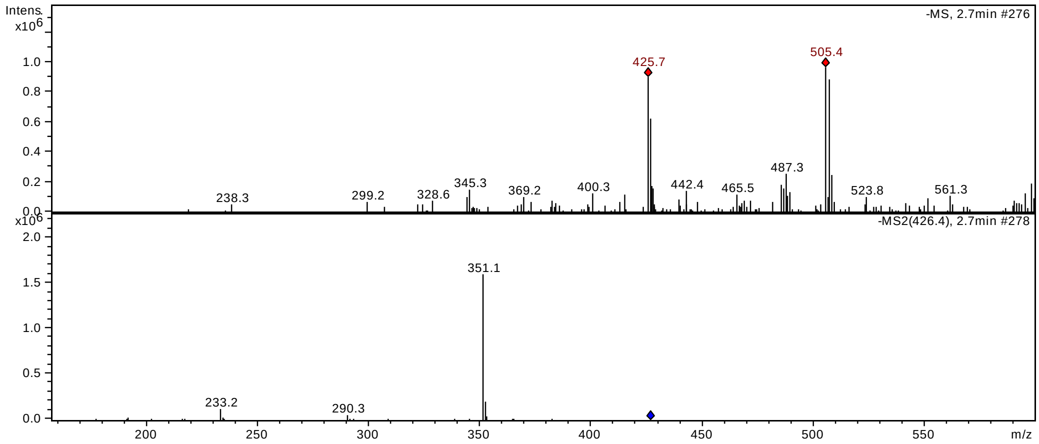

Figure S46. The MS spectrum for the compound **5j.**

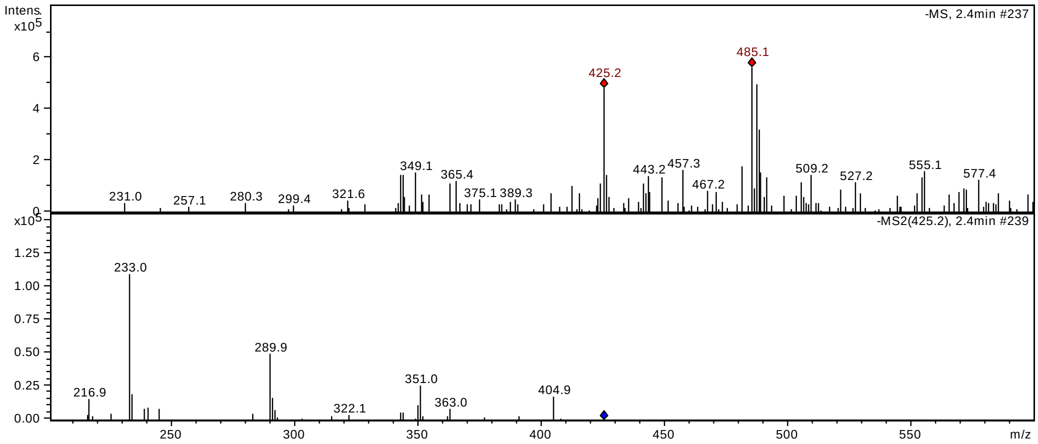

Figure S47. The MS spectrum for the compound **5k.**

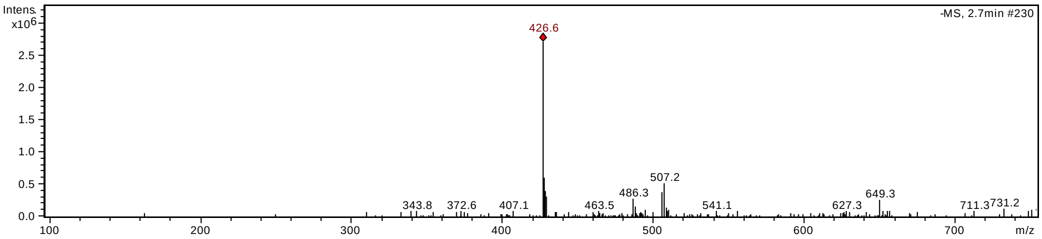

Figure S48. The MS spectrum for the compound **5l.**

1.3. The ¹H-NMR spectrum

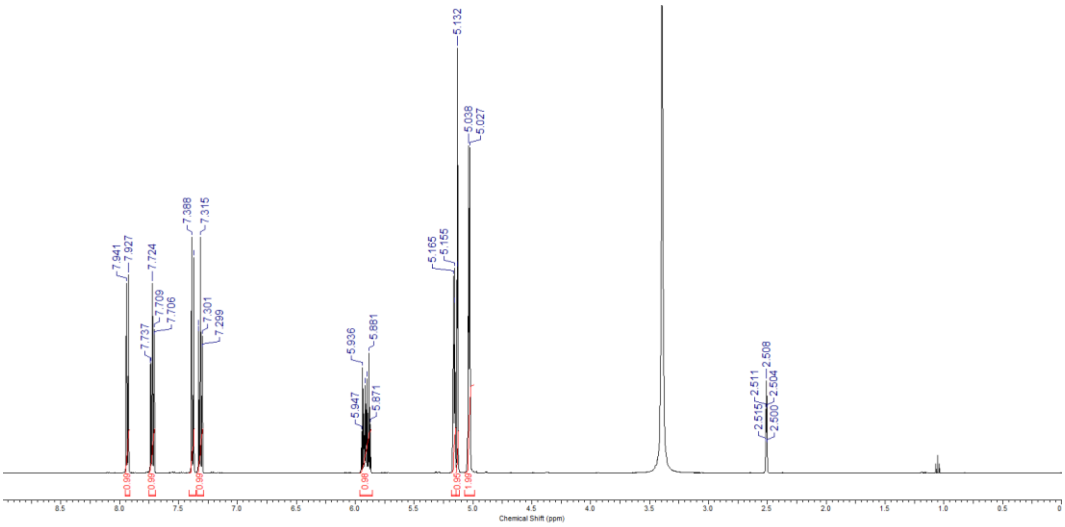

Figure S49. The ¹H-NMR spectrum for the compound **1a.**

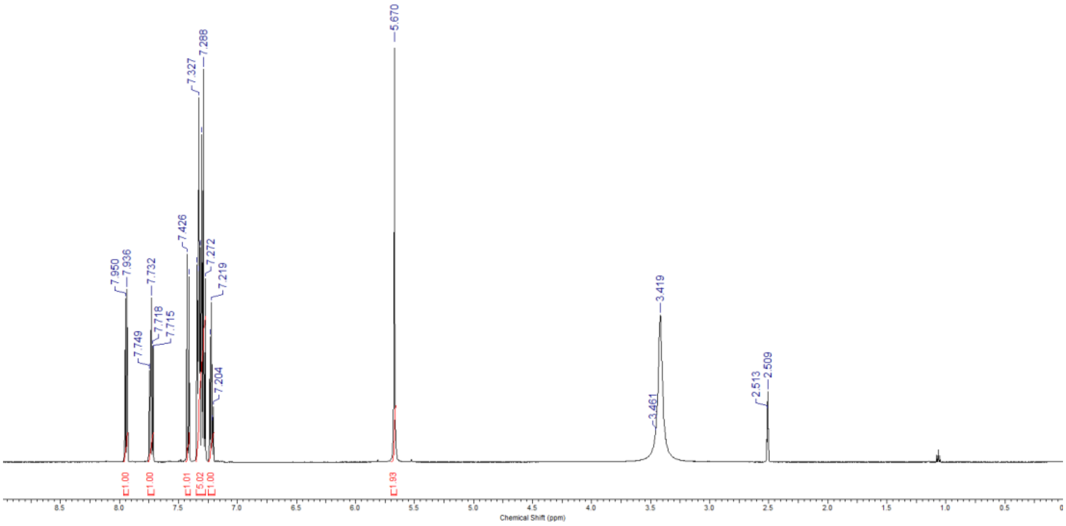

Figure S50. The ¹H-NMR spectrum for the compound **1b.**

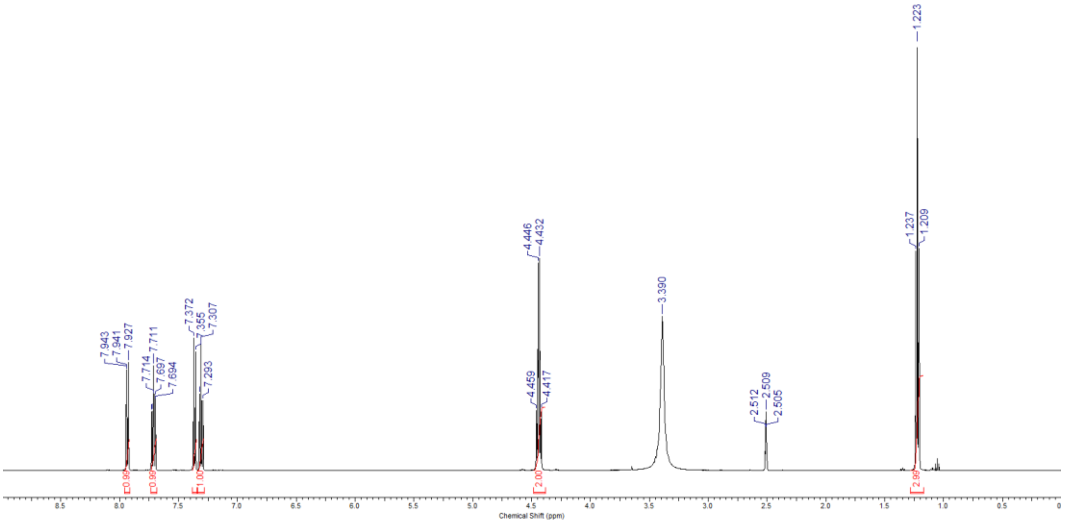

Figure S51. The ¹H-NMR spectrum for the compound **1c.**

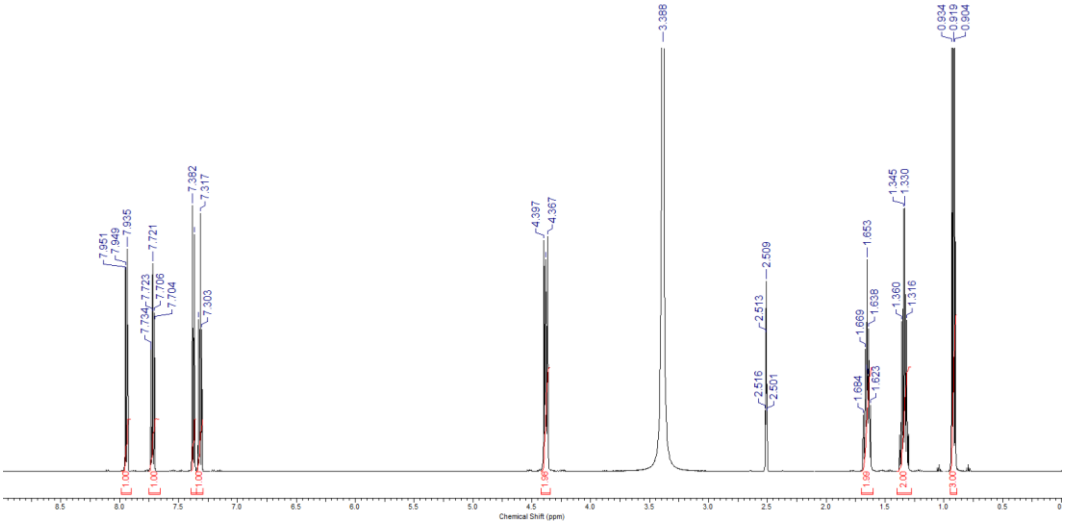

Figure S52. The ¹H-NMR spectrum for the compound **1d.**

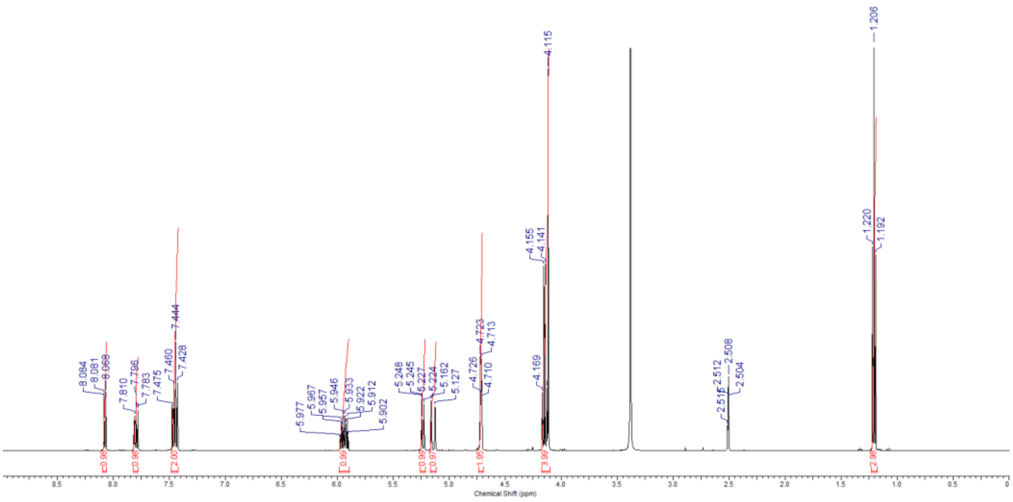

Figure S53. The ^{1}H-NMR spectrum for the compound **2a.**

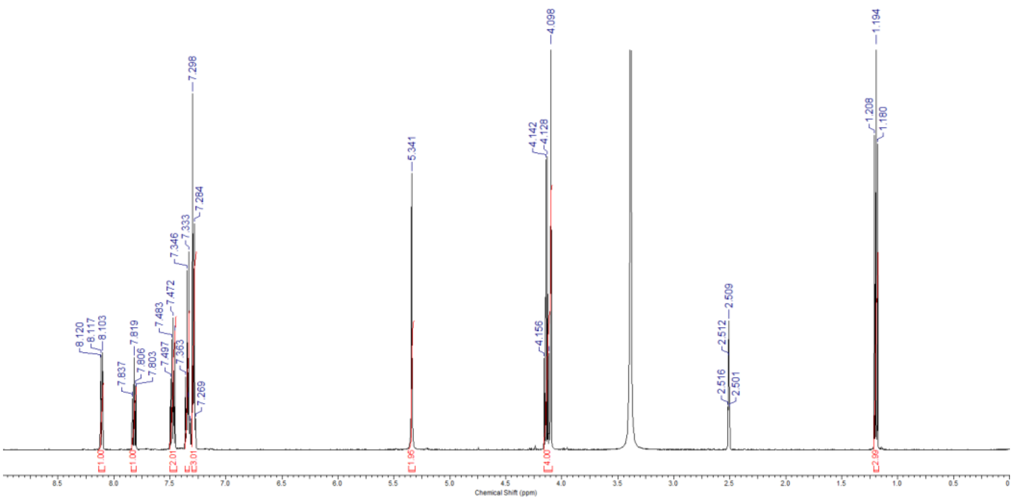

Figure S54. The ^{1}H-NMR spectrum for the compound **2b.**

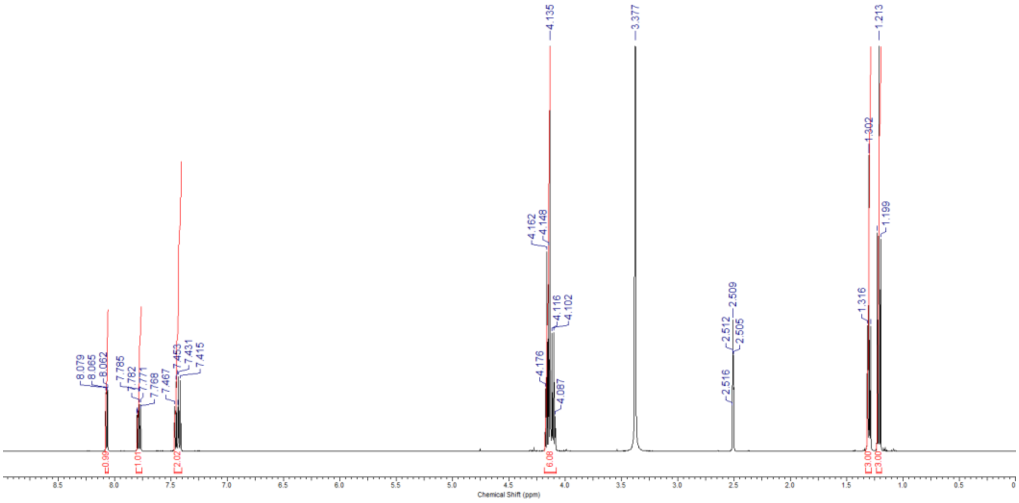

Figure S55. The ^{1}H-NMR spectrum for the compound **2c.**

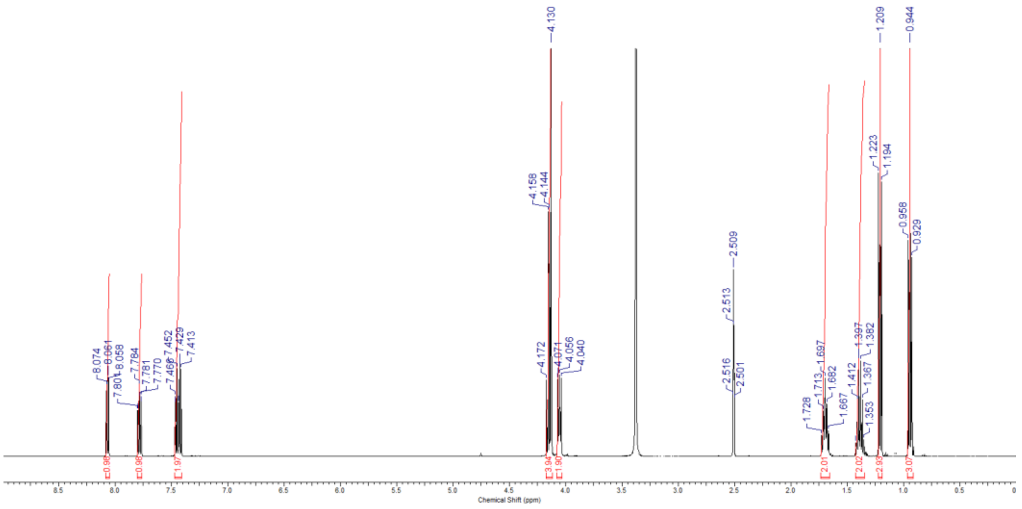

Figure S56. The ^{1}H-NMR spectrum for the compound **2d.**

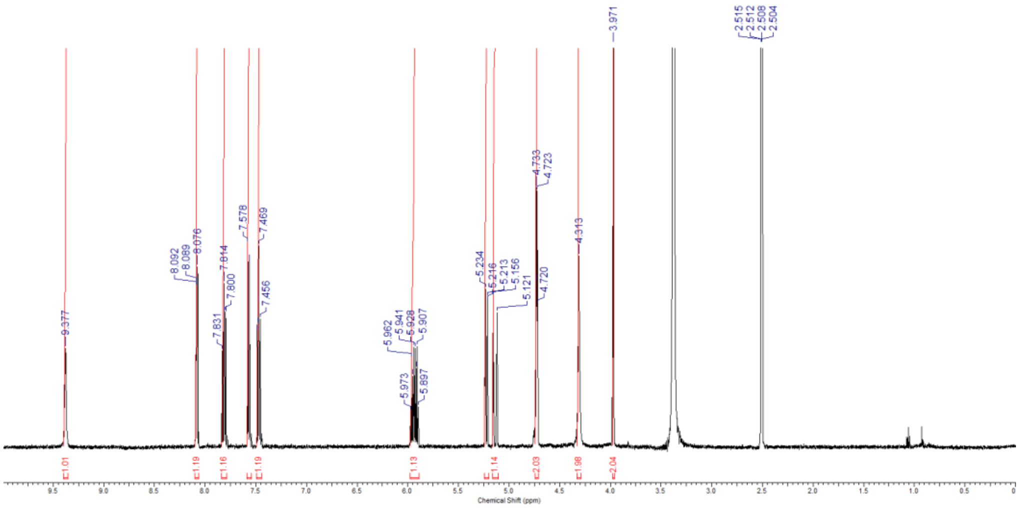

Figure S57. The ^{1}H-NMR spectrum for the compound **3a.**

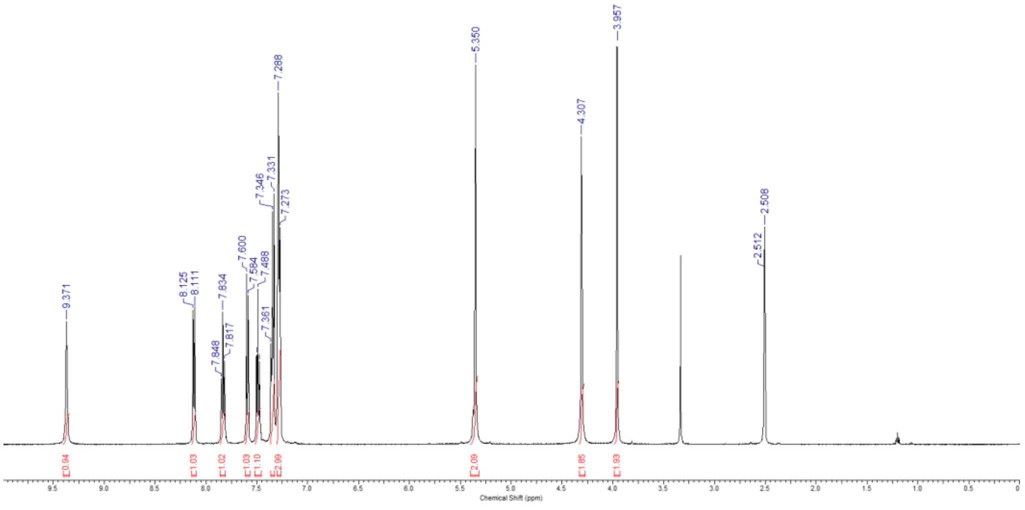

Figure S58. The ^{1}H-NMR spectrum for the compound **3b.**

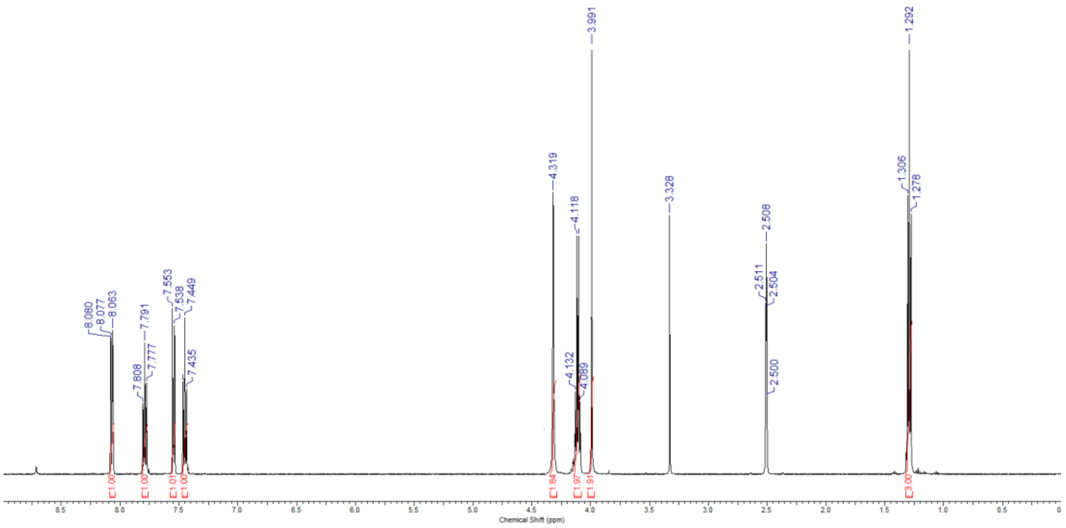

Figure S59. The ^{1}H-NMR spectrum for the compound **3c.**

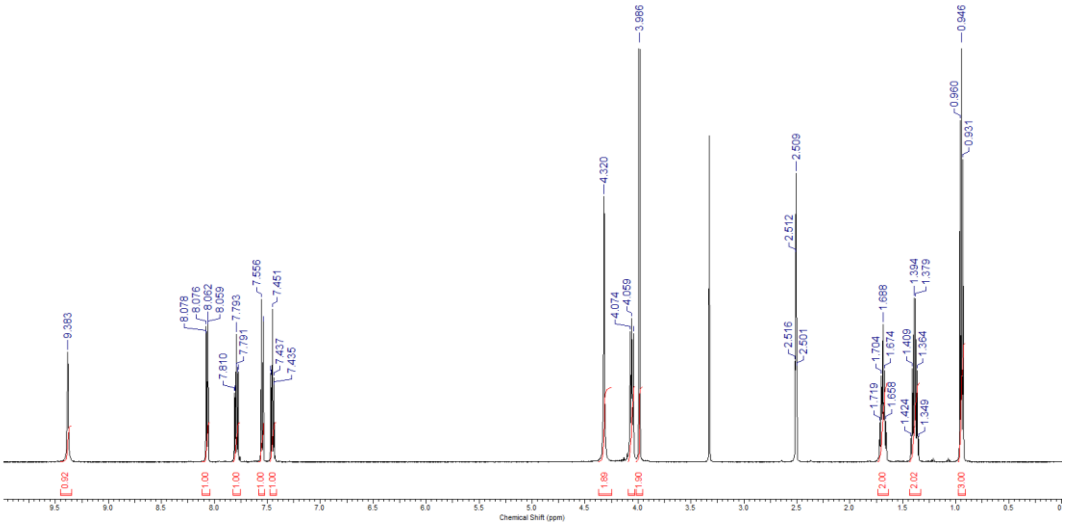

Figure S60. The ^{1}H-NMR spectrum for the compound **3d.**

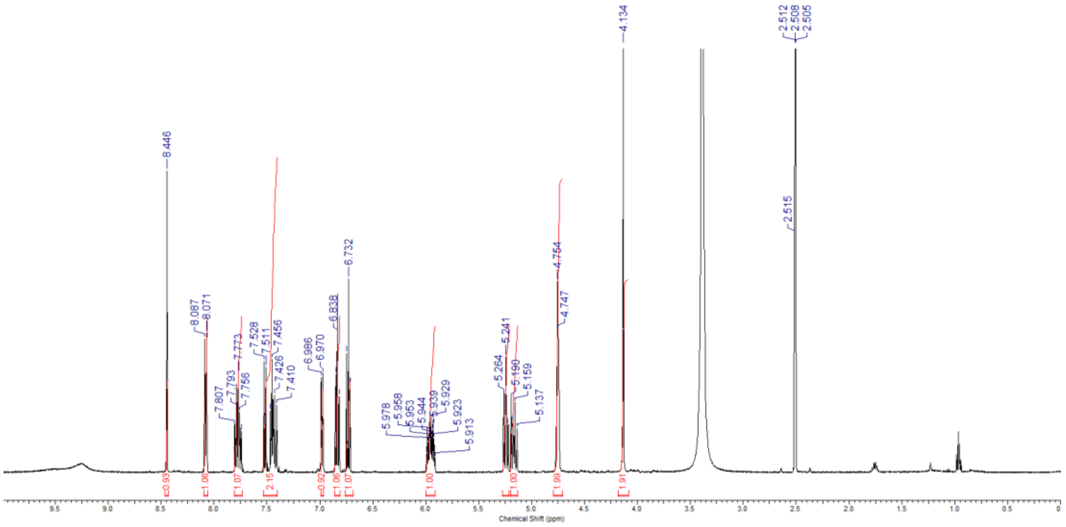

Figure S61. The ^{1}H-NMR spectrum for the compound **5a.**

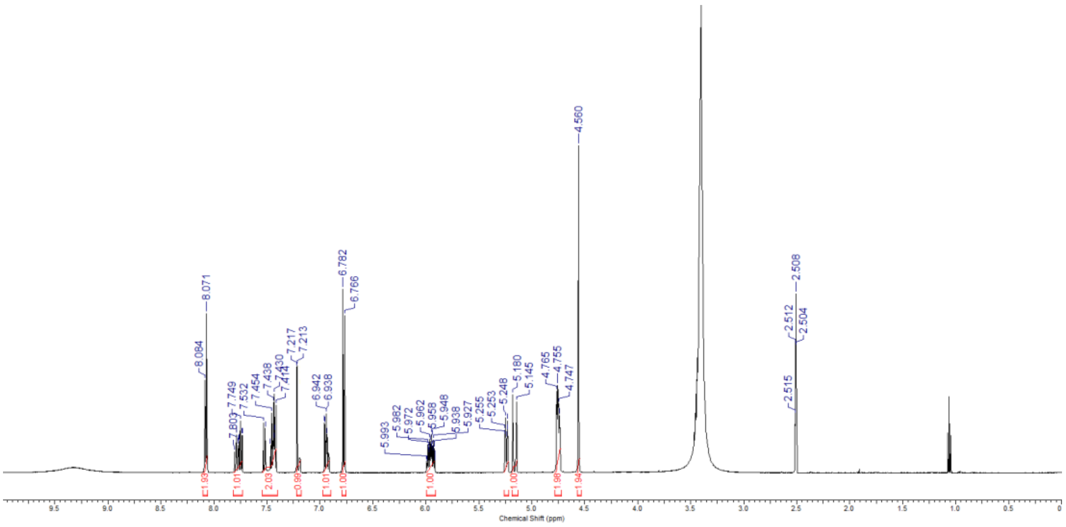

Figure S62. The ^{1}H-NMR spectrum for the compound **5b.**

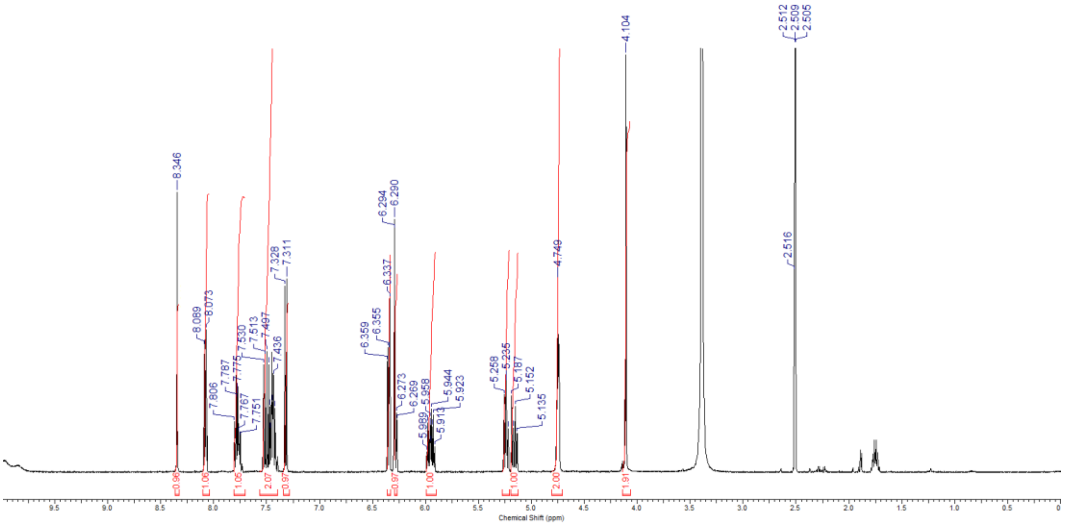

Figure S63. The ^{1}H-NMR spectrum for the compound **5c.**

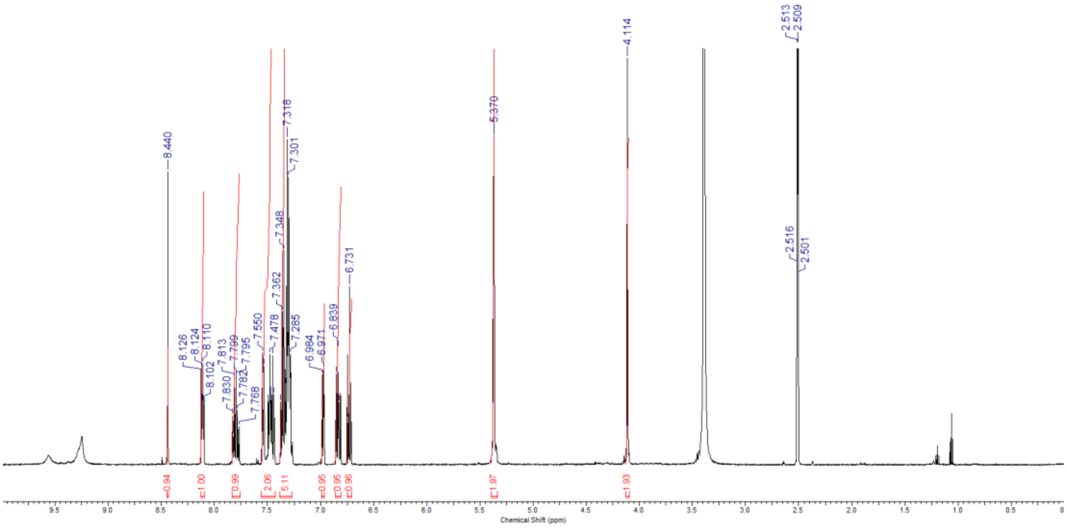

Figure S64. The ^{1}H-NMR spectrum for the compound **5d.**

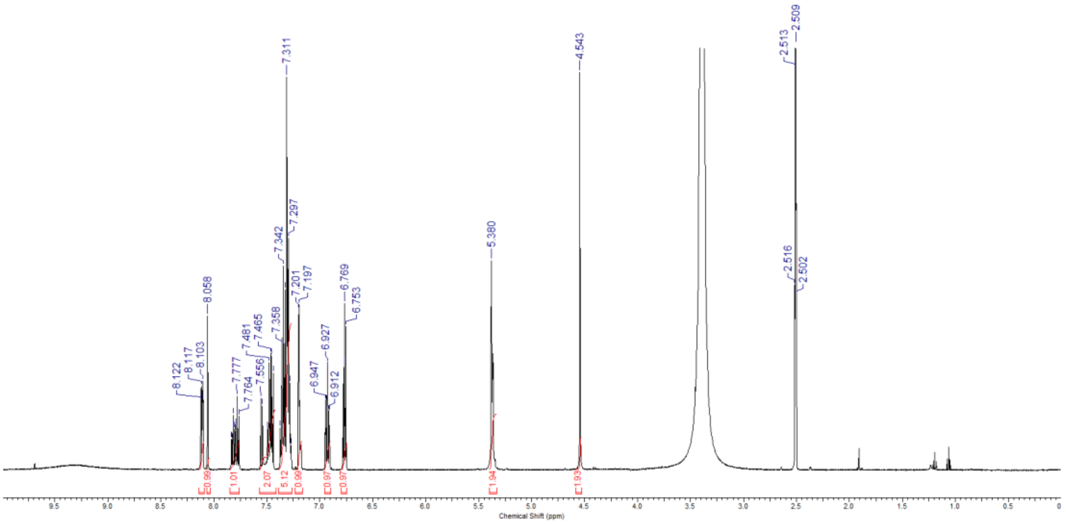

Figure S65. The ^{1}H-NMR spectrum for the compound **5e.**

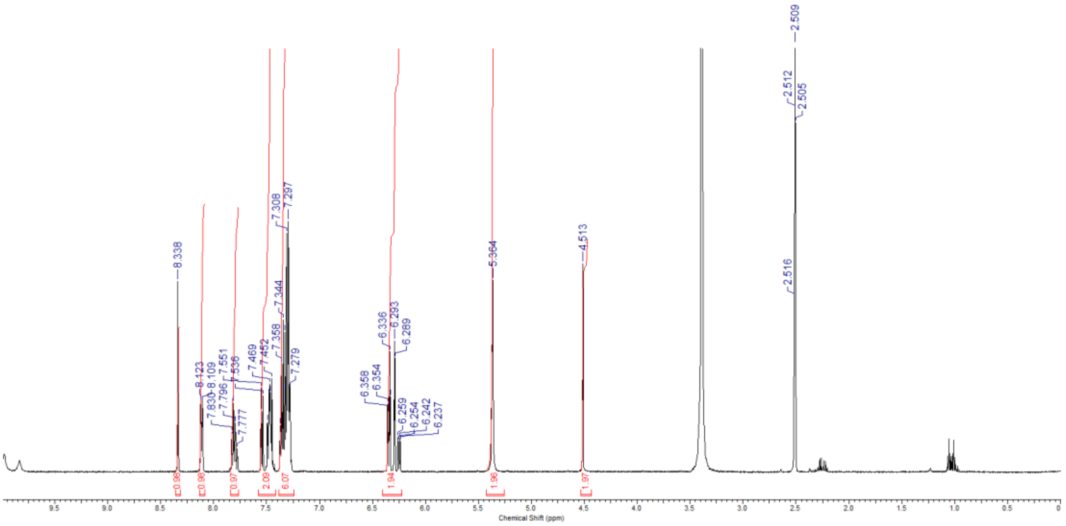

Figure S66. The ^{1}H-NMR spectrum for the compound **5f.**

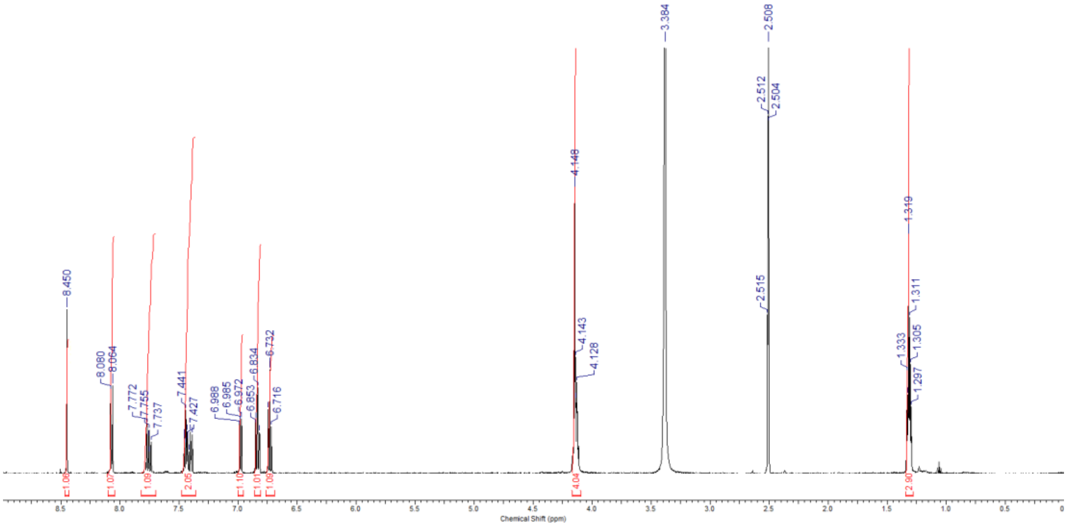

Figure S67. The ^{1}H-NMR spectrum for the compound **5g.**

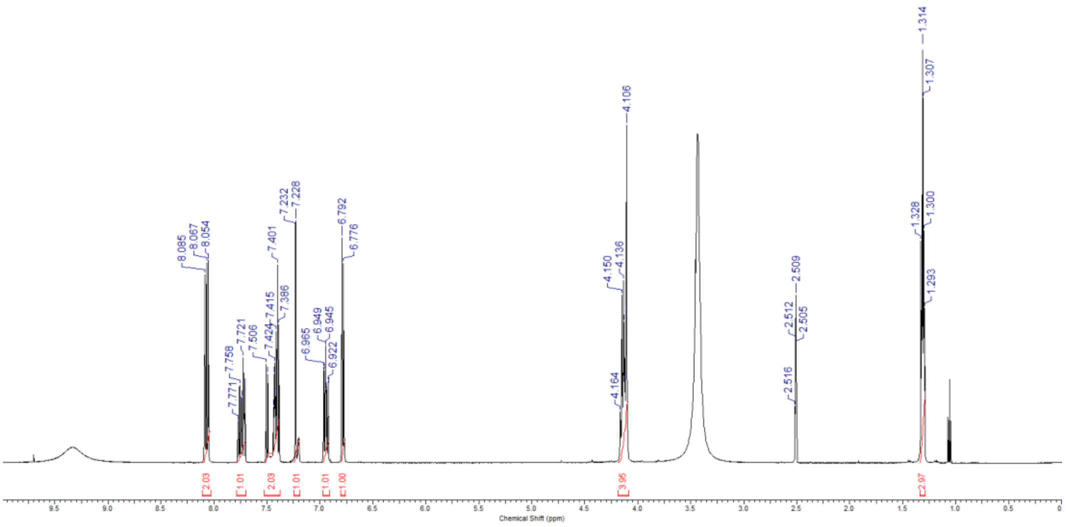

Figure S68. The ^{1}H-NMR spectrum for the compound **5h.**

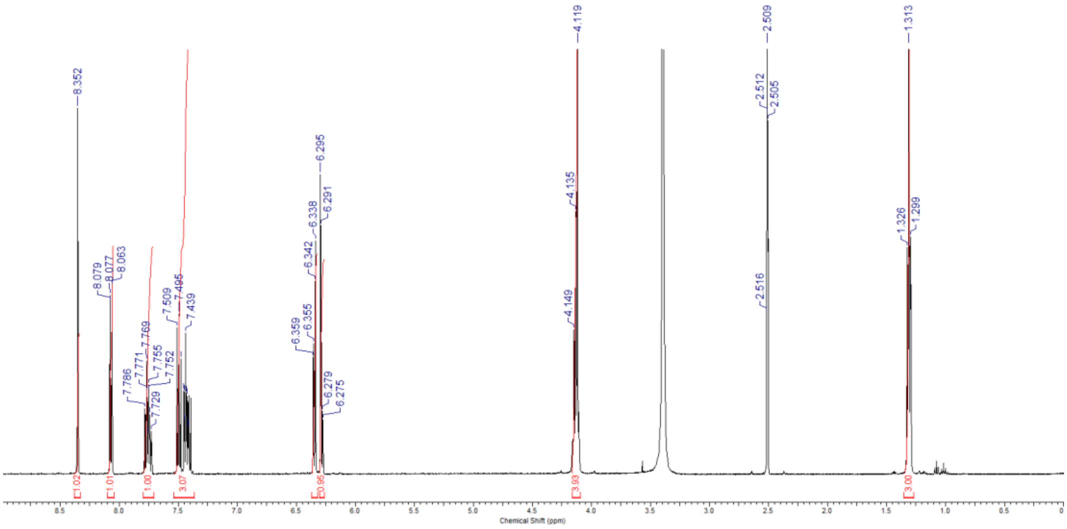

Figure S69. The ^{1}H-NMR spectrum for the compound **5i.**

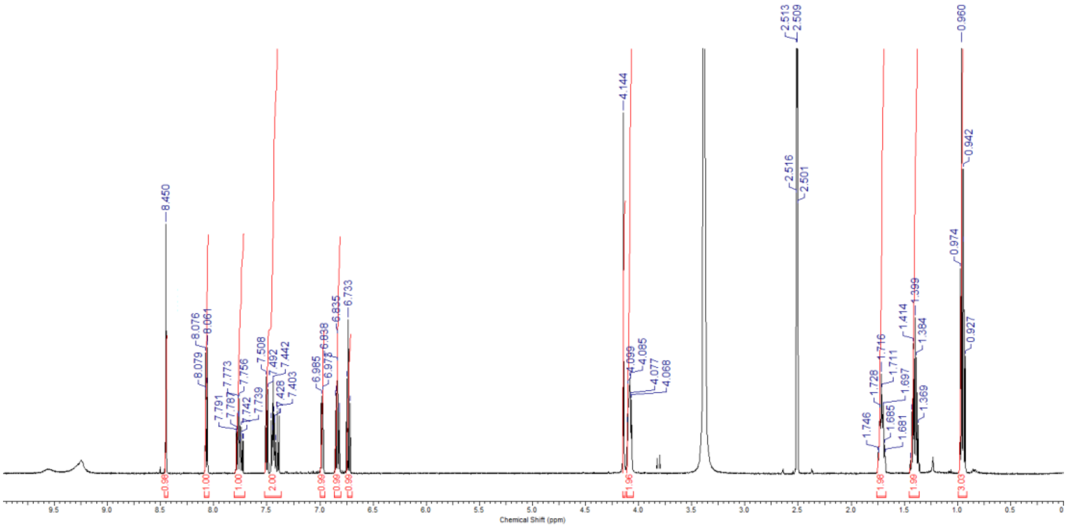

Figure S70. The ^{1}H-NMR spectrum for the compound **5j.**

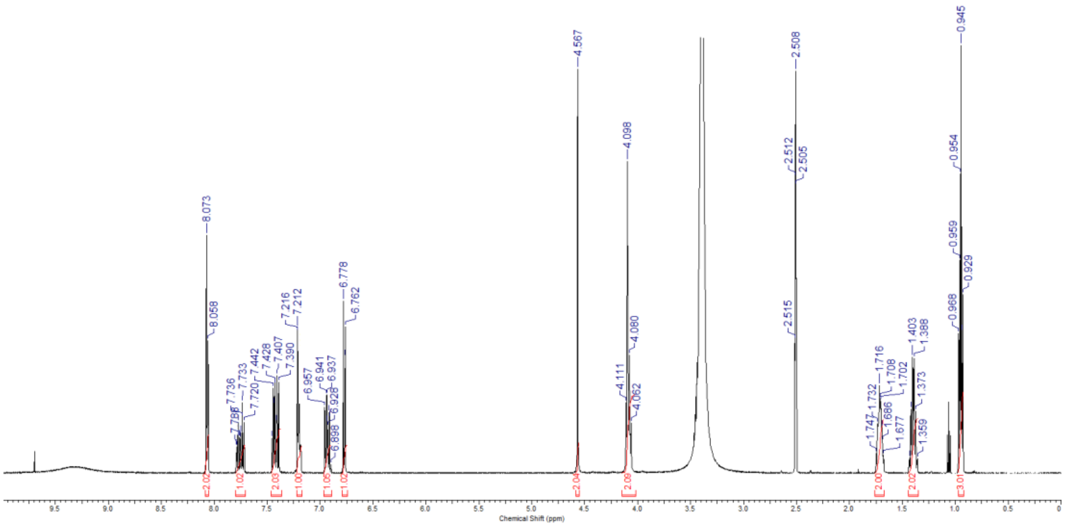

Figure S71. The ^{1}H-NMR spectrum for the compound **5k.**

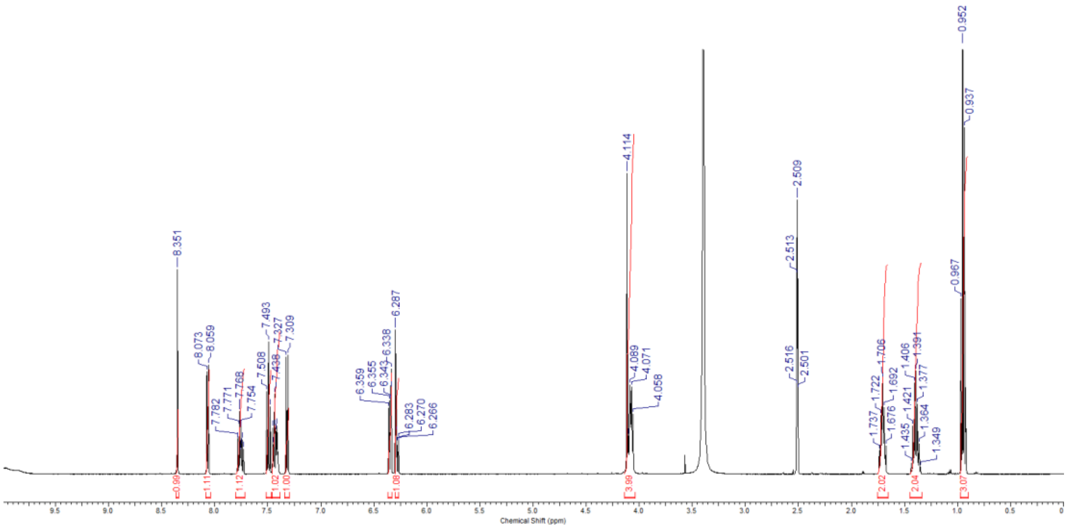

Figure S72. The ^{1}H-NMR spectrum for the compound **5l.**

1.4. *The ^{13}C-NMR spectra*

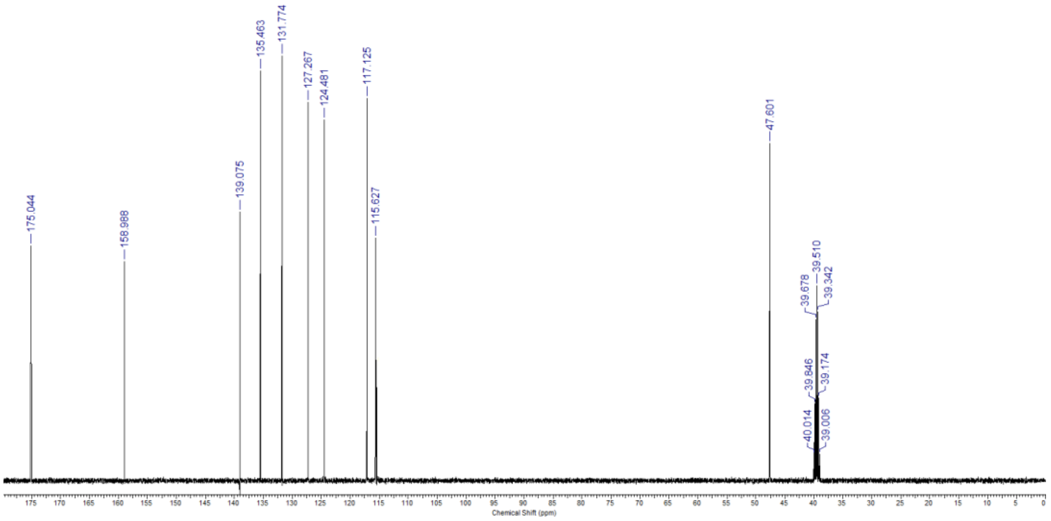

Figure S73. The ^{13}C-NMR spectrum for the compound **1a.**

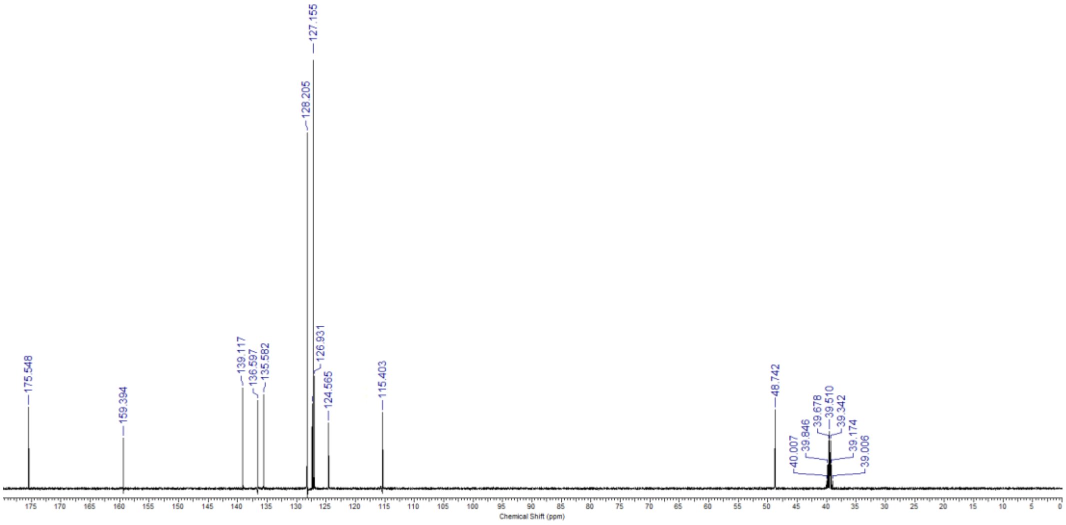

Figure S74. The ^{13}C-NMR spectrum for the compound **1b.**

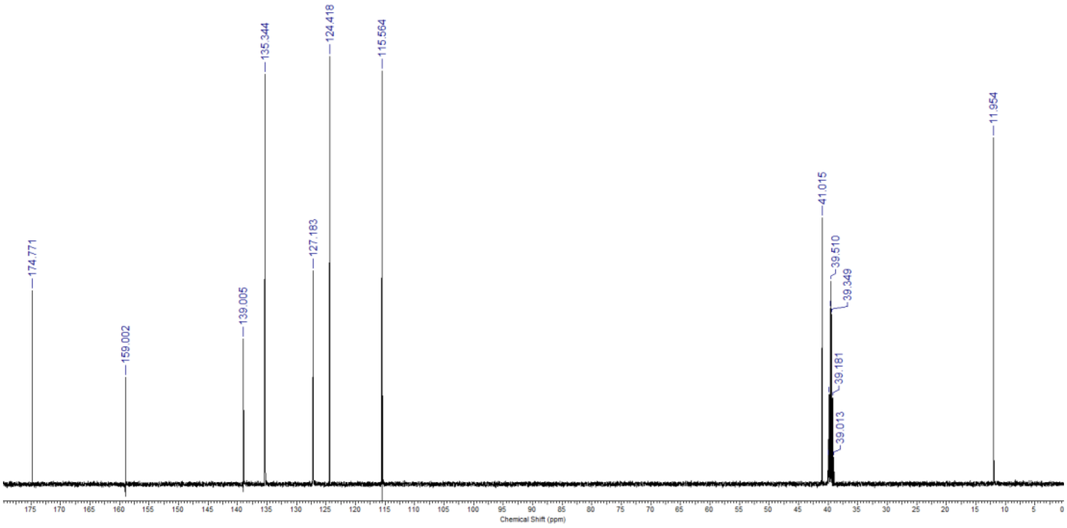

Figure S75. The ^{13}C-NMR spectrum for the compound **1c.**

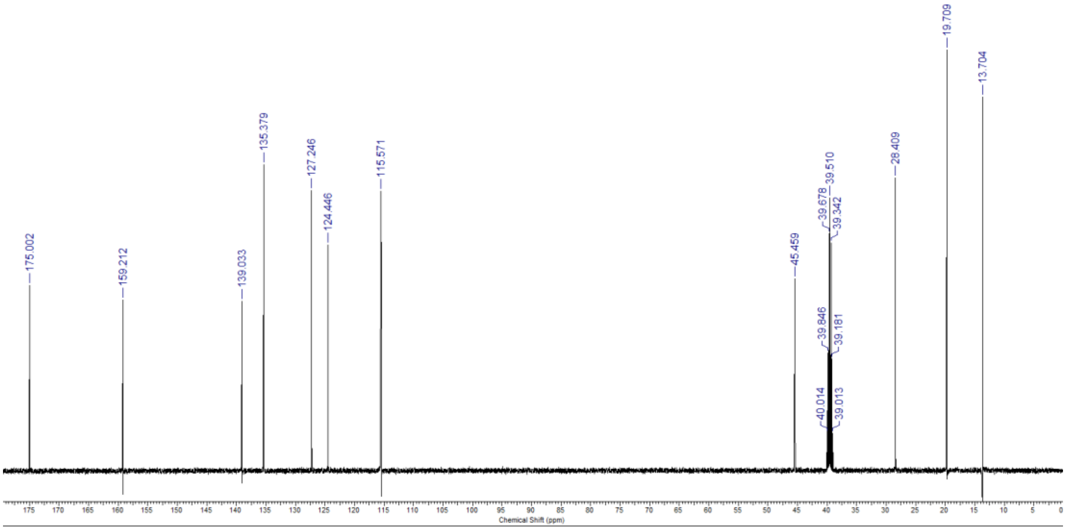

Figure S76. The ^{13}C-NMR spectrum for the compound **1d.**

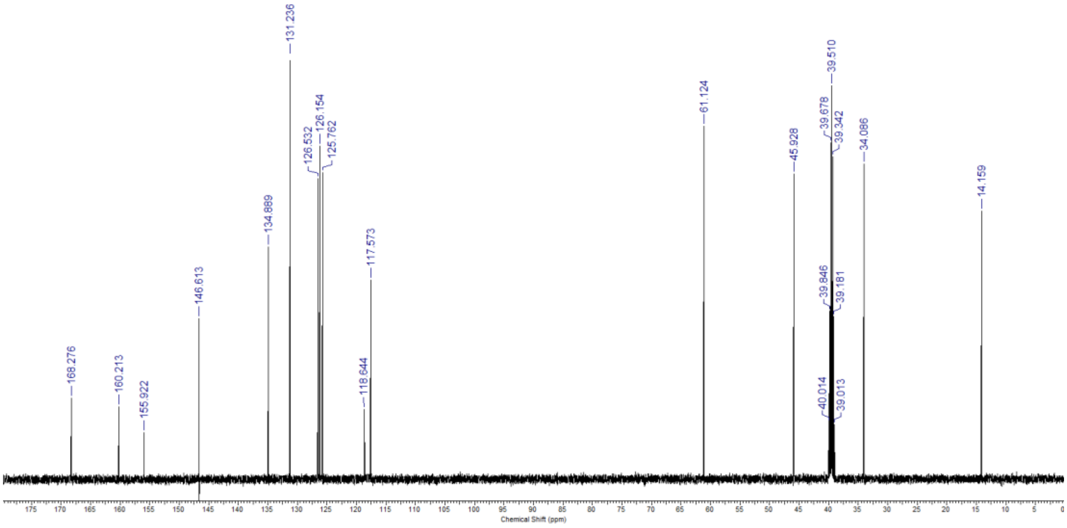

Figure S77. The ^{13}C-NMR spectrum for the compound **2a.**

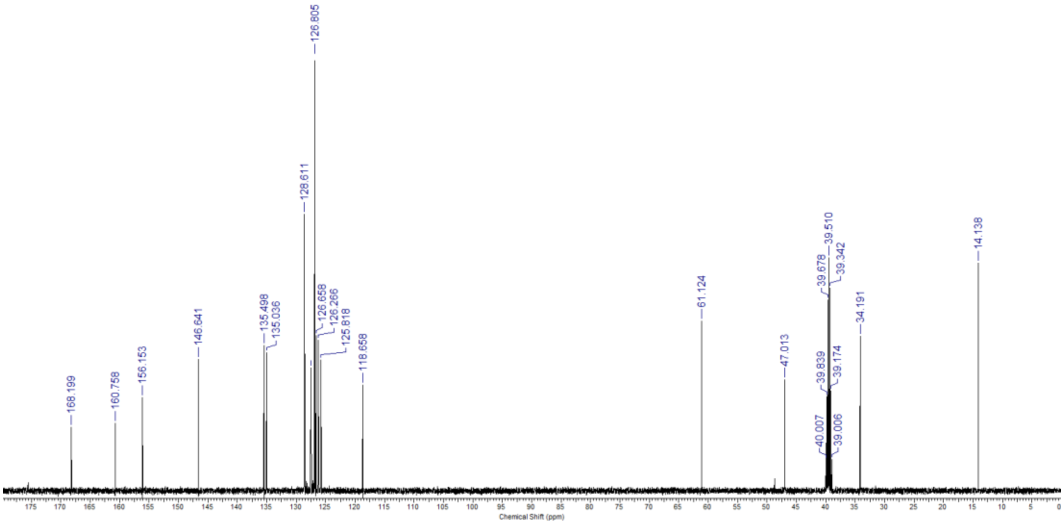

Figure S78. The ^{13}C-NMR spectrum for the compound **2b.**

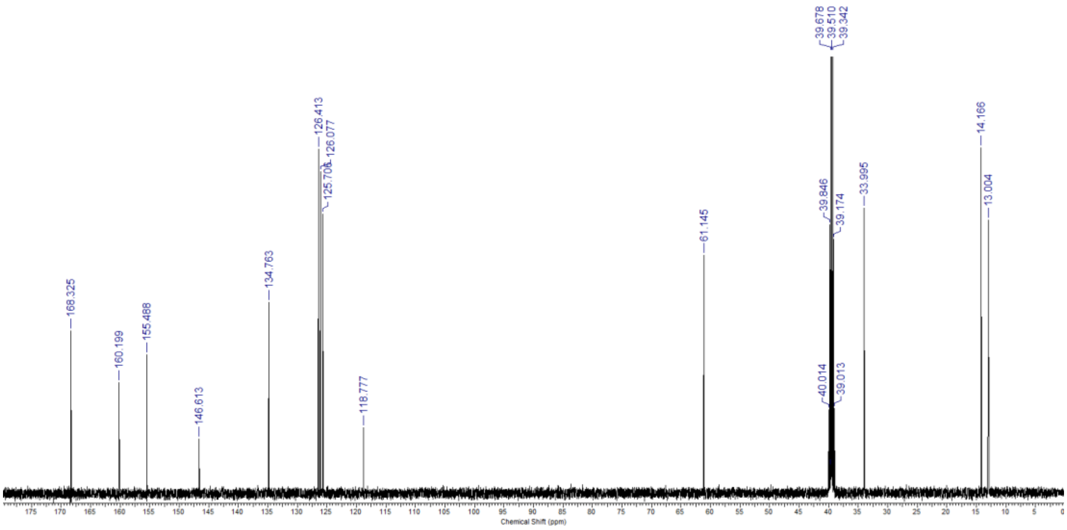

Figure S79. The ^{13}C-NMR spectrum for the compound **2c.**

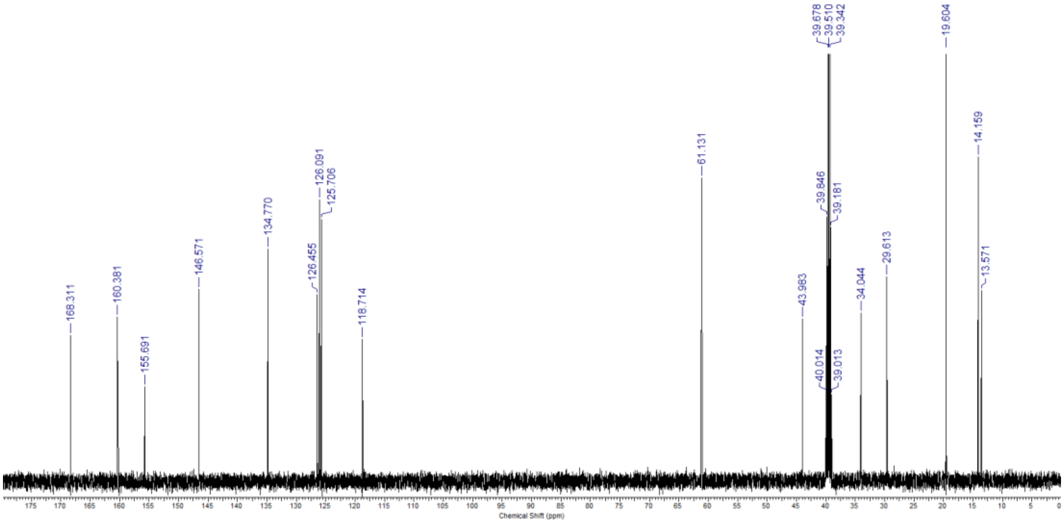

Figure S80. The ^{13}C-NMR spectrum for the compound **2d.**

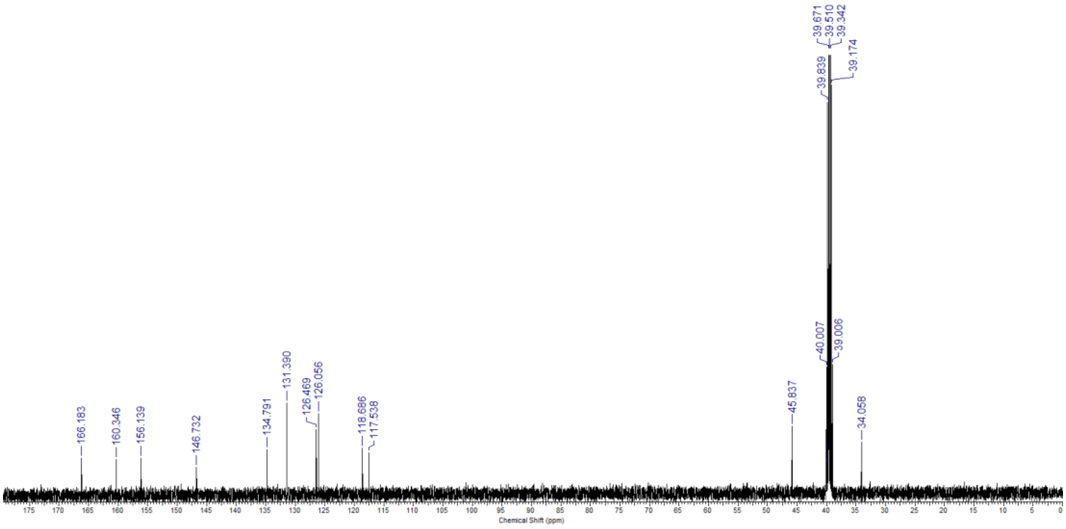

Figure S81. The ^{13}C-NMR spectrum for the compound **3a.**

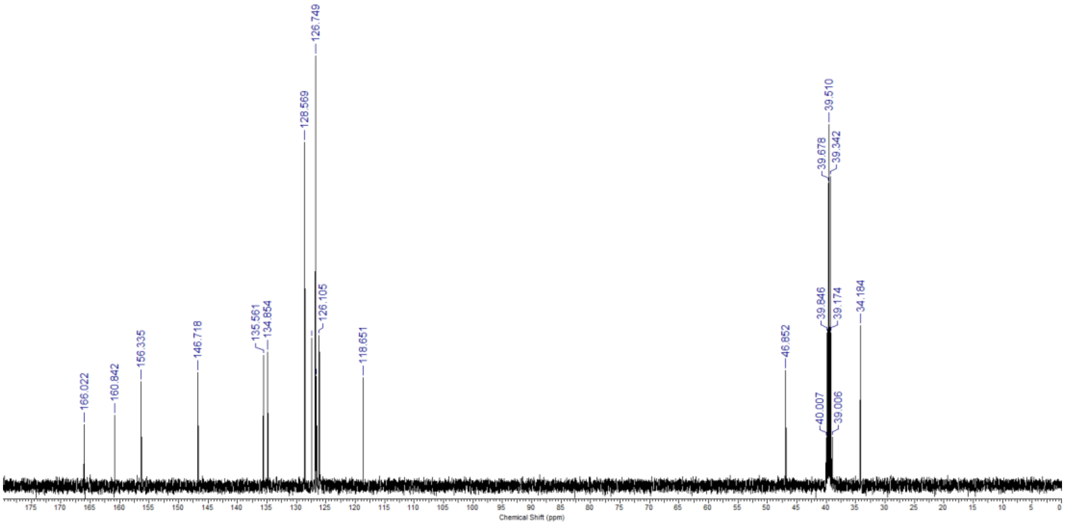

Figure S82. The ^{13}C-NMR spectrum for the compound **3b.**

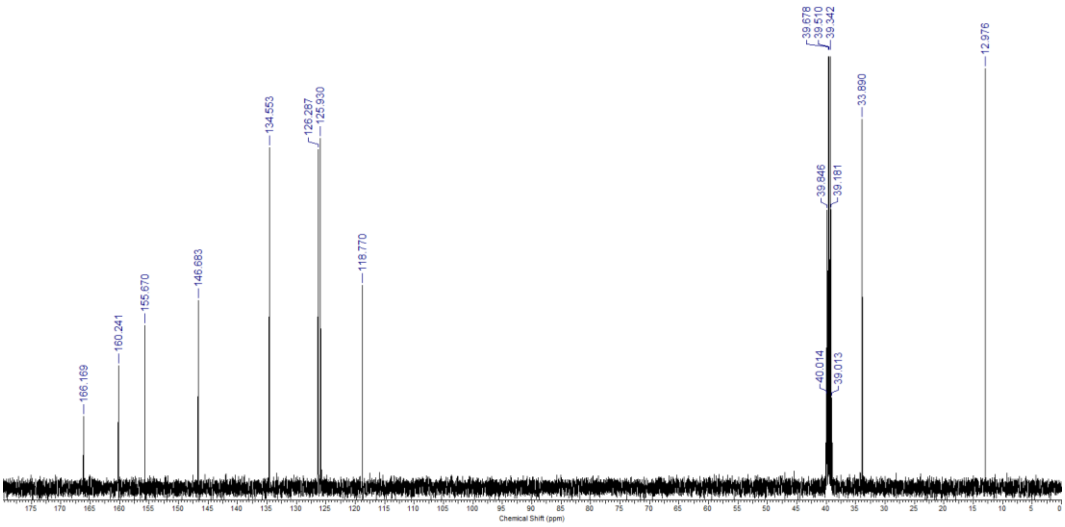

Figure S83. The ^{13}C-NMR spectrum for the compound **3c.**

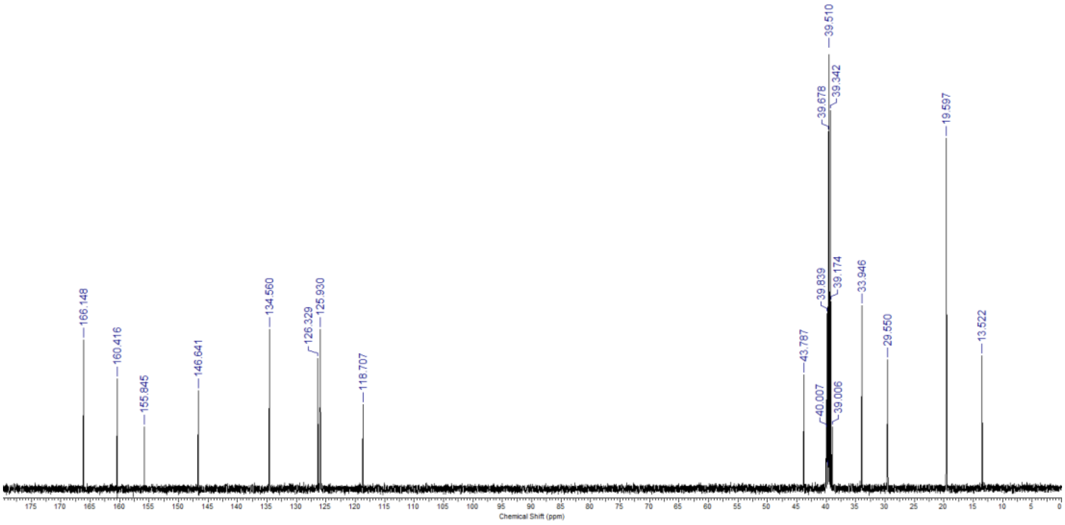

Figure S84. The ^{13}C-NMR spectrum for the compound **3d.**

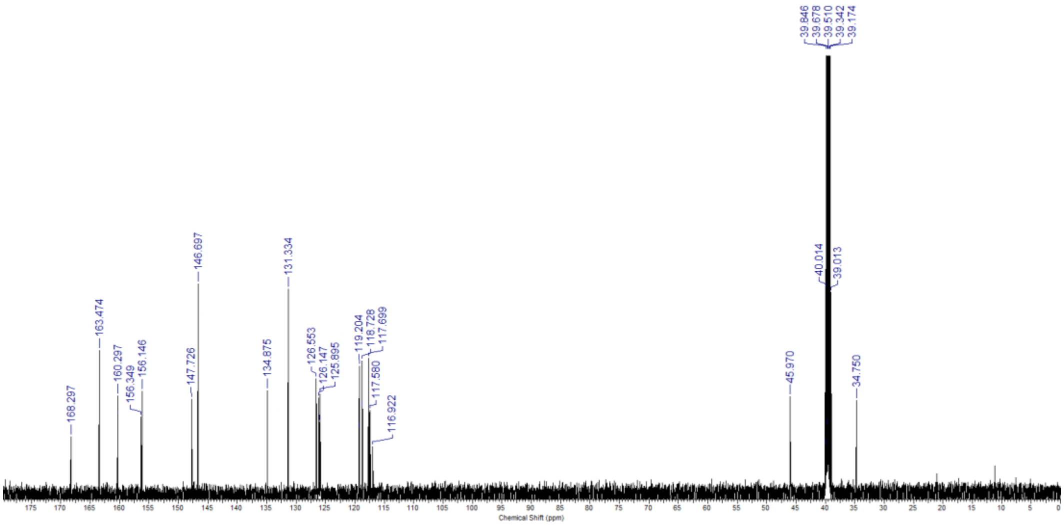

Figure S85. The ^{13}C-NMR spectrum for the compound **5a.**

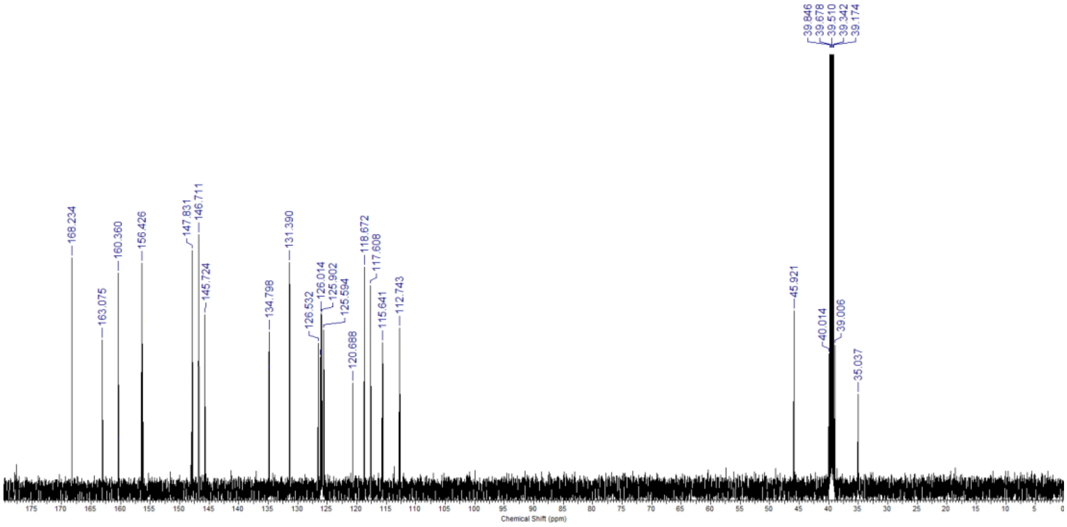

Figure S86. The ^{13}C-NMR spectrum for the compound **5b.**

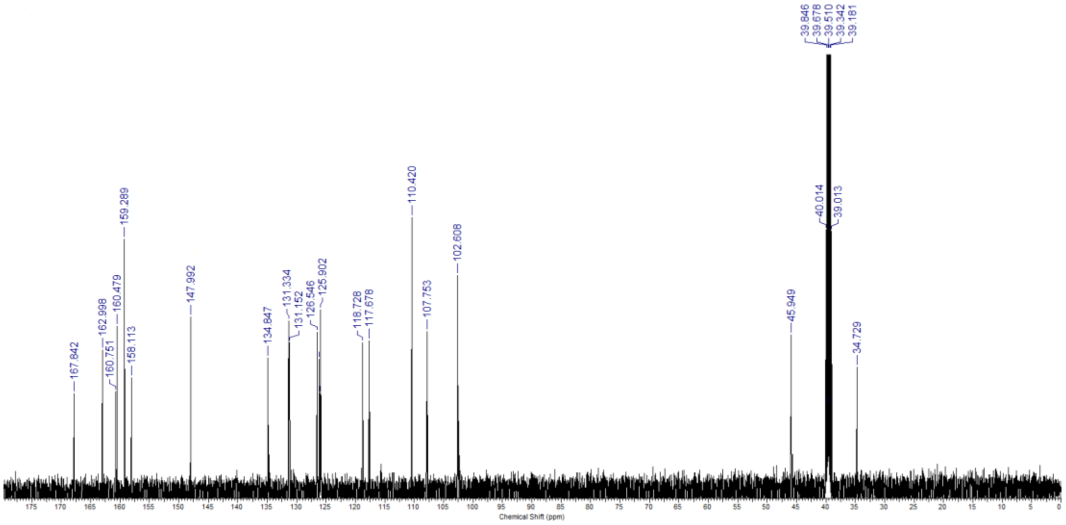

Figure S87. The ^{13}C-NMR spectrum for the compound **5c.**

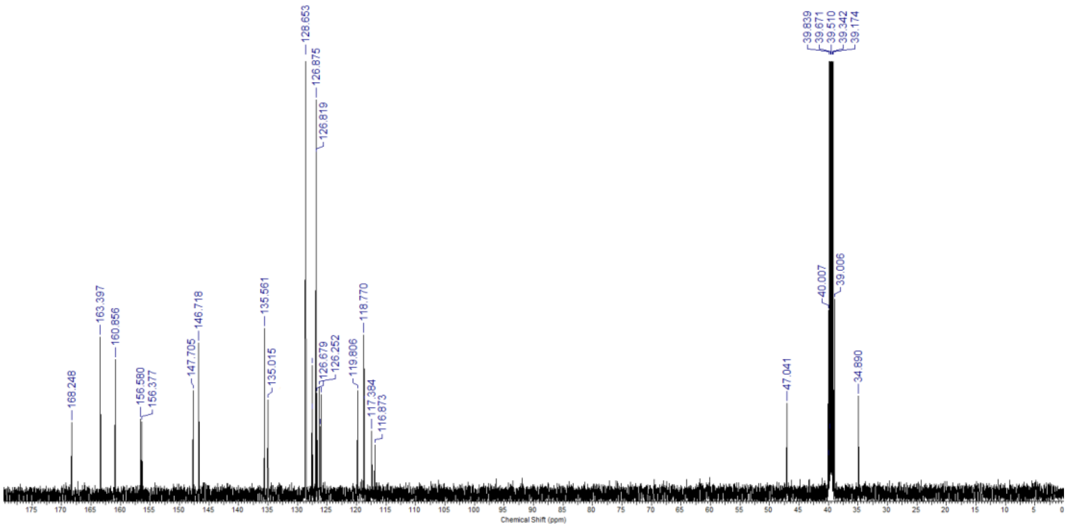

Figure S88. The ^{13}C-NMR spectrum for the compound **5d.**

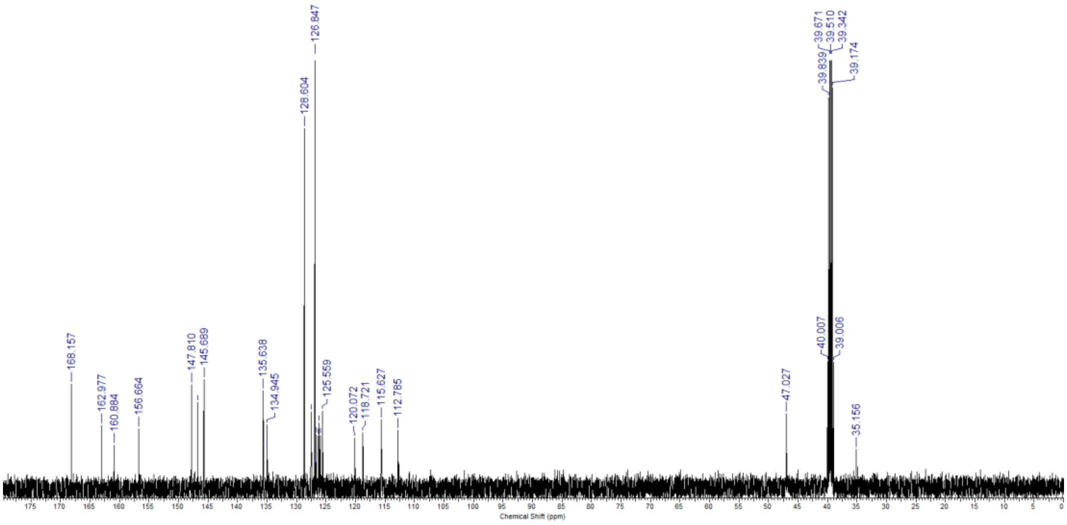

Figure S89. The ^{13}C-NMR spectrum for the compound **5e.**

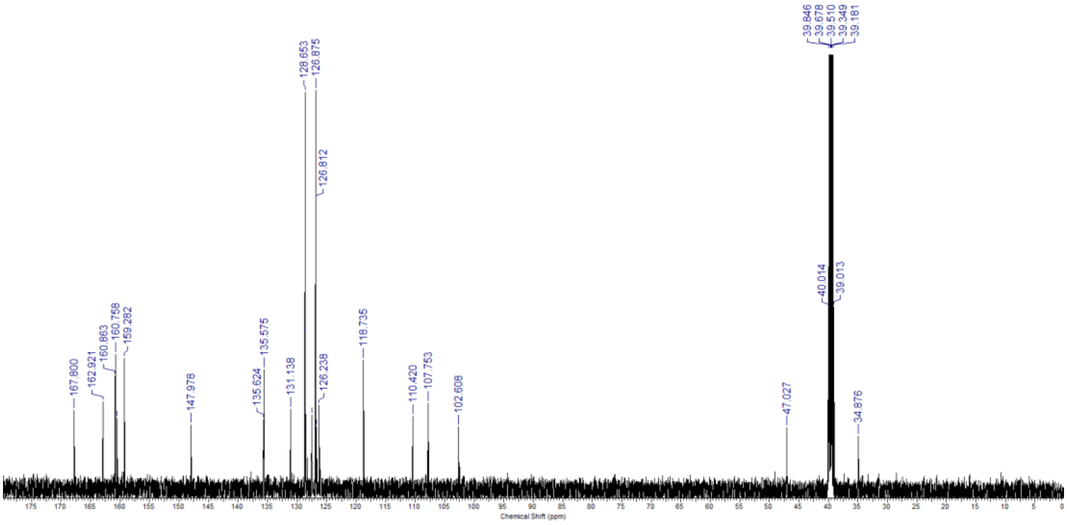

Figure S90. The ^{13}C-NMR spectrum for the compound **5f.**

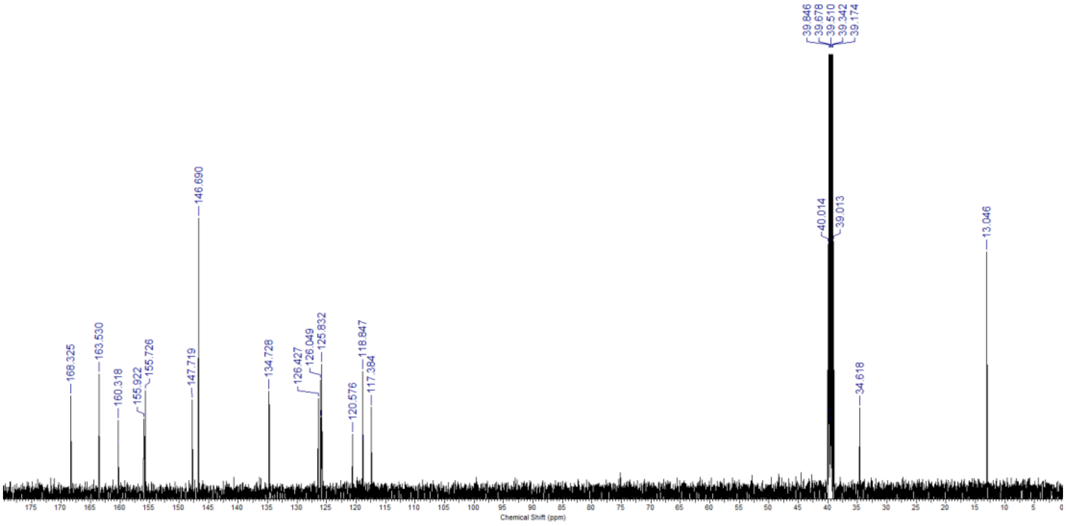

Figure S91. The ^{13}C-NMR spectrum for the compound **5g.**

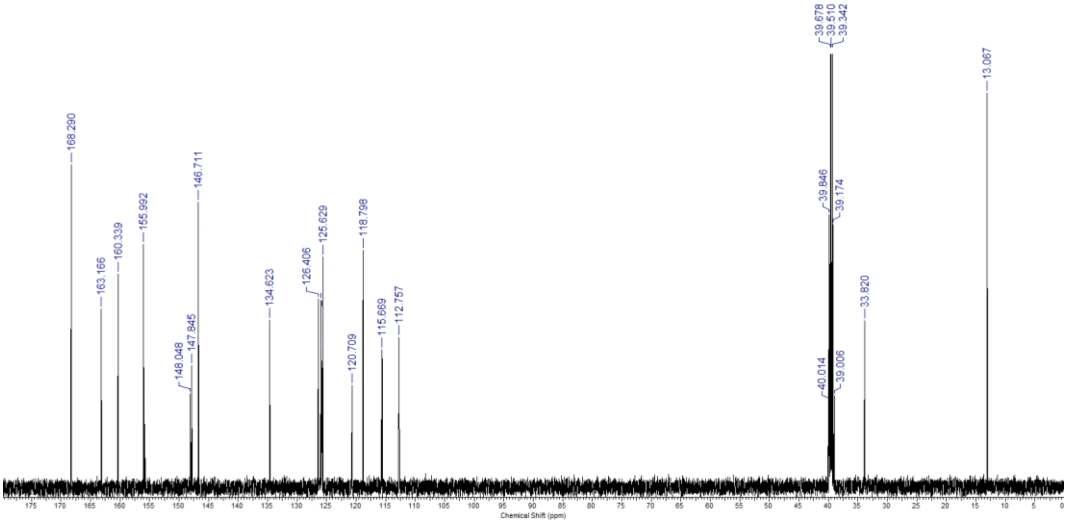

Figure S92. The ^{13}C-NMR spectrum for the compound **5h.**

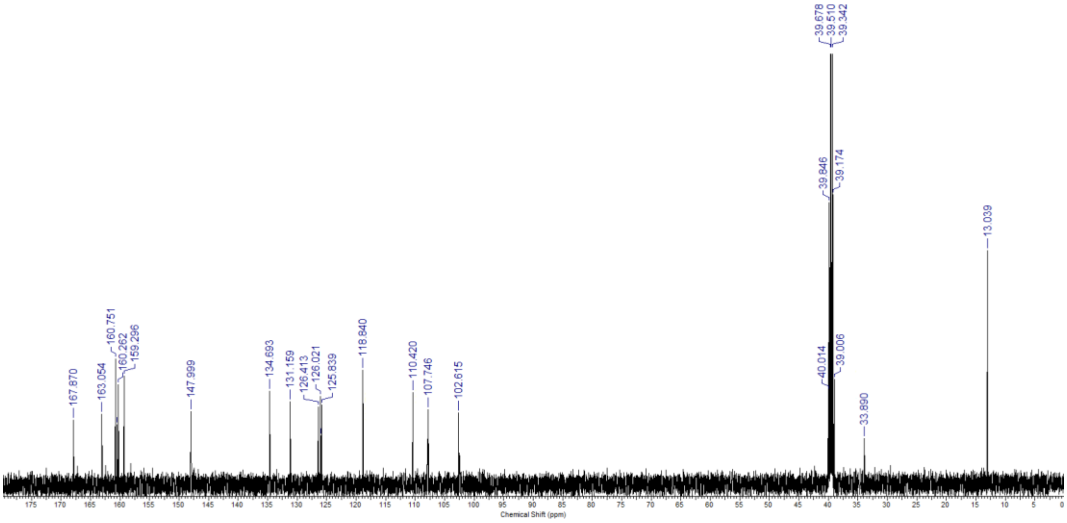

Figure S93. The ^{13}C-NMR spectrum for the compound **5i.**

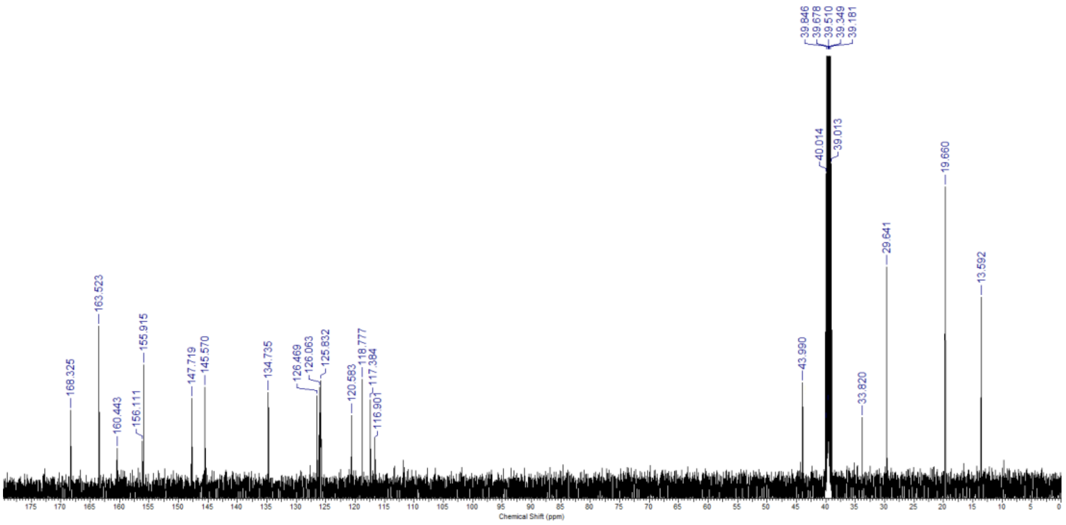

Figure S94. The ^{13}C-NMR spectrum for the compound **5j.**

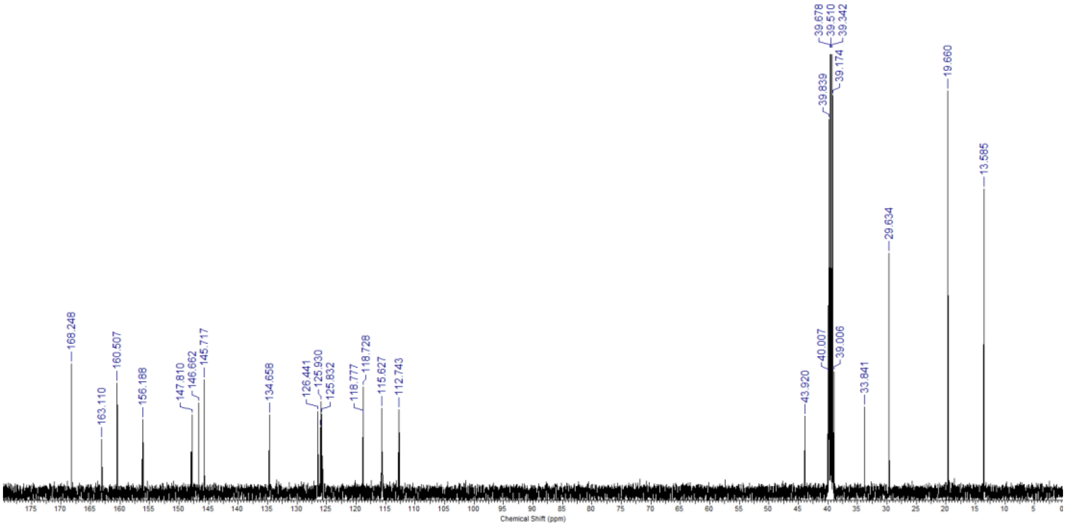

Figure S95. The ^{13}C-NMR spectrum for the compound **5k.**

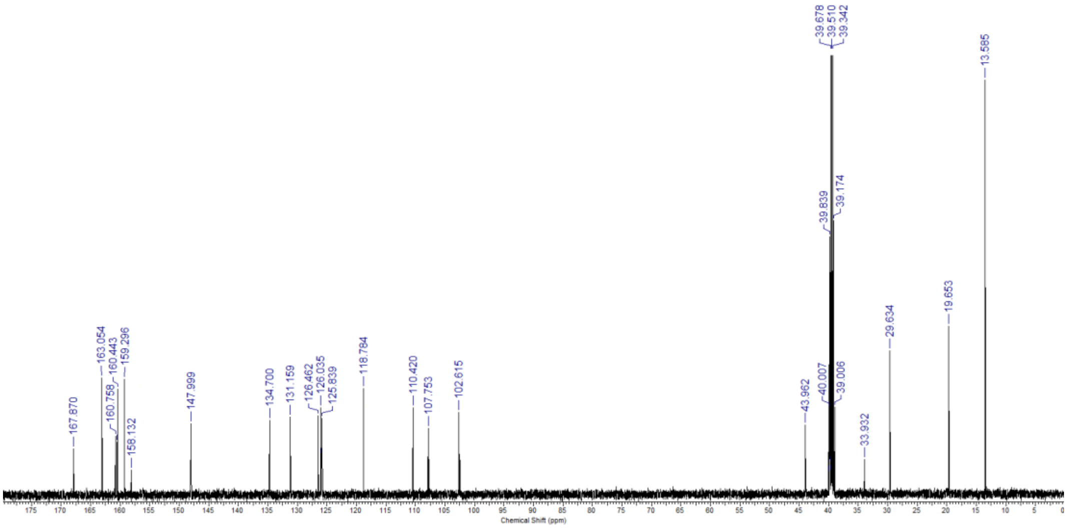

Figure S96. The ^{13}C-NMR spectrum for the compound **5l.**

2. Table

Table S1. Spin density map depending on the phenolic radical for the compounds **5a-l.**

**Compound**	Spin density map depending on the phenolic radical	
**5a**	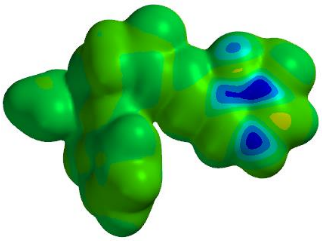 Ortho (H1)	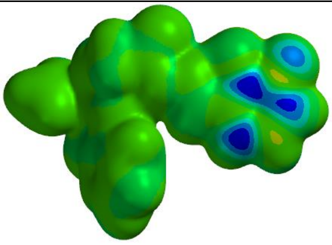 Meta (H2)
**5b**	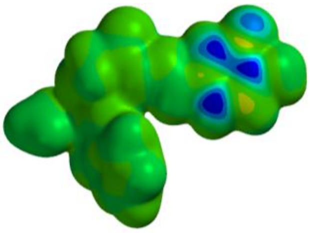 Meta (H2)	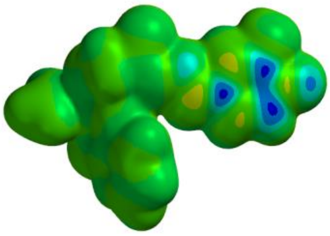 Para (H3)
**5c**	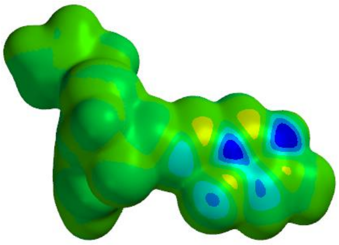 Ortho (H1)	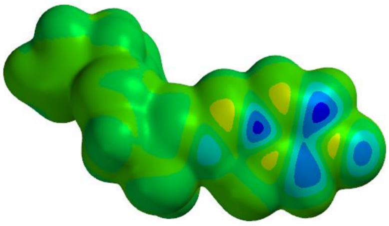 Para (H3)
**5d**	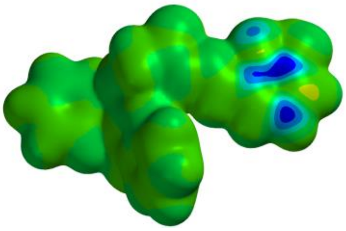 Ortho (H1)	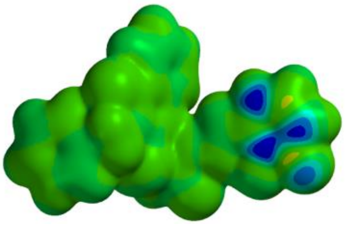 Meta (H2)
**5e**	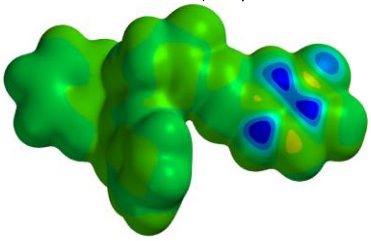 Meta (H2)	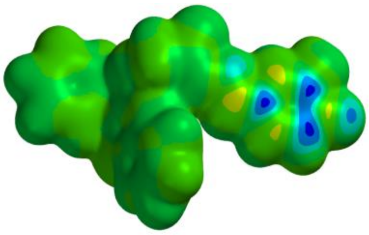 Para (H3)
**5f**	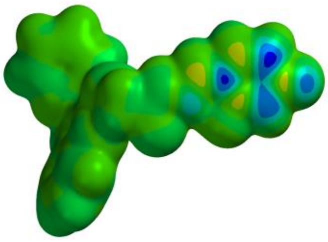 Ortho (H1)	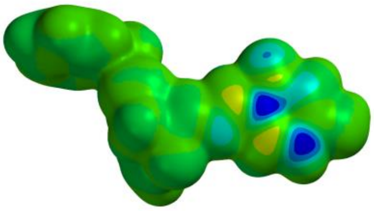 Para (H3)

**Compound**	Spin density map depending on the phenolic radical	
**5g**	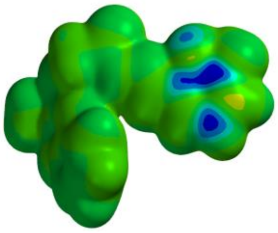 Ortho (H1)	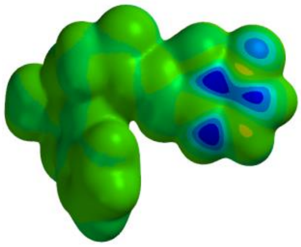 Meta (H2)
**5h**	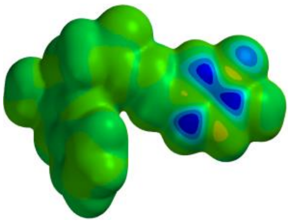 Meta (H2)	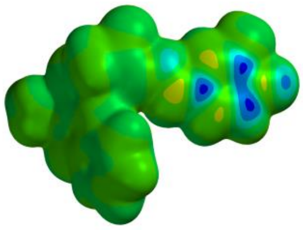 Para (H3)
**5i**	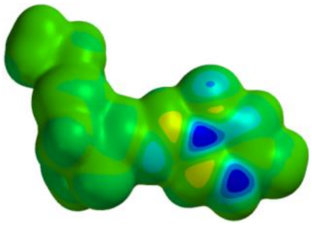 Ortho (H1)	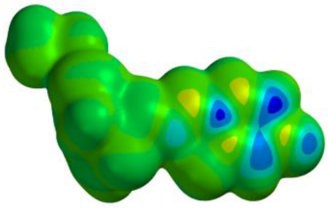 Para (H3)
**5j**	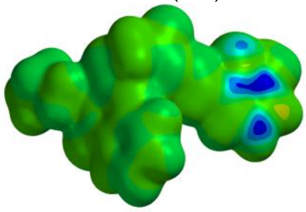 Ortho (H1)	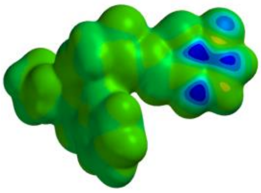 Meta (H2)
**5k**	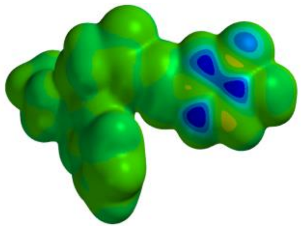 Meta (H2)	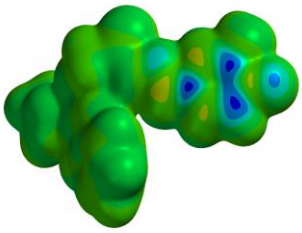 Para (H3)
**5l**	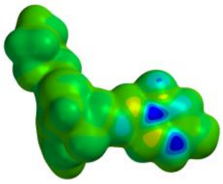 Ortho (H1)	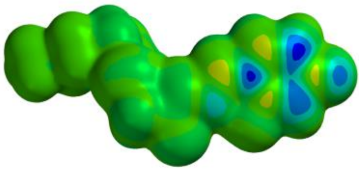 Para (H3)
